# Tricyclic Diterpenoids Selectively Suppress Androgen Receptor-Positive Prostate Cancer Cells

**DOI:** 10.3390/molecules28124743

**Published:** 2023-06-13

**Authors:** Inderpal Sekhon, Guanglin Chen, Keyara Piri, Seiji Shinkawa, Dennis Ashong, Qiang Zhang, Guangdi Wang, Qiao-Hong Chen

**Affiliations:** 1 Department of Chemistry & Biochemistry, California State University, Fresno, CA 93740, USA; 2 Department of Chemistry and RCMI Cancer Research Center, Xavier University of Louisiana, New Orleans, LA 70125, USA

**Keywords:** QW07, diterpenoid, androgen receptor antagonist, *N*-terminal domain, prostate cancer

## Abstract

Androgen receptor (AR) is a viable therapeutic target for lethal castration-resistant prostate cancer (CRPC), because the continued progression of CRPC is mainly driven by the reactivation of AR transcriptional activity. The current FDA-approved AR antagonists binding to ligand binding domain (LBD) become ineffective in CRPC with AR gene amplification, LBD mutation, and the evolution of LBD-truncated AR splice variants. Encouraged by the fact that tricyclic aromatic diterpenoid QW07 has recently been established as a potential *N*-terminal AR antagonist, this study aims to explore the structure–activity relationship of tricyclic diterpenoids and their potential to suppress AR-positive cell proliferation. Dehydroabietylamine, abietic acid, dehydroabietic acid, and their derivatives were selected, since they have a similar core structure as QW07. Twenty diterpenoids were prepared for the evaluation of their antiproliferative potency on AR-positive prostate cancer cell models (LNCaP and 22Rv1) using AR-null cell models (PC-3 and DU145) as comparisons. Our data indicated that six tricyclic diterpenoids possess greater potency than enzalutamide (FDA-approved AR antagonist) towards LNCaP and 22Rv1 AR-positive cells, and four diterpenoids are more potent than enzalutamide against 22Rv1 AR-positive cells. The optimal derivative possesses greater potency (IC_50_ = 0.27 µM) and selectivity than QW07 towards AR-positive 22Rv1 cells.

## 1. Introduction

**Figure 1:**
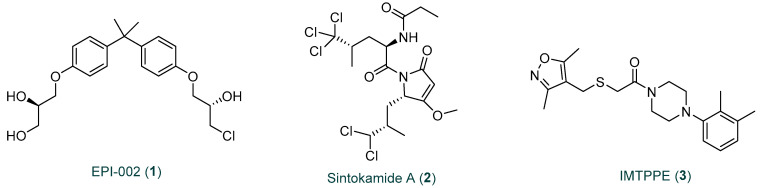
Representative AR NTD antagonists.

The first case of prostate cancer was diagnosed as a then extremely rare disease by J. Adams at the London Hospital in 1853 [1]. It has now evolved as one of the main health concerns for men worldwide, even with the tremendous development of therapeutics and diagnostics [2]. Specifically, prostate cancer is the second most prevalent cancer in men worldwide, with 1.41 million men annually diagnosed as patients with prostate cancer [3]. In the United States, 32,707 prostate cancer deaths were recorded in 2020, accounting for 10.3% of and the second leading cause of the year’s cancer death [4]. One recent concern is raised by the 3% rising incidence per year of prostate cancer from 2014 through 2019 [4]. Castration-resistant prostate cancer (CRPC) is the lethal form of prostate cancer and continues to progress even under a very limited amount of serum androgen [5]. The androgen receptor (AR) transcriptional signal pathway activated by androgen is the driving force for the development and progression of prostate cancer [6]. AR is also a viable therapeutic target for lethal CRPC because the continued progression of CRPC is mainly driven by the reactivation of AR transcriptional activity [5]. AR is a ligand-dependent transcription factor that regulates the specific genes associated with prostate cancer growth and metastasis [7]. Four functional domains of AR are the ***N***-terminal domain (NTD), DNA binding domain (DBD), *C*-terminal ligand binding domain (LBD), and a flexible hinge region connecting LBD to DBD. Three second-generation AR antagonists, including enzalutamide [8], apalutamide [9], and darolutamide [10], have been approved by the U.S. FDA for the treatment of both metastatic and non-metastatic CRPC, as well as castration-sensitive prostate cancer [11]. These three AR antagonists demonstrated appreciable efficacy in prolonging the patient’s survival time, as well as tumor progression time, by binding to the AR LBD and turning the AR switch to “off” status. However, each of them can barely improve median overall survival and is indeed ineffective in CRPC with AR gene amplification, LBD mutation, and the evolution of AR splice variants lacking the LBD. Given that the main resistance mechanisms are centered upon the AR LBD, novel drugs targeting the other functional domains of the AR are thus very likely to be good strategies to treat deadly CRPC. Certain primary resistance mechanisms for current therapeutics that target AR transcriptional axis can be attenuated or circumvented via targeting intrinsically disordered but constitutionally active AR NTD [12]. AR NTD is essential for transcriptional activity of both full-length AR and AR variants lacking the LBD and thus emerges as an attractive but challenging drug target. The *N*-terminal AR antagonists such as EPI-002 (**1**), sintokamide A (**2**), and IMTPPE (**3**) (Figure 1) that were obtained from screening natural compound libraries have been demonstrated to have in vitro capability in inhibiting AR transactivation and AR-positive prostate cancer cell proliferation and in vivo anti-tumor efficacy in CRPC xenograft models [13]. Seeking novel AR antagonists targeting the NTD thus emerges as a promising alternative strategy to fight against the resistance to the current FDA-approved AR antagonists [14].

**Figure 2:**
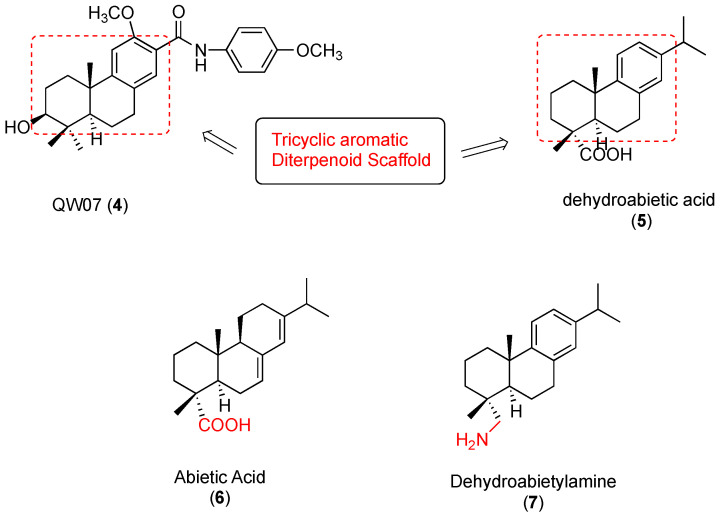
Structures of QW07, dehydroabietic acid, abietic acid, and dehydroabietylamine.

QW07 (**4**, Figure 2), a tricyclic diterpenoid, is a recent addition to AR NTD antagonists, which works by directly binding to AR NTD [15]. QW07 (**4**) blocks the transcriptional activity of AR NTD in both in vitro and in vivo models, suppresses prostate cancer cell proliferation, and shrinks CRPC tumors. QW07 (**4**) was also revealed to inhibit the interactions between the AR and ARE (androgen response elements of DNA), as well as the interaction between the AR and coactivators. QW07 (**4**) has IC_50_ values of 0.50 µM and 0.54 µM towards the PC-3 and DU145 cell lines using SRB cell proliferation assay after 96 h of treatment [16], implying QW07 (**4**) can suppress both AR-positive and AR-negative prostate cancer cell proliferation through AR-dependent and AR-independent pathway. This study aims to dig into tricyclic diterpenoid alkaloids to seek more or even better anti-prostate cancer agents. Some tricyclic aromatic diterpenoids have been illustrated to inhibit prostate cancer cell proliferation [17]. Ferruginol, the simplest phenolic abietane diterpenoid, has shown capability in suppressing PC-3 prostate cancer cell proliferation with an IC_50_ value of 55 µM [18]. Carnosic acid was demonstrated to suppress PC-3 cell proliferation and promote PC-3 and DU145 cell apoptosis [19]. Carnosol has been revealed to inhibit PC-3 prostate cancer cell proliferation with an IC_50_ value of 34 µM [20]. 6-hydroxy-5,6-dehydrosugiol originally isolated from the stem bark of Cryptomeria japonica has been revealed to promote androgen receptor-positive LNCaP and 22RV1 prostate cancer cell apoptosis. The in vitro antiproliferative potency has been confirmed by its in vivo anti-tumor efficacy in xenografted mice models [21].

## 2. Results and Discussion

### 2.1. Design

Since abietic acid (**6**), dehydroabietic acid (**5**), and dehydroabietylamine (**7**) (Figure 2) have a similar tricyclic diterpenoid scaffold as QW07 (**4**, Figure 2), they are chosen as parent tricyclic diterpenoids to synthesize different derivatives for a structure-activity relationship (SAR) study. Additionally, abietic acid (**6**) and dehydroabietylamine (**7**) are commercially available and affordable, which allows the introduction of a wide range of functional groups to systematically investigate the SAR.

### 2.2. Purification and Synthesis

**Scheme 1:**
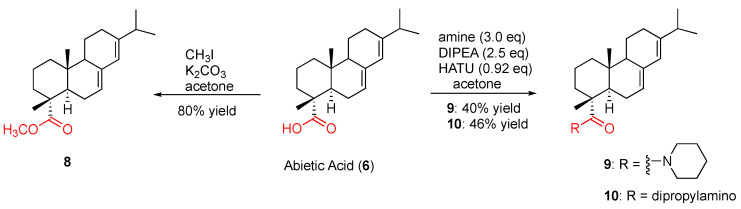
Synthetic scheme for the derivatives of abietic acid.

The less pure abietic acid (**6**, 80% purity) and dehydroabietylamine (**7**, 55% purity) were purchased due to their affordability. Abietic acid was purchased at 80% purity with some aromatic impurities, as indicated by the ^1^H NMR signals at 6–7 ppm. The pure abietic acid was achieved by PTLC purification developing three times with hexane-EtOAc (2:1, *v*/*v*) in 32% for bioassay. The purchased abietic acid with 80% purity was used to make other derivatives. As summarized in Scheme 1, methyl ester **9** was prepared by methylation of abietic acid. Amides **9** and **10** were synthesized by coupling the abietic acid with the appropriate amine.

**Scheme 2:**
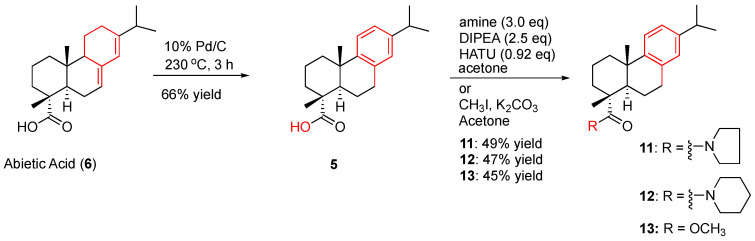
Synthetic scheme for dehydroabietic acid and derivatives.

Dehydroabietic acid (**5**) and derivatives **11**–**13** were prepared according to the procedures illustrated in Scheme 2. Dehydrogenation of abietic acid (**6**) at 230 °C gave **5** with aromatic ring C [22]. Maintaining the temperature at 250 °C, as described in the literature, was difficult since the maximum temperature of the used aluminum beads only reached 230 °C. We, therefore, decided to run the reaction at 230 °C with a prolonged reaction time. Methylation of dehydroabietic acid (**5**) gave methyl ester **13**. Coupling dehydroabietic acid (**5**) with the appropriate amines yielded amides **11** and **12**.

**Scheme 3:**
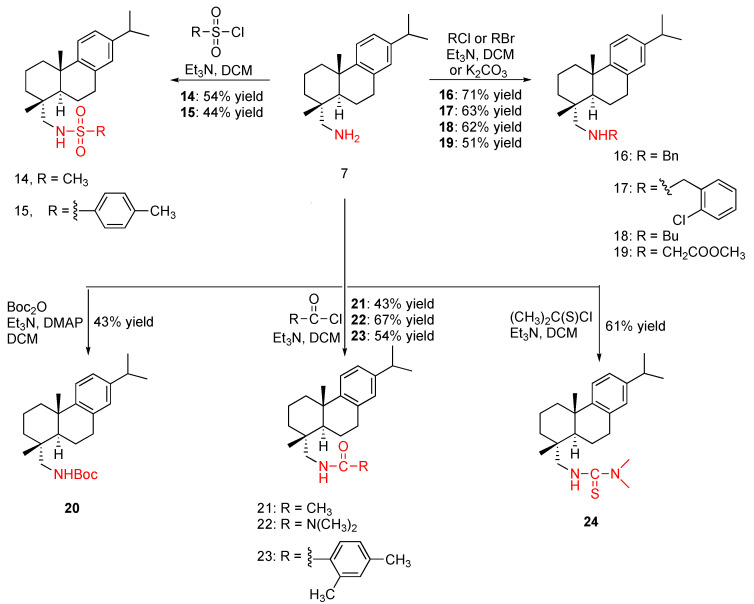
Synthetic scheme for the derivatives of dehydroabietylamine.

Dehydroabietylamine (**7**) is a natural product that was purchased in 55% purity. A crystallization procedure reported by Laaksonen was modified to purify dehydroabietylamine (**7**) [23]. However, a significant amount of the impurities is still observed after this procedure, which was removed by PTLC developing with hexane-EtOAC-triethylamine (1:3:3%, *v*/*v*/*v*) followed by 3% triethylamine in EtOAc. Alternatively, the pure dehydroabietylamine (**7**) was achieved by directly subjecting the 55% dehydroabietylamine to PTLC purification developing with hexane-EtOAC-triethylamine (1:3:3%, *v*/*v*/*v*) followed by 3% triethylamine in EtOAc. The pure dehydroabietylamine (**7**) was only used for bioassay, while the purchased dehydroabietylamine (**7**) in 55% purity was directly used for the preparation of its derivatives. As shown in Scheme 3, sulfonyl derivatives **14** and **15** were prepared by treating dehydroabietylamine with either mesyl chloride or tosyl chloride mediated by triethylamine. *N*-alkyl derivatives **16**ߝ**19** were obtained by *N*-alkylation of dehydroabietylamine (**7**) with the appropriate alkyl halide mediated by triethylamine or potassium carbonate. Carbamoyl derivative **22** and thiocarbamoyl derivative **24** were prepared by treating dehydroabietylamine (**7**) with *N*, *N*-dimethyl(thio)carbamoyl chloride using triethylamine as a base. Amides **21** and **23** were synthesized by reacting dehydroabietylamine with the appropriate acetyl chloride. It is worth noting that the yields (43–71%) for the above-mentioned derivatives are not very high, mainly due to the challenging process of completely removing the impurities from the purchased dehydroabietylamine (55% purity). The yields could be appreciably increased by using the pure version of dehydroabietylamine (**7**).

### 2.3. Antiproliferative Activity

**Figure 3:**
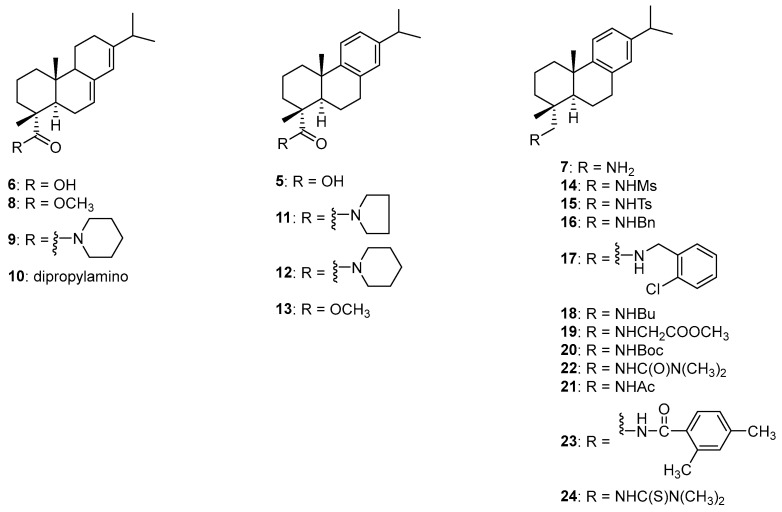
Chemical structures of tricyclic diterpenoids.

To initiate the exploration of the structure-activity relationship of the tricyclic diterpenoids, twenty tricyclic diterpenoids, including abietic acid (**6**), dehydroabietic acid (**5**), dehydroabietylamine (**7**), and their derivatives (**8**–**24**) (Figure 3), have been evaluated for their antiproliferative potency on AR-positive prostate cancer cell lines (LNCaP and 22Rv1) using AR-null cell models (PC-3 and DU145) as comparisons. The critical difference between the two AR-positive cell lines is that LNCaP only includes full-length AR with androgen responsiveness, while 22Rv1 consists of AR-V7 that lacks ligand binding domain and androgen responsiveness. WST-1 cell proliferation assay is used in this study due to the water solubility and stability of the tetrazolium dye [24,25]. The FDA-approved AR antagonist enzalutamide was used as a positive control.

As illustrated in Table 1, six tricyclic diterpenoid compounds, **7**, **10**, **14**, **18**, **19**, and **24**, possess greater potency than enzalutamide towards LNCaP and 22Rv1 AR-positive prostate cancer cells. Four tricyclic diterpenoid compounds, **5**, **16**, **21**, and **22,** are more potent than enzalutamide against 22Rv1 AR-positive prostate cancer cells. Abietic acid (**6**) selectively inhibits AR-positive LNCaP prostate cancer cell proliferation. The potency of AR-positive cells can be increased by appropriate modification of the 4-carboxyl group (e.g., **10**). Dehydroabietic acid (**5**) selectively suppresses both LNCaP and 22RV1 prostate cancer cell proliferation. Therefore, the difference in antiproliferative potency of abietic acid (**6**) and dehydroabietic acid (**5**) suggests that the induction of the aromatic ring C enhances the selectivity towards the 22Rv1 castration-resistance prostate cancer cell model. However, the potency is very moderate, even with selectivity. The incorporation of a bulky group to the 4-carboxyl group of dehydroabietic acids, such as **11,** loses selectivity. Attaching piperidine to the 4-carboxyl group of dehydroabietic acid (**12**) eliminates the potency of LNCaP and 22Rv1 cells.

Dehydroabietylamine (**7**) has greater potency than abietic acid (**6**) towards both AR-positive and AR-negative prostate cancer cells, with their IC_50_ values falling into the range of 19.30–40.22 µM. The introduction of an n-butyl group to the amine moiety in compound **18** significantly enhances the antiproliferative potency towards AR-positive prostate cancer cells, especially towards 22Rv1, with an IC_50_ value of 0.27 µM. The selectivity of antiproliferative potency towards the 22Rv1 prostate cancer cells over that against the AR-null PC-3 prostate cancer cell lines is 89-fold. This optimal derivative **18** possesses greater potency (IC_50_ = 0.27 µM) and selectivity than QW07 towards AR-positive 22Rv1 cells.

## 3. Conclusions

Dehydroabietylamine (**7**), abietic acid (**6**), dehydroabietic acid (**5**), and their derivatives **8**–**24** were purified or synthesized for evaluation on both AR-positive and AR-null prostate cancer cell models since they have a similar core structure as QW07. Twenty diterpenoids were prepared for the evaluation of their antiproliferative potency on AR-positive prostate cancer cell models (LNCaP and 22Rv1) using AR-negative cell models (PC-3 and DU145) as comparisons. Our data indicated that (i) six tricyclic diterpenoids, **7**, **10**, **14**, **18**, **19**, and **24,** possess greater potency than enzalutamide (FDA-approved AR antagonist) towards LNCaP and 22Rv1 AR-positive cells, and (ii) four tricyclic diterpenoids, **5**, **16**, **21**, and **22**, are more potent than enzalutamide against 22Rv1 AR-positive cells. The optimal amine derivative **18** possesses greater potency (IC_50_ = 0.27 µM) and selectivity than QW07 towards AR-positive 22Rv1 cells. These data warrant the further exploration of tricyclic diterpenoids for potential treatment of prostate cancer.

## 4. Materials and Methods

### 4.1. General Experiments

A Thermo Scientific Q-Exactive mass spectrometer with electrospray ionization (ESI) was utilized to obtain the HRMS. A Nicolet Nexus 470 FTIR spectrophotometer (Waltham, MA, USA) was used to gather the IR spectra. A Bruker Fourier 300 spectrometer was employed to acquire NMR spectra, with CDCl_3_ as the solvent. The chemical shifts of the NMR spectra are reported in ppm, with reference to the corresponding solvent peak, while the coupling constants are expressed in Hz. The PureSolv MD 7 Solvent Purification System from Innovative Technologies (MB-SPS-800) (Herndon, VA, USA) or activated molecular sieves (heating at 180–200 °C for 6 h under vacuum) were used to remove the trace amount of water from acetone and dichloromethane. All remaining reagents and solvents were directly used as received from commercial sources. All column chromatography was carried out on silica gel with a particle size of 32–63 μm. Preparative thin-layer chromatography (PTLC) purifications were conducted on silica gel 60 GF254-loaded plates (EMD Millipore Corporation, Burlington, MA, USA). Abietic acid (80% purity) and dehydroabietylamine (55% purity) were purchased from Fisher Scientific (Portland, OR, USA). All NMR spectra and high-resolution mass spectra were included in Supplementary Materials.

### 4.2. Purification of Abietic Acid (**6**)

The purchased abietic acid (**6**) has only 80% purity, which was purified via PTLC eluting twice with hexane-ethyl acetate (2:1, *v*/*v*) to give the pure abietic acid as a yellow oil. The recovery rate is 66%. ^1^H NMR (300 MHz, CDCl_3_) *δ* 5.77 (s, 1H), 5.37 (s, 1H), 2.26–2.18 (m, 1H), 2.10–2.03 (m, 4H), 1.97–1.76 (m, 5H), 1.62–1.55 (m, 2H), 1.28–1.09 (m, 3H), 1.25 (s, 3H), 1.01 (d, *J* = 6.9 Hz, 3H), 1.00 (d, *J* = 6.9 Hz, 3H), 0.83 (s, 3H). ^13^C NMR (75 MHz, CDCl_3_) *δ* 184.69, 145.00, 135.28, 122.12, 120.24, 50.65, 46.05, 44.61, 37.98, 36.89, 34.60, 34.18, 27.17, 25.32, 22.19, 21.13, 20.59, 17.77, 16.43, 13.75. HRMS (ESI): *m*/*z* calculated for C_20_H_31_O_2_ [M + H]^+^: 303.2324. Found: 303.2321. IR (film) *v*_max_: 3395–2600, 2951, 1686, 1277, 1154, 993, 891,789 cm^−1^.

### 4.3. Synthesis of **8**

To a solution of abietic acid (5.00 g, 80%, 13.2 mmol) in acetone (25 mL) at room temperature were sequentially added K_2_CO_3_ (2.51 g, 18.2 mmol) and methyl iodide (1.51 mL, 24.3 mmol) dropwise. The reaction was then stirred at room temperature for two days and monitored with TLC (hexane-ethyl acetate, 3:1, *v*/*v*) for completeness. The solution was diluted with ethyl acetate (300 mL) and rinsed with brine (30 mL × 5). The organic fraction was dried over anhydrous Na_2_SO_4_ and concentrated. The crude product was purified through column chromatography, eluting with hexane-ethyl acetate (3:1, *v*/*v*) to give the desired product an 80% yield as a yellowish oil. ^1^H NMR (300 MHz, CDCl_3_) *δ* 5.77 (s, 1H), 5.36 (s, 1H), 3.63 (s, 3H), 2.26–2.17 (m, 1H), 2.13–2.03 (m, 4H), 1.91–1.70 (m, 5H), 1.62–1.57 (m, 2H), 1.27–1.19 (m, 3H), 1.25 (s, 3H), 1.01 (d, *J* = 6.9 Hz, 3H), 1.00 (d, *J* = 6.9 Hz, 3H), 0.82 (s, 3H). ^13^C NMR (75 MHz, CDCl_3_) *δ* 178.66, 144.95, 135.18, 122.00, 120.25, 51.52, 50.60, 46.25, 44.76, 37.98, 36.77, 34.54, 34.19, 27.13, 25.33, 22.12, 21.51, 17.79, 16.67, 13.68. HRMS (ESI): *m*/*z* calculated for C_21_H_33_O_2_ [M + H]^+^: 317.2480. Found: 317.2478. IR (film) *v*_max_: 2928, 2868, 1724, 1459, 1385, 1243, 1185, 1106 cm^−1^.

### 4.4. Synthesis of **9**

Piperidine (89 μL, 0.9 mmol) was added to a solution of abietic acid (113 mg, 80% purity, 0.3 mmol) in acetone (5 mL) under argon at 0 °C, to which was added a solution of DIPEA (0.13 mL, 0.75 mmol) and HATU (105 mg, 0.28 mmol) in acetone (5 mL). The resulting reaction mixture was stirred at 0 °C for 15 to 20 min, when it turned to a yellow color. The reaction was then allowed to proceed at room temperature overnight prior to removing the solvent. The residue was diluted with EtOAc (75 mL), which was rinsed with brine (25 mL × 3). The EtOAc layer was dried over anhydrous Na_2_SO_4_ and concentrated to afford a yellow oil, which was subjected to PTLC purification eluting with hexane/EtOAc (2:1, *v*/*v*) to give the desired product in 40% yield as a yellow oil. ^1^H NMR (300 MHz, CDCl_3_) *δ* 5.74 (s, 1H), 5.35 (s, 1H), 3.58 (t, *J* = 5.6 Hz, 4H), 2.24–2.14 (m, 2H), 2.07–2.02 (m, 2H), 1.89–1.75 (m, 5H), 1.67–1.45 (m, 10H), 1.30 (s, 3H), 1.26–1.17 (s, 2H), 0.99 (d, *J* = 6.9 Hz, 3H), 0.98 (d, *J* = 6.9 Hz, 3H), 0.84 (s, 3H). ^13^C NMR (75 MHz, CDCl_3_) *δ* 176.04, 144.41, 134.83, 122.27, 120.97, 51.01, 46.92, 46.12, 44.01, 37.48, 35.04, 34.44, 27.03, 25.89, 25.54, 24.43, 22.25, 21.03, 20.46, 19.96, 18.17, 13.79. HRMS (ESI): *m*/*z* calculated for C_25_H_40_NO [M + H]^+^: 370.3110. Found: 370.3106. IR (film) *v*_max_: 2931, 2853, 1681, 1416, 1247, 1040 cm^−1^.

### 4.5. Synthesis of **10**

Dipropylamine (123 μL, 0.9 mmol) was added to a solution of abietic acid (113 mg, 80% purity, 0.3 mmol) in acetone (5 mL) under argon at 0 °C, to which was added a solution of DIPEA (0.13 mL, 0.75 mmol) and HATU (105 mg, 0.28 mmol) in acetone (5 mL). The resulting reaction mixture was stirred at 0 °C for 15 to 20 min, when it turned to a yellow color. The reaction was then allowed to proceed at room temperature overnight prior to removing the solvent. The residue was diluted with EtOAc (75 mL), which was rinsed with brine (25 mL × 3). The EtOAc layer was dried over anhydrous Na_2_SO_4_ and concentrated to afford a crude product, which was purified with PTLC eluting with hexane/EtOAc (6:1, *v*/*v*) to give **10** as a red oil in 46% yield. ^1^H NMR (300 MHz, CDCl_3_) *δ* 5.75 (s, 1H), 5.36 (s, 1H), 3.46–3.13 (m, 4H), 2.85–2.74 (m, 1H), 2.33–1.99 (m, 6H), 1.86–1.73 (m, 6H), 1.62–1.44 (m, 8H), 1.30 (s, 3H), 1.25–1.17 (m, 4H), 1.00 (d, *J* = 6.9 Hz, 3H), 0.99 (d, *J* = 6.9 Hz, 3H), 0.92–0.82 (overlapped, 9H). ^13^C NMR (75 MHz, CDCl_3_) *δ* 177.12, 144.89, 135.35, 122.70, 121.41, 51.65, 50.63, 46.96, 44.63, 38.17, 35.70, 34.96, 27.51, 25.97, 24.06, 22.74, 21.50, 20.95, 20.25, 18.72, 14.31, 11.44. HRMS (ESI): *m*/*z* calculated for C_26_H_44_NO [M + H]^+^: 386.3423. Found: 386.3423. IR (film) *v*_max_: 2931, 1693, 1506, 1471, 1385, 1100 cm^−1^.

### 4.6. Synthesis of Dehydroabietic Acid (**5**)

Abietic acid (502 mg, 80%, 1.33 mmol) and 10% Pd/C (12.6 mg) were added to a 5 mL conical vial with a triangular spin vane. The reaction mixture was heated under argon using aluminum beads to 220–230 °C (the melting point of abietic acid is 250 °C) for four hours. TLC (hexane/EtOAc, 4:1) was used to check the completeness of the reaction. The reaction mixture was then cooled down to room temperature and washed with ethyl acetate (10 mL) before it completely solidified. The black solids were placed in a celite pad and rinsed with ethyl acetate (10 mL). The combined ethyl acetate fractions were concentrated to give a yellow solid, which was purified with column chromatography eluting with hexane/EtOAC (4:1, *v*/*v*) to give dehydroabietic acid in 66% yield as a clear crystal solid. ^1^H NMR (300 MHz, CDCl_3_) *δ* 7.19 (d, *J* = 8.1 Hz, 1H), 7.02 (d, *J* = 8.1 Hz, 1H), 6.92 (s, 1H), 2.98–2.80 (m, 3H), 2.35–2.26 (m, 2H), 1.97–1.72 (m, 5H), 1.61–1.52 (m, 2H), 1.31 (s, 3H), 1.25 (d, *J* = 6.9 Hz, 6H), 1.25 (s, 3H). ^13^C NMR (75 MHz, CDCl_3_) *δ* 185.60, 146.89, 145.86, 134.83, 127.04, 124.25, 124.03, 47.58, 44.70, 38.04, 36.99, 36.88, 33.59, 30.14, 25.26, 24.12, 21.91, 18.67, 16.33. HRMS (ESI): *m*/*z* calculated for C_20_H_29_O_2_ [M + H]^+^: 301.2167. Found: 301.2164. IR (film) *v*_max_: 3047, 2954, 2928, 2867, 1690, 1612, 1458, 1276, 1134 cm^−1^.

### 4.7. General Synthesis Procedures of Amides (**11** and **12**)

The corresponding amine (3 equv.) was added to a solution of dehydroabietic acid in half the volume of acetone (the concentration of the limiting reagent in acetone is 0.03 M) under argon at 0 °C. A solution of DIPEA (2.5 equiv.) and HATU (0.92 equiv.) in the remaining acetone was then added at 0 °C. The resulting reaction mixture was stirred for 15 to 20 min when it turned a yellow color. Then the reaction was allowed to proceed at room temperature overnight. After the removal of the organic solvent, the residue was diluted with 75 mL of ethyl acetate, which was rinsed with brine (25 mL × 3). The ethyl acetate layer was dried over anhydrous Na_2_SO_4_ and concentrated to yield a crude product as a yellow oil.

#### 4.7.1. Amide **11**

The crude product was subjected to PTLC purification eluting with hexane/EtOAC (4:1, *v*/*v*) to give amide **11** in 49% yield as a colorless solid. ^1^H NMR (300 MHz, CDCl_3_) *δ* 7.17 (d, *J* = 8.1 Hz, 1H), 6.99 (d, *J* = 8.1 Hz, 1H), 6.89 (s, 1H), 3.57 (t, *J* = 6.9 Hz, 4H), 3.01– 2.78 (m, 3H), 2.39 (dd, *J* = 12.0, 2.1 Hz, 1H), 2.29 (d, *J* = 13.2 Hz, 1H), 1.86–1.69 (m, 9H), 1.60–1.40 (m, 2H), 1.35 (s, 3H), 1.25 (s, 3H), 1.22 (d, *J* = 6.9 Hz, 6H). ^13^C NMR (75 MHz, CDCl_3_) *δ* 176.84, 147.37, 145.63, 135.15, 127.10, 124.21, 123.85, 48.84, 47.52, 44.29, 37.91, 37.45, 34.59, 33.53, 30.47, 25.64, 24.11, 24.08, 21.86, 18.93, 18.18. HRMS (ESI): *m*/*z* calculated for C_24_H_36_NO [M + H]^+^: 354.2797. Found: 354.2796. IR (film) *v*_max_: 2953, 2867, 1711, 1609, 1497, 1279, 1036 cm^−1^. HRMS (ESI): *m*/*z* calculated for C_24_H_36_NO [M + H]^+^: 354.2797. Found: 354.2796.

#### 4.7.2. Amide **12**

The crude product was subjected to PTLC purification eluting with hexane-EtOAc (4:1, *v*/*v*) to afford amide **12** as a clear oil in 47% yield. ^1^H NMR (300 MHz, CDCl_3_) *δ* 7.16 (d, *J* = 8.3 Hz, 1H), 6.99 (d, *J* = 8.3 Hz, 1H), 6.90 (s, 1H), 3.65–3.51 (m, 4H), 3.06–2.76 (m, 1H), 2.90–2.76 (m, 2H), 2.37–2.25 (m, 2H), 1.84–1.70 (m, 5H), 1.65–1.58 (m, 3H), 1.55–1.41 (m, 5H), 1.34 (s, 3H), 1.25 (s, 3H), 1.23 (d, *J* = 6.9 Hz, 6H). ^13^C NMR (75 MHz, CDCl_3_) *δ* 177.02, 147.25, 145.62, 135.36, 127.19, 124.24, 123.80, 47.20, 46.88, 45.40, 37.81, 37.60, 35.33, 33.55, 30.81, 26.37, 25.67, 24.89, 24.11, 24.08, 22.26, 19.05. HRMS (ESI): *m*/*z* calculated for C_25_H_38_NO [M + H]^+^: 368.2953. Found: 368.2948. IR (film) *v*_max_: 2935, 1768, 1615, 1464, 1262, 1007 cm^−1^.

### 4.8. Methylation of Dehydroabietic Acid (Synthesis of **13**)

To a solution of dehydroabietic acid (97 mg, 0.32 mmol) in acetone (3.2 mL, 0.1 M) was added K_2_CO_3_ (133 mg, 0.96 mmol), and the reaction mixture was stirred for 5 min before adding methyl iodide (0.09 mL, 1.45 mmol). The reaction was allowed to proceed with stirring under argon at room temperature overnight when the reaction was completed as monitored by TLC (hexane/EtOAc, 5:1). After removing the acetone; the crude product was diluted with 50 mL of ethyl acetate and rinsed with brine (10 mL × 5). The organic layer was dried over anhydrous Na_2_SO_4_ and concentrated to give a crude product, which was subjected to PTLC purification eluting with hexane/EtOAc (5:1) to give the desired product as a white solid in 45% yield. ^1^H NMR (300 MHz, CDCl_3_) *δ* 7.19 (d, *J* = 8.3 Hz, 1H), 7.02 (dd, *J* = 8.3, 2.4 Hz, 1H), 6.91 (d, *J* = 2.4 Hz, 1H), 3.68 (s, 3H), 2.94–2.80 (m, 3H), 2.35–2.29 (m, 1H), 2.27 (dd, *J* = 12.6, 2.4 Hz, 1H), 1.93–1.64 (m, 5H), 1.57–1.40 (m, 2H), 1.30 (s, 3H), 1.25 (d, *J* = 6.9 Hz, 6H), 1.23 (s, 3H). ^13^C NMR (75 MHz, CDCl_3_) *δ* 179.24, 147.03, 145.82, 134.80, 126.99, 124.25, 124.02, 52.05, 47.77, 44.97, 38.11, 37.05, 36.75, 33.58, 30.12, 25.22, 24.11, 21.83, 18.69, 16.62. HRMS (ESI): *m*/*z* calculated for C_21_H_31_O_2_ [M + H]^+^: 315.2324. Found: 315.2321. IR (film) *v*_max_: 2928, 2867, 1725, 1611, 1432, 1243, 1057 cm^−1^.

### 4.9. Purification of Dehydroabietylamine (**7**)

The purchased dehydroabietylamine (204.5 mg, 55% purity) was purified via PTLC developing with hexane-EtOAc (1:3 with 3% Et_3_N, *v*/*v*) three times to remove the impurity that is on top of the desired product. Pure dehydroabietylamine (**7**) was obtained in 31% yield as a clear oil. ^1^H NMR (300 MHz, CDCl_3_) *δ* 7.19 (d, *J* = 8.1, 1H), 7.00 (d, *J* = 8.1, 1H), 6.90 (d, *J* = 2.1 Hz, 1H), 2.92–2.78 (m, 3H), 2.62 (d, *J* = 13.2 Hz, 1H), 2.47 (d, *J* = 13.2 Hz, 1H), 2.38 (br.s, 2H), 2.30 (br.d, *J* = 14.1 Hz, 1H), 1.79–1.64 (m, 4H), 1.53–1.34 (m, 4H), 1.23 (s, 3H), 1.23 (d, *J* = 6.9 Hz, 6H), 0.92 (s, 3H). ^13^C NMR (75 MHz, CDCl_3_) *δ* 147.49, 145.58, 134.73, 126.87, 124.31, 123.90, 53.81, 45.01, 38.59, 37.45, 37.22, 35.30, 33.51, 30.23, 25.33, 24.08, 24.05, 18.79, 18.72. HRMS (ESI): *m*/*z* calculated for C_20_H_32_N [M + H]^+^: 286.2535. Found: 286.2530. IR (film) *v*_max_: 3305, 2922, 2865, 2085, 1611, 1555, 1497, 1237, 1173, 1058, 908 cm^−1^.

### 4.10. Synthesis of **14**

To a solution of dehydroabietylamine (156 mg, 55% purity, 0.3 mmo) in DCM (3 mL) was added triethylamine (104 μL, 0.75 mmol) at 0 °C. The solution was stirred for 30 min before adding mesyl chloride (23 μL, 0.3 mmol). The reaction was allowed to proceed at room temperature under argon overnight before diluting with EtOAc (75 mL). The resulting mixture was rinsed with brine (10 mL × 2), dried over anhydrous Na_2_SO_4_, and concentrated. The yellow crude oil was subjected to PTLC purification developing four times with hexane/EtOAc (3:1, *v*/*v*) to give the desired product as a white crystal in 54% yield. ^1^H NMR (300 MHz, CDCl_3_) *δ* 7.17 (d, *J* = 8.4 Hz, 1H), 6.99 (dd, *J* = 8.4, 2.4 Hz, 1H), 6.89 (d, *J* = 2.1 Hz, 1H), 4.69 (t, *J* = 6.9 Hz, 1H), 3.04–2.78 (m, 5H), 2.89 (s, 3H, SO_2_*CH_3_*), 2.29 (d, *J* = 12.6 Hz, 1H), 1.7–1.65 (m, 4H), 1.52 (dd, *J* = 10.8, 3.9 Hz, 1H), 1.44–1.30 (m, 3H), 1.23 (d, *J* = 6.9 Hz, 6H, CH(CH_3_)_2_), 1.22 (s, 3H, CH_3_), 0.95 (s, 3H). ^13^C NMR (75 MHz, CDCl_3_) *δ* 147.04, 145.82, 134.66, 126.96, 124.26, 123.98, 45.02, 40.17, 38.35, 37.49, 37.10, 35.92, 33.55, 29.98, 25.31, 24.12, 24.09, 18.89, 18.61, 18.56. HRMS (ESI): *m*/*z* calculated for C_21_H_34_NO_2_S [M + H]^+^: 364.2310. Found: 364.2303. IR (film) *v*_max_: 3255, 3000, 2959, 2927, 2871, 1496, 1433, 1375, 1232, 1134, 1050 cm^−1^.

### 4.11. Synthesis of **15**

To a solution of dehydroabietylamine (156 mg, 55% purity, 0.3 mmo) in DCM (3 mL) was added triethylamine (104 μL, 0.75 mmol) at 0 °C. The solution was stirred for 30 min before adding 4-tolenesulfonyl chloride (57 mg, 0.3 mmol). The reaction was allowed to proceed at room temperature under argon for 5 h before diluting with EtOAc (75 mL). The resulting mixture was rinsed with brine (10 mL × 2), dried over anhydrous Na_2_SO_4_, and concentrated. The crude product is purified with column chromatography eluting with hexane-EtOAc (5:1, *v*/*v*) followed by further PTLC purification developing four times with hexane-EtOAc (8:1, *v*/*v*) to give **15** as a clear oil in 44% yield. ^1^H NMR (300 MHz, CDCl_3_) *δ* 7.74 (d, *J* = 8.4 Hz, 2H), 7.24 (d, *J* = 8.4 Hz, 2H), 7.14 (d, *J* = 8.1 Hz, 1H), 6.98 (dd, *J* = 8.1, 2.4 Hz, 1H), 6.87 (d, *J* = 2.4 Hz,1H), 4.87 (t, *J* = 7.2 Hz, 1H, N*H*), 2.87–2.77 (m, 4H), 2.64 (dd, *J* = 12.9, 7.5 Hz, 1H), 2.40 (s, 3H, Ar-CH_3_), 2.24 (br.d, *J* = 12.9 Hz, 1H), 1.74–1.60 (m, 4H), 1.53–1.48 (m, 1H), 1.34–1.26 (m, 3H), 1.23 (d, *J* = 6.9 Hz, 6H), 1.18 (s, 3H), 0.88 (s, 3H). ^13^C NMR (75 MHz, CDCl_3_) *δ* 147.07, 145.66, 143.33, 137.19, 134.76, 129.81, 127.08, 126.93, 124.22, 123.88, 53.89, 44.92, 38.29, 37.47, 37.04, 35.83, 33.55, 29.95, 25.31, 24.15, 21.61, 18.80, 18.66, 18.60. HRMS (ESI): *m*/*z* calculated for C_27_H_38_NO_2_S [M + H]^+^: 440.2623. Found: 440.2616. IR (film) *v*_max_: 3273, 3273, 2929, 2851, 2448, 2216, 2182, 1952, 1459, 1093 cm^−1^.

### 4.12. Synthesis of **16**

To a solution of dehydroabietylamine (156 mg, 55% purity, 0.3 mmo) in DCM (3 mL) was added triethylamine (104 μL, 0.75 mmol) at 0 °C. The solution was stirred for 30 min before adding benzyl bromide (36 μL, 0.3 mmol). The reaction was allowed to proceed at room temperature under argon for 5 h before diluting with EtOAc (75 mL). The resulting mixture was rinsed with brine (10 mL × 2), dried over anhydrous Na_2_SO_4_, and concentrated. The clear crude oil is subjected to PTLC purification by developing twice with hexane/EtOAc (4:1, *v*/*v*) to give the desired product as wax in 71% yield. ^1^H NMR (300 MHz, CDCl_3_) *δ* 7.40–7.27 (m, 5H), 7.17 (d, *J* = 8.1 Hz, 1H), 6.97 (dd, *J* = 8.1, 2.4 Hz, 1H), 6.88 (d, *J* = 2.4 Hz, 1H), 3.84 (s, 2H), 2.88–2.77 (m, 3H), 2.54 (d, *J* = 12.0 Hz, 1H), 2.37 (d, *J* = 12.0 Hz, 1H), 2.27 (br.d, *J* = 12.6 Hz, 1H), 1.75–1.63 (m, 4H), 1.50–1.36 (m, 4H), 1.22 (d, *J* = 6.9 Hz, 6H), 1.21 (s, 3H), 0.95 (s, 3H). ^13^C NMR (75 MHz, CDCl_3_) *δ* 147.53, 145.55, 134.87, 128.52, 127.31, 126.89, 124.34, 123.90, 60.33, 54.26, 45.52, 38.48, 37.55, 37.06, 33.53, 30.27, 25.49, 24.11, 19.31, 18.93. HRMS (ESI): *m*/*z* calculated for C_27_H_38_N [M + H]^+^: 376.3004. Found: 376.3001. IR (film) *v*_max_: 2923, 2866, 1538, 1495, 1361, 1264, 1173, 1075, 971 cm^−1^.

### 4.13. Synthesis of **17**

To a solution of dehydroabietylamine (156 mg, 55% purity, 0.3 mmo) in DCM (3 mL) was added triethylamine (104 μL, 0.75 mmol) at 0 °C. The solution was stirred for 30 min before adding 2-chlorobenzyl bromide (62 mg, 0.3 mmol). The reaction was allowed to proceed at room temperature under argon for 4 h before diluting with EtOAc (75 mL). The resulting mixture was rinsed with brine (10 mL × 2), dried over anhydrous Na_2_SO_4_, and concentrated. The clear crude oil was purified via PTLC, developing twice with hexane/EtOAc (7:1, *v*/*v*) to yield the desired product as a clear oil in 63% yield. ^1^H NMR (300 MHz, CDCl_3_) *δ* 7.45 (dd, *J* = 7.2, 2.1 Hz, 1H), 7.40 (dd, *J* = 7.2, 1.8 Hz, 1H), 7.31–7.21 (overlapped, 3H), 7.04 (dd, *J* = 8.1, 2.1 Hz, 1H), 6.94 (d, *J* = 2.1 Hz, 1H), 3.95 (d, *J* = 14.1 Hz, 1H, benzylic H), 3.88 (d, *J* = 14.1 Hz, 1H, benzylic H), 2.95–2.83 (m, 3H), 2.58 (d, *J* = 11.7 Hz, 1H), 2.28 (d, *J* = 11.7 Hz, 1H), 2.34–2.30 (overlapped, 1H), 1.87–1.66 (m, 5H), 1.60–1.41 (m, 3H), 1.28 (d, *J* = 6.9 Hz, 6H), 1.27 (s, 3H), 0.98 (s, 3H). ^13^C NMR (75 MHz, CDCl_3_) *δ* 147.67, 145.55, 138.32, 134.96, 133.76, 130.15, 129.51, 128.19, 126.92, 126.79, 124.45, 123.92, 61.02, 52.30, 45.34, 38.66, 37.58, 37.19, 36.33, 33.57, 30.43, 25.54, 24.13, 19.56, 19.03, 18.88. HRMS (ESI): *m*/*z* calculated for C_27_H_37_ClN [M + H]^+^: 410.2614 and 412.2585. Found: 410.2611 and 412.2575. IR (film) *v*_max_: 2924, 2866, 1572, 1497, 1442, 1381, 1264, 1196, 1109 cm^−1^.

### 4.14. Synthesis of **18**

To a solution of dehydroabietylamine (156 mg, 55% purity, 0.3 mmol) in anhydrous acetonitrile (3 mL) was added potassium carbonate (124 mg, 0.9 mmol) followed by 1-bromobutane (96 μL, 0.9 mmol) at room temperature. The reaction was allowed to proceed at room temperature under argon overnight before diluting with EtOAc (75 mL). The resulting mixture was rinsed with brine (10 mL × 2), dried over anhydrous Na_2_SO_4_, and concentrated. The reaction mixture was stirred under argon at room temperature for six hours. The clear crude oil was subjected to PTLC purification, developing twice with hexane/EtOAc (5:1, *v*/*v*) to give the desired product as a colorless oil in 62% yield. ^1^H NMR (300 MHz, CDCl_3_) *δ* 7.19 (d, *J* = 8.1 Hz, 1H), 7.00 (dd, *J* = 8.1, 2.4 Hz, 1H), 6.90 (d, *J* = 2.4 Hz, 1H), 2.91–2.79 (m, 3H), 2.60 (d, *J* = 6.9 Hz, 2H), 2.52 (d, *J* = 11.7 Hz, 1H), 2.34 (d, *J* = 11.7 Hz, 1H), 2.28 (br.d, *J* = 12.7 Hz, 1H), 1.81–1.57 (m, 5H), 1.51–1.30 (m, 7H), 1.24 (d, *J* = 6.9 Hz, 6H, CH(*CH_3_)_2_*), 1.22 (s, 3H, *CH_3_*), 0.95 (s, 3H, *CH_3_*), 0.92 (t, *J* = 7.2 Hz, 3H, CH_2_*CH_3_*). ^13^C NMR (75 MHz, CDCl_3_) *δ* 147.77, 145.55, 135.00, 126.92, 124.46, 123.92, 61.99, 50.90, 45.68, 38.68, 37.59, 37.10, 36.40, 33.59, 32.41, 30.50, 25.51, 24.14, 20.64, 19.40, 19.05, 18.95, 14.18. HRMS (ESI): *m*/*z* calculated for C_24_H_40_N [M + H]^+^: 342.3161. Found: 342.3155. IR (film) *v*_max_: 2955, 2868, 1458, 1379, 1121, 974.8 cm^−1^.

### 4.15. Synthesis of **19**

To a solution of dehydroabietylamine (156 mg, 55% purity, 0.3 mmol) in DCM (3 mL) was added triethylamine (104 μL, 0.75 mmol) at 0 °C. The solution was stirred for 30 min before adding methyl chloroacetate (32 mg, 0.3 mmol). The reaction was allowed to proceed at room temperature under argon for 5 h before diluting with EtOAc (75 mL). The resulting mixture was rinsed with brine (10 mL × 2), dried over anhydrous Na_2_SO_4_, and concentrated. The crude oil was subjected to PTLC purification by developing twice with hexane/EtOAc (6:1, *v*/*v*) to give the desired product as a clear oil in 51% yield. ^1^H NMR (300 MHz, CDCl_3_) *δ* 7.19 (d, *J*= 8.3 Hz, 1H), 6.99 (dd, *J* = 8.3, 2.4 Hz, 1H), 6.90 (d, *J* = 2.4 Hz, 1H), 4.19 (q, *J* = 7.2 Hz, 2H, *CH_2_*CH_3_), 3.43 (d, *J* = 17.3 Hz, 1H), 3.33 (d, *J* = 17.3 Hz, 1H), 2.93–2.79 (m, 3H), 2.56 (d, *J* = 11.7 Hz, 1H), 2.28 (d, *J* = 11.5 Hz, 1H), 1.82–1.69 (m, 5H), 1.51–1.40 (m, 4H), 1.29 (t, *J* = 7.2 Hz, 3H), 1.24 (d, *J* = 6.9 Hz, 6H, CH(CH_3_)_2_), 1.23 (s, 3H, CH_3_), 0.94 (s, 3H, CH_3_). ^13^C NMR (75 MHz, CDCl_3_) *δ* 172.88, 147.54, 145.46, 134.84, 126.82, 124.30, 123.81, 61.44, 60.67, 52.07, 45.35, 38.55, 37.47, 37.15, 36.12, 33.50, 30.30, 25.35, 24.08, 19.18, 18.90, 18.83, 14.32. HRMS (ESI): *m*/*z* calculated for C_24_H_38_NO_2_ [M + H]^+^: 372.2902. Found: 372.2896. IR (film) *v*_max_: 2926,1736,1497,1192, 1036 cm^−1^.

### 4.16. Synthesis of **20**

To a solution of dehydroabietylamine (379 mg, 55% purity, 0.73 mmol) in DCM (3.5 mL) were added triethylamine (0.195 mL, 1.4 mmol) and 4-dimethylaminopyridine (21 mg, 0.17 mmol) under argon at 0 °C. A solution of di-*tert*-butyl dicarbonate (135 mg, 0.62 mmol) in DCM (3.8 mL) was then added to the reaction mixture. The reaction was allowed to proceed with stirring overnight at room temperature until the reaction turned pinkish and the reaction was complete as monitored by TLC (hexane/EtOAc, 10:1). The reaction mixture was diluted with DCM (30 mL), and the resulting mixture was rinsed with HCl solution (1 M, 10 mL) and saturated NaHCO_3_ solution (10 mL), respectively. The organic layer was dried over anhydrous Na_2_SO_4_ and concentrated. The crude product was purified by PTLC developing with hexane/EtOAC (10:1, *v*/*v*) to give the desired product as a colorless oil in 43% yield. ^1^H NMR (300 MHz, CDCl_3_) *δ* 7.18 (d, *J* = 8.1, 1H), 7.00 (dd, *J* = 8.1, 2.4 Hz, 1H), 6.90 (d, *J* = 2.4 Hz, 1H), 4.53 (br.s, 1H), 3.11 (dd, *J* = 13.8, 6.3 Hz, 1H), 2.97–2.78 (m, 5H), 2.28 (d, *J* = 12.9 Hz, 1H), 1.89–1.58 (m, 7H), 1.42 (s, 9H, C*(CH_3_)_3_*), 1.23 (d, *J* = 6.9 Hz, 6H, CH(CH_3_)_2_)), 1.22 (s, 3H, *CH_3_*), 0.91 (s, 3H, *CH_3_*). ^13^C NMR (75 MHz, CDCl_3_) *δ* 150.30, 147.39, 145.75, 134.97, 127.02, 124.40, 123.98, 79.22, 51.21, 44.99, 38.50, 37.56, 37.49, 36.12, 33.57, 30.38, 28.53, 25.45, 24.13, 24.08, 18.98, 18.76. HRMS (ESI): *m*/*z* calculated for C_25_H_40_NO_2_ [M + H]^+^: 386.3059. Found: 386.3051. IR (film) *v*_max_: 3350, 2959, 1694, 1389, 1165, 1039 cm^−1^.

### 4.17. Synthesis of **21**

To a solution of dehydroabietylamine (156 mg, 55% purity, 0.3 mmo) in DCM (3 mL) was added triethylamine (104 μL, 0.75 mmol) at 0 °C. The solution was stirred for 30 min before adding acetyl chloride (21 μL, 0.3 mmol). The reaction was allowed to proceed at 0 °C under argon for 1 h before adding 10 M HCl (0.03 mL, 0.3 mmol). The mixture was then diluted with EtOAc (75 mL). The resulting mixture was rinsed with brine (10 mL × 2), dried over anhydrous Na_2_SO_4_, and concentrated. The yellow crude oil was purified via PTLC, developing twice with hexane/EtOAc (2:1, *v*/*v*) to furnish the desired product as a clear oil in 43% yield. ^1^H NMR (300 MHz, CDCl_3_) *δ* 7.17 (d, *J* = 8.2 Hz, 1H), 6.99 (dd, *J* = 8.1, 2.4 Hz, 1H), 6.89 (d, *J* = 2.4 Hz, 1H), 5.87 (br.s, 1H), 3.24 (dd, *J* = 13.5, 6.3 Hz, 1H), 3.08 (dd, *J* = 13.8, 6.6 Hz, 1H), 2.96–2.75 (m, 3H), 2.29 (br.d, *J* = 14.4 Hz, 1H), 1.98 (s, 3H), 1.92–1.85 (m, 2H), 1.79–1.59 (m, 3H), 1.44–1.36 (m, 3H), 1.22 (d, *J* = 6.9 Hz, 6H), 1.21 (s, 3H), 0.94 (s, 3H). ^13^C NMR (75 MHz, CDCl_3_) *δ* 170.50, 147.23, 145.72, 134.85, 127.01, 124.21, 123.92, 50.00, 45.22, 38.39, 37.49, 37.39, 36.22, 33.49, 30.23, 25.35, 24.07, 24.03, 23.48, 23.41, 19.02, 18.81, 18.66. HRMS (ESI): *m*/*z* calculated for C_22_H_34_NO [M + H]^+^: 328.2640. Found: 328.2639. IR (film) *v*_max_: 2924, 2866, 1693, 1440, 1286, 1039 cm^−1^.

### 4.18. Synthesis of **22**

To a solution of dehydroabietylamine (156 mg, 55% purity, 0.3 mmo) in DCM (3 mL) was added triethylamine (104 μL, 0.75 mmol) at 0 °C. The solution was stirred for 30 min before adding dimethylcarbamoyl chloride (32 mg, 0.3 mmol). The reaction was allowed to proceed at room temperature under argon for 6 h before diluting with EtOAc (75 mL). The resulting mixture was rinsed with brine (10 mL × 2), dried over anhydrous Na_2_SO_4_, and concentrated. The clear crude oil was subjected to PTLC purification eluting with hexane/EtOAc (2:1, *v*/*v*) to give the desired product as a clear oil in 67% yield. ^1^H NMR (300 MHz, CDCl_3_) *δ* 7.16 (d, *J* = 8.4 Hz, 1H), 6.98 (d, *J* = 8.4 Hz, 1H), 6.88 (d, *J* = 2.4 Hz, 1H), 4.49 (br,s, 1H, NH), 3.20 (dd, *J* = 13.8, 5.7 Hz, 1H), 3.08 (dd, *J* = 13.8, 5.7 Hz, 1H), 2.87 (s, 6H), 2.27 (d, *J* = 14.4 Hz, 1H), 1.77–1.58 (m, 6H), 1.45–1.29 (m, 5H), 1.21 (d, *J* = 6.9 Hz, 6H), 1.20 (s, 3H), 0.91 (s, 3H). ^13^C NMR (75 MHz, CDCl_3_) *δ* 158.52, 147.34, 145.59, 135.03, 127.02, 124.31, 123.87, 60.45, 51.31, 45.43, 38.45, 37.57, 37.44, 36.42, 36.27, 33.48, 30.57, 25.56, 24.06, 24.03, 19.03, 18.75. HRMS (ESI): *m*/*z* calculated for C_23_H_37_N_2_O [M + H]^+^: 357.2906. Found: 357.2902. IR (film) *v*_max_: 3349, 1636, 1529, 1219, 908 cm^−1^.

### 4.19. Synthesis of **23**

To a solution of dehydroabietylamine (156 mg, 55% purity, 0.3 mmo) in DCM (3 mL) was added triethylamine (104 μL, 0.75 mmol) at 0 °C. The solution was stirred for 30 min before adding 2,4-dimethylbenzoyl chloride (51 mg, 0.3 mmol). The reaction was allowed to proceed at room temperature under argon for 4 h before being diluted with EtOAc (75 mL). The resulting mixture was rinsed with brine (10 mL × 2), dried over anhydrous Na_2_SO_4_, and concentrated. The light-yellow crude product was subjected to PTLC purification by developing three times with hexane/EtOAc (8:1, *v*/*v*) to give the desired product as a clear wax in 54% yield. ^1^H NMR (300 MHz, CDCl_3_) *δ* 7.33 (s, 2H), 7.17 (d, *J* = 8.4 Hz, 1H), 7.11 (s, 1H), 6.99 (dd, *J* = 8.1, 2.4 Hz, 1H), 6.89 (d, *J* = 2.4 Hz, 1H), 6.12 (t, *J* = 5.7 Hz, 1H, N*H*), 3.43 (dd, *J* = 13.8, 6.6 Hz, 1H), 3.32 (dd, *J* = 13.8, 6.6 Hz, 1H), 2.97–2.78 (m, 3H), 2.41–2.29 (m, 2H), 2.34 (s, 6H, 2 × CH_3_), 2.02–1.96 (m, 1H), 1.84–1.66 (m, 3H), 1.55–1.36 (m, 3H), 1.24 (s, 3H, CH_3_), 1.22 (d, *J* = 6.9 Hz, 6H, CH*(CH_3_)_2_)*, 1.01 (s, 3H, CH_3_). ^13^C NMR (75 MHz, CDCl_3_) *δ* 168.14, 147.19, 145.73, 138.38, 134.97, 133.11, 127.07, 124.72, 124.65, 124.33, 123.96, 60.52, 50.48, 38.46, 37.80, 37.67, 36.52, 33.53, 30.53, 25.57, 24.08, 21.34, 19.23, 18.86, 18.75, 14.32. HRMS (ESI): *m*/*z* calculated for C_29_H_40_NO [M + H]^+^: 418.3110. Found: 418.3109. IR (film) *v*_max_: 3292, 2916, 2865, 1685, 1497, 1245, 1038 cm^−1^.

### 4.20. Synthesis of **24**

To a solution of dehydroabietylamine (202 mg, 55% purity, 0.39 mmol) in DCM (4 mL) was added triethylamine (0.14 mL, 1.0 mmol) at 0 °C under argon, and the mixture was stirred for 15–20 min before adding dimethyl thiocarbonyl chloride (73 mg, 0.59 mmol). The reaction was then allowed to proceed with stirring at room temperature for two days prior to being diluted with ethyl acetate (50 mL). The resulting mixture was rinsed with brine (10 mL × 3), dried over anhydrous Na_2_SO_4,_ and concentrated. The obtained crude product was purified by PTLC developing twice with hexane/EtOAc (1:2, *v*/*v*) to give the desired product as a yellow oil in 61% yield. ^1^H NMR (300 MHz, CDCl_3_) *δ* 7.16 (d, *J* = 8.2 Hz, 1H), 6.99 (dd, *J* = 8.2, 2.1 Hz, 1H), 6.89 (d, *J* = 2.1 Hz, 1H), 5.49 (br.s, 1H), 3.72 (dd, *J* = 13.5, 5.1 Hz, 1H), 3.59 (dd, *J* = 13.2, 4.5 Hz, 1H), 3.25 (s, 6H, *N(CH_3_)_2_*), 2.91–2.77 (m, 3H), 2.30 (d, *J* = 13.5 Hz, 1H), 1.98 (dd, *J* = 13.5, 6.6 Hz, 1H), 1.82–1.65 (m, 3H), 1.51–1.29 (m, 4H), 1.22 (d, *J* = 6.9 Hz, 6H, CH*(CH_3_)_2_*), 1.22 (s, 3H, *CH_3_*), 0.99 (s, 3H, *CH_3_*). ^13^C NMR (75 MHz, CDCl_3_) *δ* 182.23, 147.04, 145.70, 134.83, 127.02, 124.24, 123.93, 56.86, 46.21, 40.58, 38.39, 37.67, 37.63, 36.82, 33.46, 30.45, 25.54, 24.05, 24.00, 19.24, 18.87, 18.67. HRMS (ESI): *m*/*z* calculated for C_23_H_37_N_2_S [M + H]^+^: 373.2677. Found: 373.2675. IR (film) *v*_max_: 3332, 2923,1733, 1533, 1408, 1125, 909 cm^−1^.

### 4.21. Cell Culture

The four prostate cancer cell lines (LNCaP, 22Rv1, DU145, and PC-3) were initially procured from the ATCC (American Type Culture Collection, Manassas, VA, USA). The three cell lines (PC-3, LNCaP, and 22Rv1) were cultured on a regular basis in RPMI-1640 medium, supplemented with 10% FBS and 1% penicillin/streptomycin. The cultures were sustained at 37 °C in a humid environment with 5% CO_2_ supplementation. Eagle’s Minimum Essential Medium (EMEM), supplemented with 10% FBS and 1% penicillin/streptomycin, was employed to regularly culture the DU145 cells.

### 4.22. WST-1 Cell Proliferation Assay

The PC-3, DU145, and LNCaP prostate cancer cells were placed in 96-well plates at a density of 3200 cells per well in 200 µL of culture medium. A density of 6400 22Rv1 cells per well was used for seeding in 96-well plates, with each well containing 200 µL of culture medium. Subsequently, the cells were treated separately with enzalutamide as a positive control, or tricyclic diterpenoids at varying doses for 72 h. The vehicle control group was treated with equal volumes of DMSO. The cell culture was incubated at 37 °C in a CO_2_ incubator throughout this period. For cell proliferation assessment, 10 µL of the premixed WST-1 cell proliferation reagent (Clontech, Mountain View, CA, USA) was added to each well. After gently mixing on an orbital shaker for 1 min to ensure even color distribution, the cells were further incubated at 37 °C for 3 h. A microplate reader (Synergy HT, BioTek, Winooski, VT, USA) was utilized to measure the absorbance of each well at a wavelength of 430 nm. The IC_50_ value represented the concentration of each test compound that suppresses cell proliferation by 50% under the experimental conditions, which was determined by averaging triplicate determinations that were both reproducible and statistically significant. To calculate the IC_50_ values, a linear or logarithmic proliferative suppression curve was generated based on at least five dosages for each test compound.

### 4.23. Statistical Analysis

The mean ± SD (standard derivation) was used to represent all the data gathered from the indicated number of experiments. The differences between the treatment and control groups were analyzed using the student’s t-test, with statistical significance defined as a *p*-value < 0.05. 

## Figures and Tables

**Table 1:** Antiproliferative activities of tricyclic diterpenoids against prostate cancer cell lines.

Compound	IC_50_ (μM) ^a^			
	DU145 ^b^	PC-3 ^b^	LNCaP ^c^	22Rv1 ^c^
Enzalutamide	72.03 ± 3.07	>100	21.75 ± 4.37	67.54 ± 1.41
QW07 (**4**)	0.54 ^d^ 12.0–24.2 ^e^	0.50 ^d^ 12.0–24.2 ^e^	1.94–5.10 ^e^	1.94–5.10 ^e^
**5**	>100	>100	67.64 ± 16.41	21.44 ± 4.15
Abietic acid	>100	>100	43.10 ± 7.28	>100
**7**	28.68 ± 0.72	40.22 ± 5.45	19.30 ± 4.64	33.34 ± 2.18
**8**	69.58 ± 2.81	87.25 ± 3.89	64.08 ± 8.16	70.67 ± 3.05
**9**	>100	>100	75.37 ± 5.88	38.74 ± 5.17
**10**	22.47 ± 1.66	55.13 ± 3.76	19.57 ± 1.09	17.51 ± 2.19
**11**	47.03 ± 6.18	40.28 ± 3.81	33.61 ± 2.48	30.89 ± 2.48
**12**	>100	>100	>100	>100
**13**	62.59 ± 8.91	>100	39.85 ± 1.56	78.17 ± 8.34
**14**	>100	22.90 ± 6.76	1.99 ± 1.27	24.15 ± 10.95
**15**	>100	>100	>100	42.81 ± 4.10
**16**	21.41 ± 5.33	37.20 ± 2.59	20.35 ± 2.98	12.62 ± 4.80
**17**	>100	>100	55.45 ± 9.16	59.92 ± 0.41
**18**	6.63 ± 1.20	24.00 ± 6.95	2.38 ± 0.82	0.27 ± 0.18
**19**	>100	64.60 ± 2.65	17.83 ± 7.27	35.00 ± 9.70
**20**	35.80 ± 5.47	>100	6.95 ± 1.61	80.72 ± 10.22
**21**	50.27 ± 2.63	57.68 ± 2.95	23.52 ± 2.99	16.92 ± 6.27
**22**	56.43 ± 3.02	54.08 ± 1.64	43.37 ± 2.90	31.94 ± 1.97
**23**	>100	>100	>100	>100
**24**	6.32 ± 2.82	24.66 ± 2.50	1.66 ± 0.65	11.44 ± 1.78

^a^: IC_50_ is the half-maximal inhibitory concentration measured via WST-1 cell proliferation assay. The data were presented as mean ± standard deviation. ^b^: Human AR-negative prostate cancer cell line. ^c^: Human AR-positive prostate cancer cell line. ^d^: Reported in reference [16]. SRB assay after 96 h of treatment. ^e^: Reported in reference [15]. SRB assay after 48 h of treatment.

## Data Availability

All critical data have been included in the Supplementary Materials.

## Supplementary Materials

The following supporting information can be downloaded at: https://www.mdpi.com/article/10.3390/molecules28124743/s1, Figures S1, S2, S4, S5, S7, S8, S10, S11, S13, S14, S16, S17, S19, S20, S22, S23, S25, S26, S28, S29, S31, S32, S34, S35, S37, S38, S40, S41, S43, S44, S46, S47, S49, S50, S52, S53, S55, S56, S58, S59: NMR spectra; Figures S3, S6, S9, S12, S15, S18, S21, S24, S27, S30, S33, S36, S39, S42, S45, S48, S51, S54, S57, S60: High-resolution mass spectra.

## Sample Availability

Samples of compounds **5**–**24** are available from the authors.
