# Supplementary material for: Tricyclic Diterpenoids Selectively Suppress Androgen Receptor-Positive Prostate Cancer Cells
Source: Molecules. 2023 Jun 13;28(12):4743. doi: 10.3390/molecules28124743 (PMC10300971; doi:10.3390/molecules28124743)

Electronic Supplementary Information

Tricyclic Diterpenoids Selectively Suppress Androgen Receptor-Positive Prostate Cancer Cells

Inderpal Sekhon [1], Guanglin Chen [1], Keyara Piri [1], Seiji Shinkawa [1], Dennis Ashong [1], Qiang Zhang [2], Guangdi Wang [2] and Qiao-Hong Chen [1,*]

1 Department of Chemistry & Biochemistry, California State University, Fresno, CA 93740, USA; inderpal@mail.fresnostate.edu (I.S.); chen.bmc@gmail.com (G.C.); piri_keyara@mail.fresnostate.edu (K.P.); sashinkawa8@mail.fresnostate.edu (S.S.); dashong@mail.fresnostate.edu (D.A.)

2 Department of Chemistry and RCMI Cancer Research Center, Xavier University of Louisiana, New Orleans, LA 70125, USA; qzhang1@xula.edu (Q.Z.); gwang@xula.edu (G.W.)

* Correspondence: qchen@csufresno.edu; Tel: +1-559-278-2394

List of Contents:

Figure S1: ^{1}H NMR spectrum of **5** in $CDCl_3$……………………………………………………………..S4
Figure S2: ^{13}C NMR spectrum of **5** in $CDCl_3$…………………………………………………………..S5
Figure S3: High resolution mass spectrum of **5**……………………………………………………….. ..S6
Figure S4: ^{1}H NMR spectrum of **6** in $CDCl_3$…………………………………………………………..…S7
Figure S5: ^{13}C NMR spectrum of **6** in $CDCl_3$……………………………………………………….. …S8
Figure S6: High resolution mass spectrum of **6**………………………………………………………....S9
Figure S7: ^{1}H NMR spectrum of **7** in $CDCl_3$……………………………………………………………..S10
Figure S8: ^{13}C NMR spectrum of **7** in $CDCl_3$…………………...………………………………………..S11
Figure S9: High resolution mass spectrum of **7**……………..……………………………………..S12
Figure S10: ^{1}H NMR spectrum of **8** in $CDCl_3$……………………………………………………………S13
Figure S11: ^{13}C NMR spectrum of **8** in $CDCl_3$…………………………………………………………S14
Figure S12: High resolution mass spectrum of **8**……………………………………………………...S15
Figure S13: ^{1}H NMR spectrum of **9** in $CDCl_3$……………………………………………………………S16
Figure S14: ^{13}C NMR spectrum of **9** in $CDCl_3$…………………………………………………………S17
Figure S15: High resolution mass spectrum of **9**……………………………………………………...S18
Figure S16: ^{1}H NMR spectrum of **10** in $CDCl_3$………………………………………………………...S19
Figure S17: ^{13}C NMR spectrum of **10** in $CDCl_3$……………………………………………………….S20
Figure S18: High resolution mass spectrum of **10**…………………………………………………….S21
Figure S19: ^{1}H NMR spectrum of **11** in $CDCl_3$………………………………………………………...S22
Figure S20: ^{13}C NMR spectrum of **11** in $CDCl_3$……………………………………………………….S23

Figure S21: High resolution mass spectrum of **11**……S24
Figure S22: ^{1}H NMR spectrum of **12** in $CDCl_3$……S25
Figure S23: ^{13}C NMR spectrum of **12** in $CDCl_3$……S26
Figure S24: High resolution mass spectrum of **12**……S27
Figure S25: ^{1}H NMR spectrum of **13** in $CDCl_3$……S28
Figure S26: ^{13}C NMR spectrum of **13** in $CDCl_3$……S29
Figure S27: High resolution mass spectrum of **13**……S30
Figure S28: ^{1}H NMR spectrum of **14** in $CDCl_3$……S31
Figure S29: ^{13}C NMR spectrum of **14** in $CDCl_3$……S32
Figure S30: High resolution mass spectrum of **14**……S33
Figure S31: ^{1}H NMR spectrum of **15** in $CDCl_3$……S34
Figure S32: ^{13}C NMR spectrum of **15** in $CDCl_3$……S35
Figure S33: High resolution mass spectrum of **15**……S36
Figure S34: ^{1}H NMR spectrum of **16** in $CDCl_3$……S37
Figure S35: ^{13}C NMR spectrum of **16** in $CDCl_3$……S38
Figure S36: High resolution mass spectrum of **16**……S39
Figure S37: ^{1}H NMR spectrum of **17** in $CDCl_3$……S40
Figure S38: ^{13}C NMR spectrum of **17** in $CDCl_3$……S41
Figure S39: High resolution mass spectrum of **17**……S42
Figure S40: ^{1}H NMR spectrum of **18** in $CDCl_3$……S43
Figure S41: ^{13}C NMR spectrum of **18** in $CDCl_3$……S44
Figure S42: High resolution mass spectrum of **18**……S45
Figure S43: ^{1}H NMR spectrum of **19** in $CDCl_3$……S46
Figure S44: ^{13}C NMR spectrum of **19** in $CDCl_3$……S47
Figure S45: High resolution mass spectrum of **19**……S48
Figure S46: ^{1}H NMR spectrum of **20** in $CDCl_3$……S49
Figure S47: ^{13}C NMR spectrum of **20** in $CDCl_3$……S50
Figure S48: High resolution mass spectrum of **20**……S51
Figure S49: ^{1}H NMR spectrum of **21** in $CDCl_3$……S52
Figure S50: ^{13}C NMR spectrum of **21** in $CDCl_3$……S53
Figure S51: High resolution mass spectrum of **21**……S54
Figure S52: ^{1}H NMR spectrum of **22** in $CDCl_3$……S55
Figure S53: ^{13}C NMR spectrum of **22** in $CDCl_3$……S56
Figure S54: High resolution mass spectrum of **22**……S57
Figure S55: ^{1}H NMR spectrum of **23** in $CDCl_3$……S58
Figure S56: ^{13}C NMR spectrum of **23** in $CDCl_3$……S59

Figure S57: High resolution mass spectrum of **23**……………………………………………………..……….S60
Figure S58: ^{1}H NMR spectrum of **24** in $CDCl_3$……………………………………………………..……...S61
Figure S59: ^{13}C NMR spectrum of **24** in $CDCl_3$…………………………………………………..……..S62
Figure S60: High resolution mass spectrum of **24**…………………………………………………….….……S63

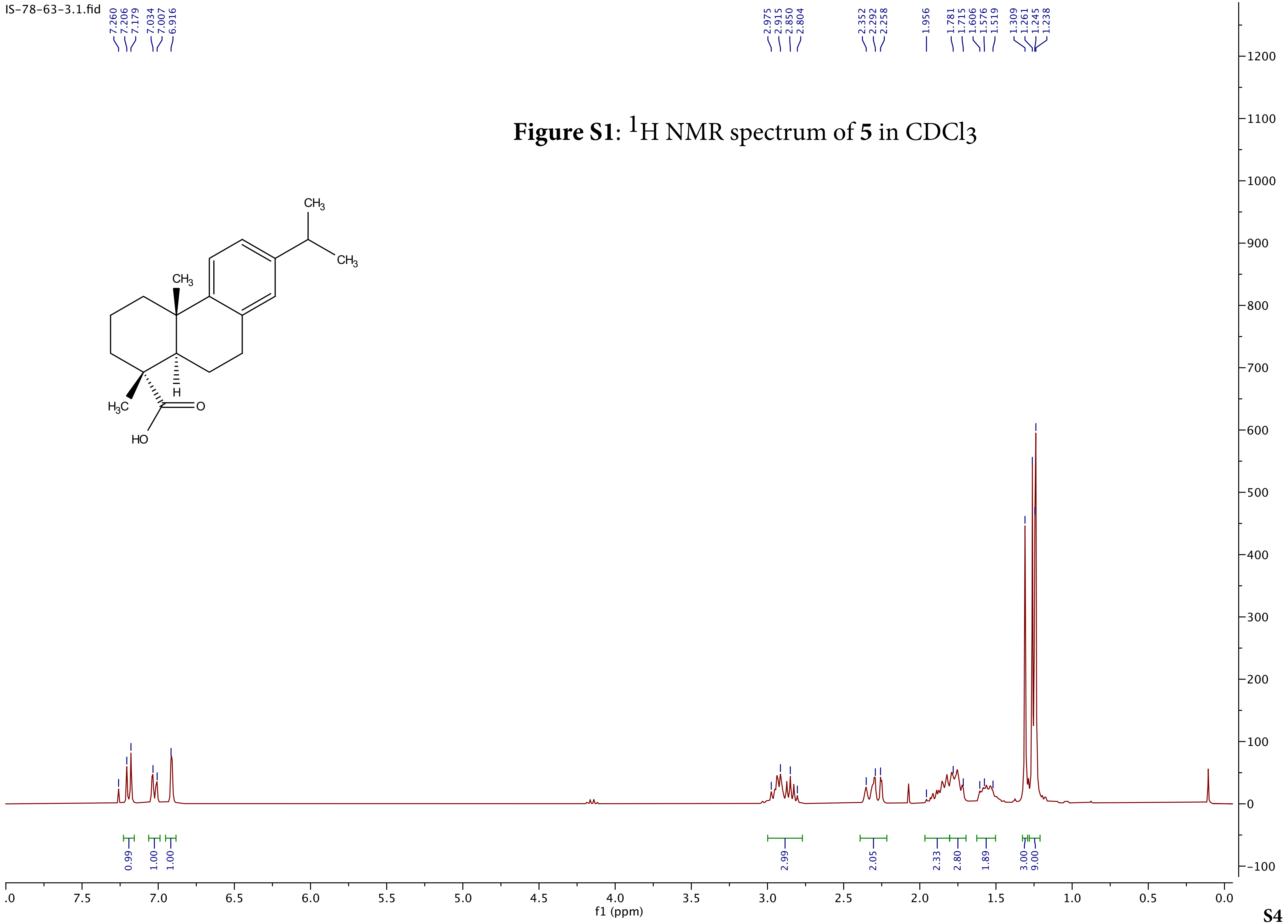

Figure S1: ^{1}H NMR spectrum of **5** in $CDCl_3$

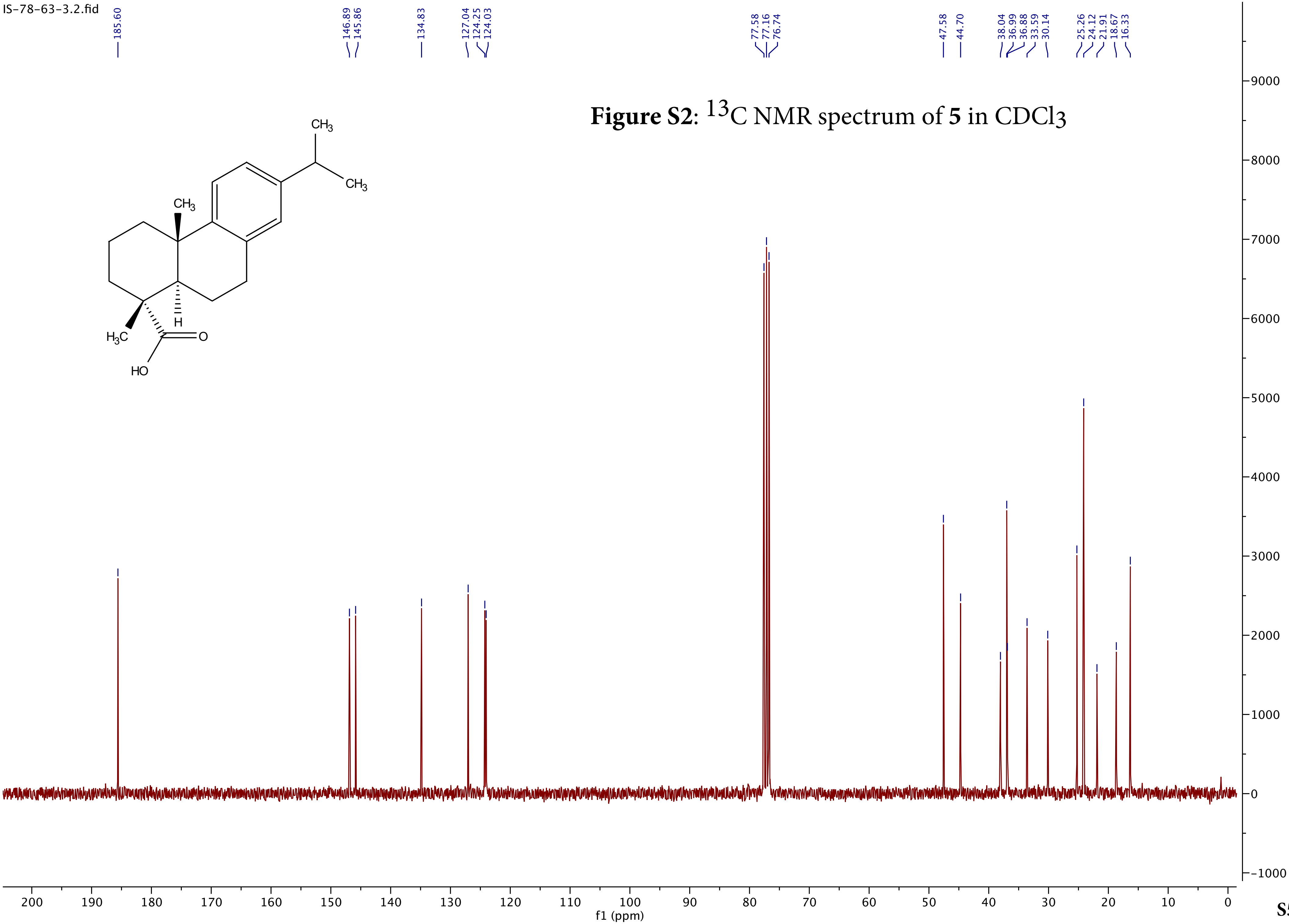

Figure S2: ^{13}C NMR spectrum of **5** in $CDCl_3$

Figure S3: High resolution mass spectrum of **5**

Sample Name	Mol Fomula	MW	M+H	observed	delta	ppm
IS-78-63-3	C20H28O2	300.2089	301.2167	301.2164	-0.0003	-1.13

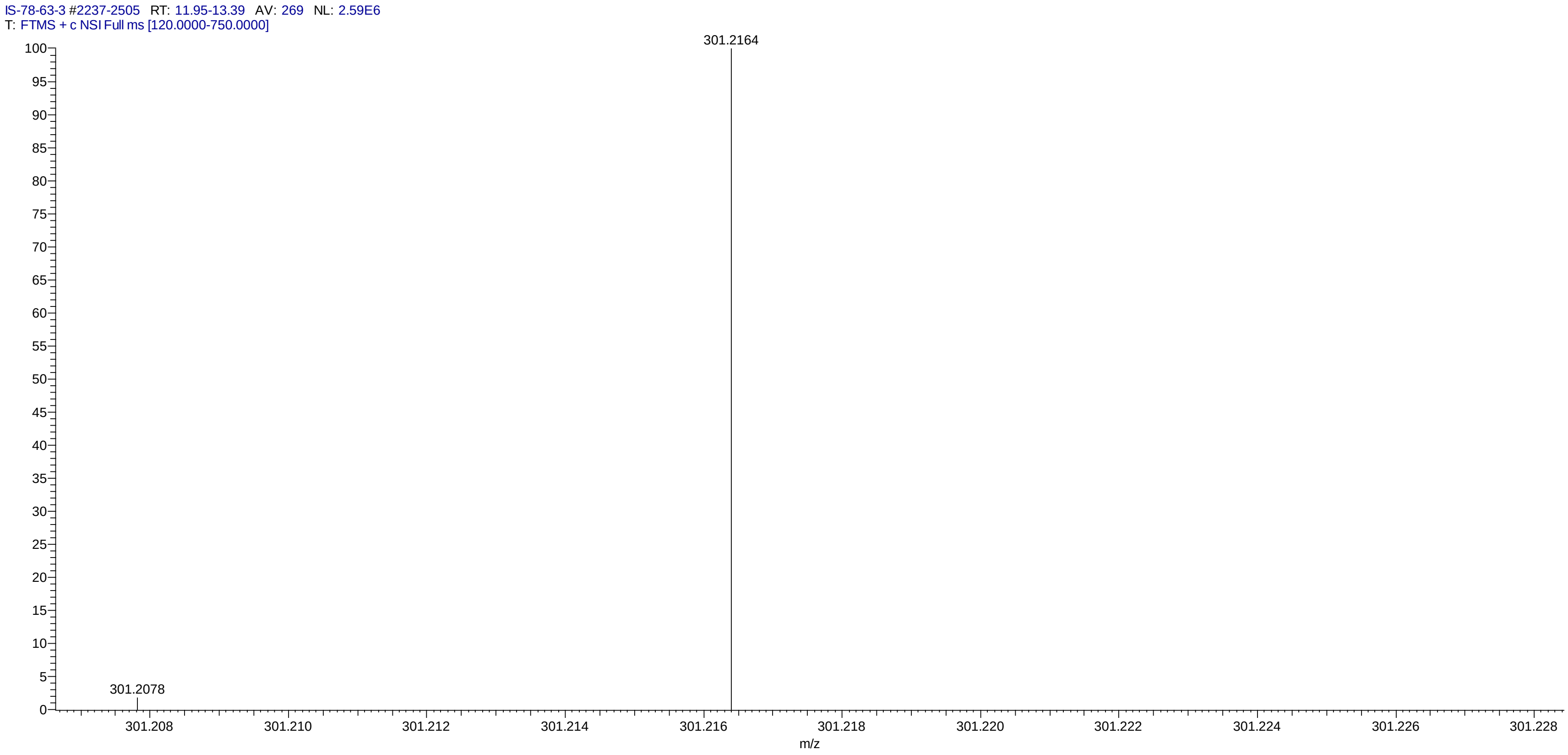

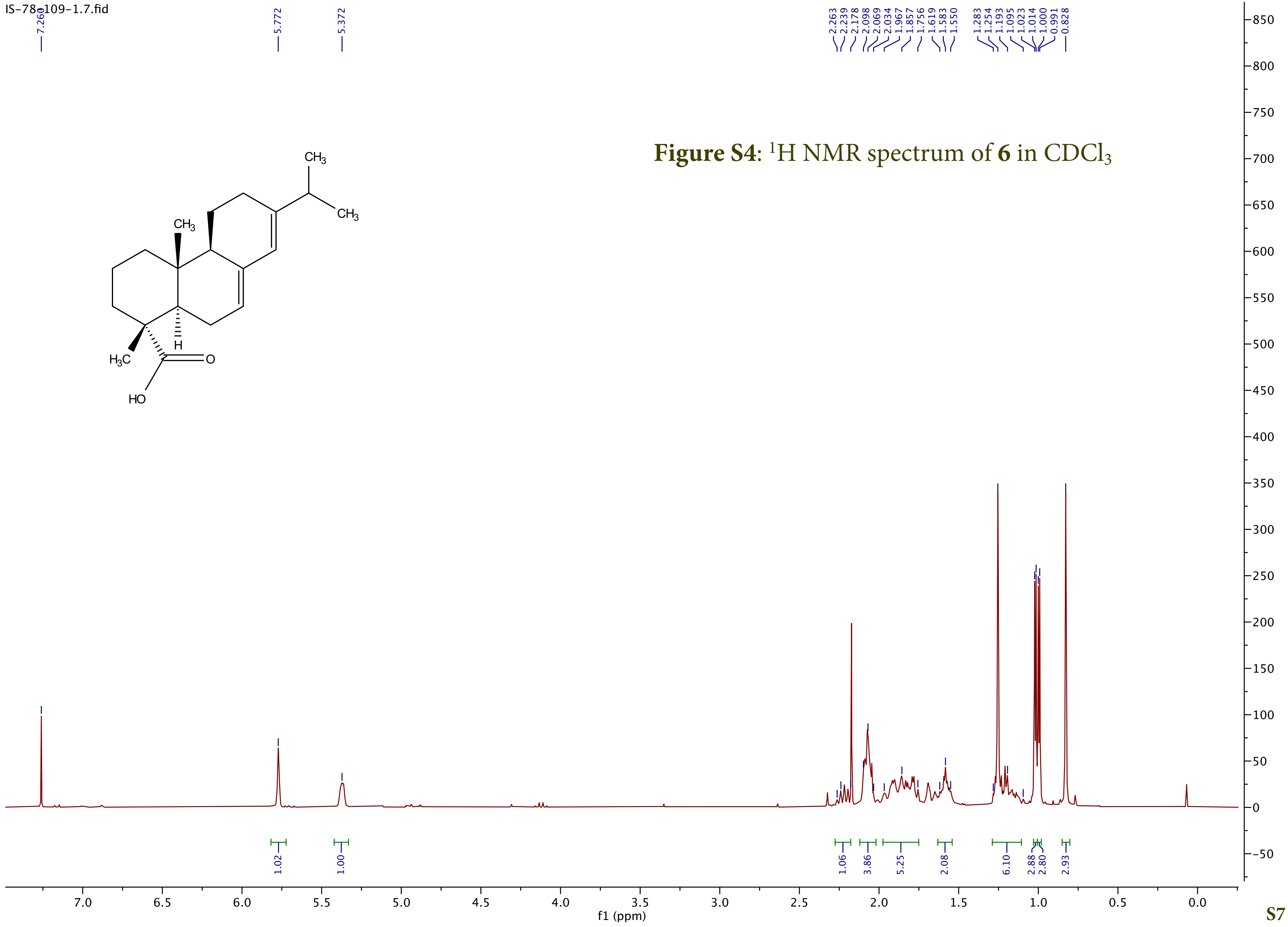

Figure S4: ^{1}H NMR spectrum of **6** in $CDCl_3$

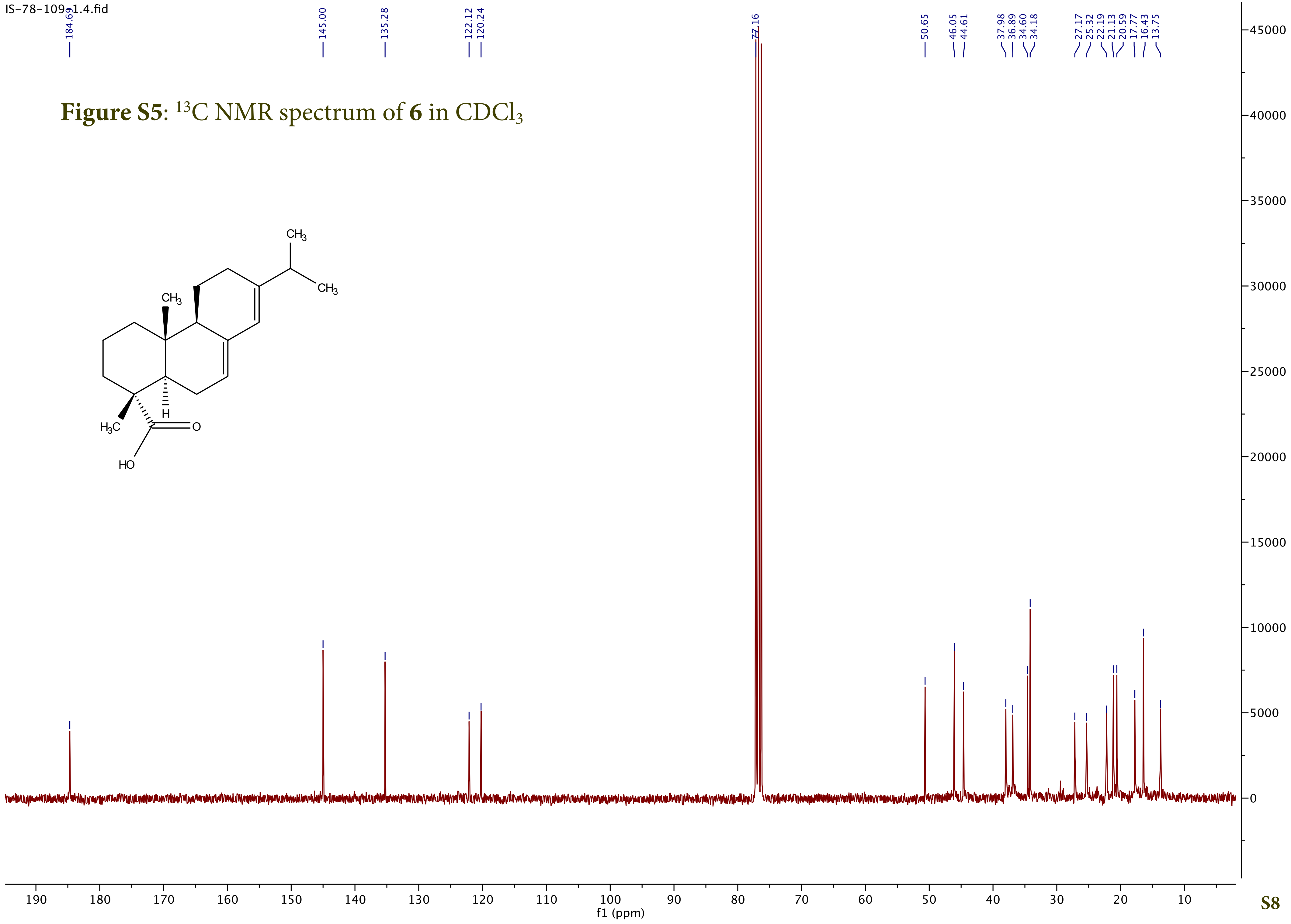

Figure S5: ^{13}C NMR spectrum of **6** in $CDCl_3$

Figure S6: High resolution mass spectrum of **6**

Sample Name	Mol Fomula	MW	M+H	observed	delta	ppm
IS-78-109-2	C20H30O2	302.2246	303.2324	303.2321	-0.0003	-0.96

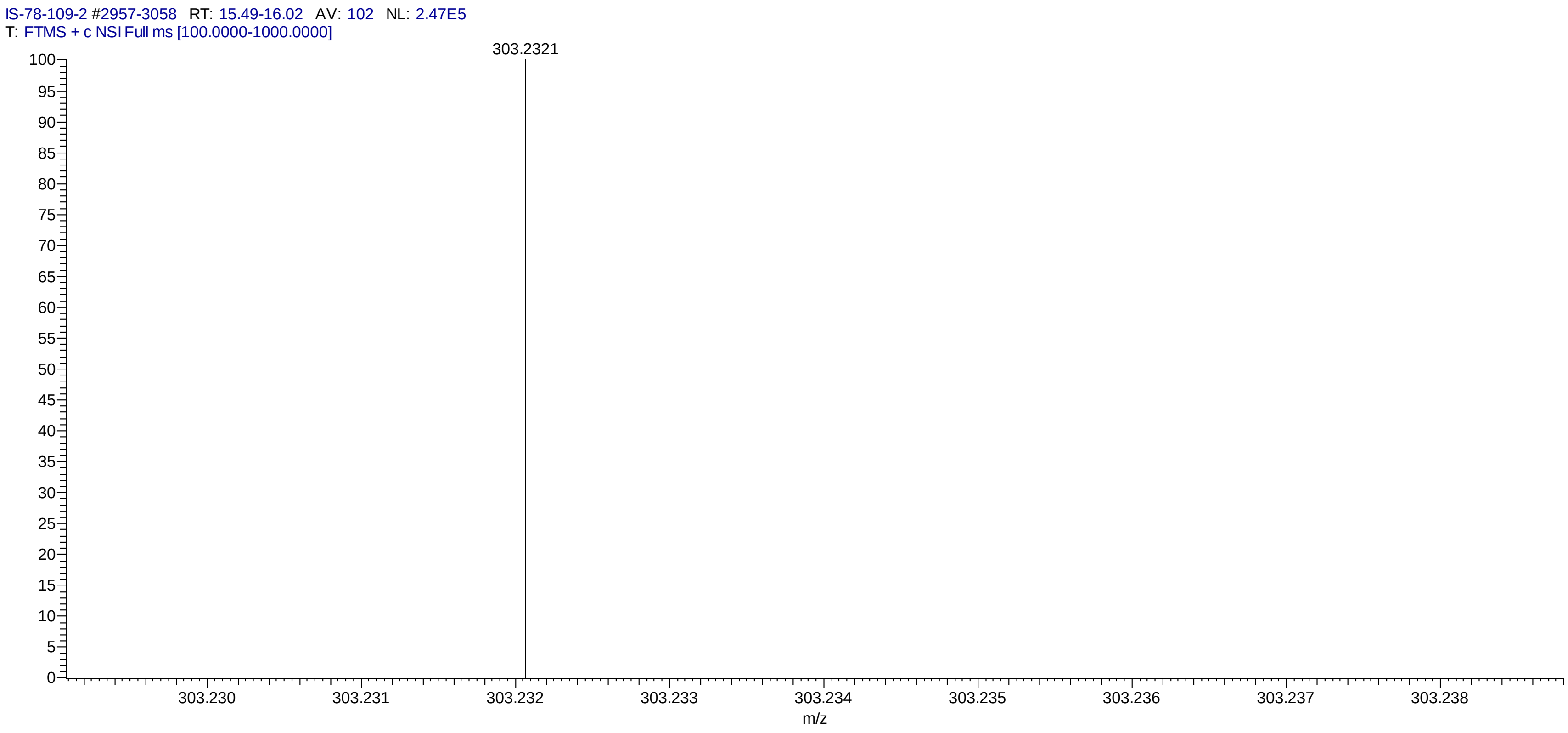

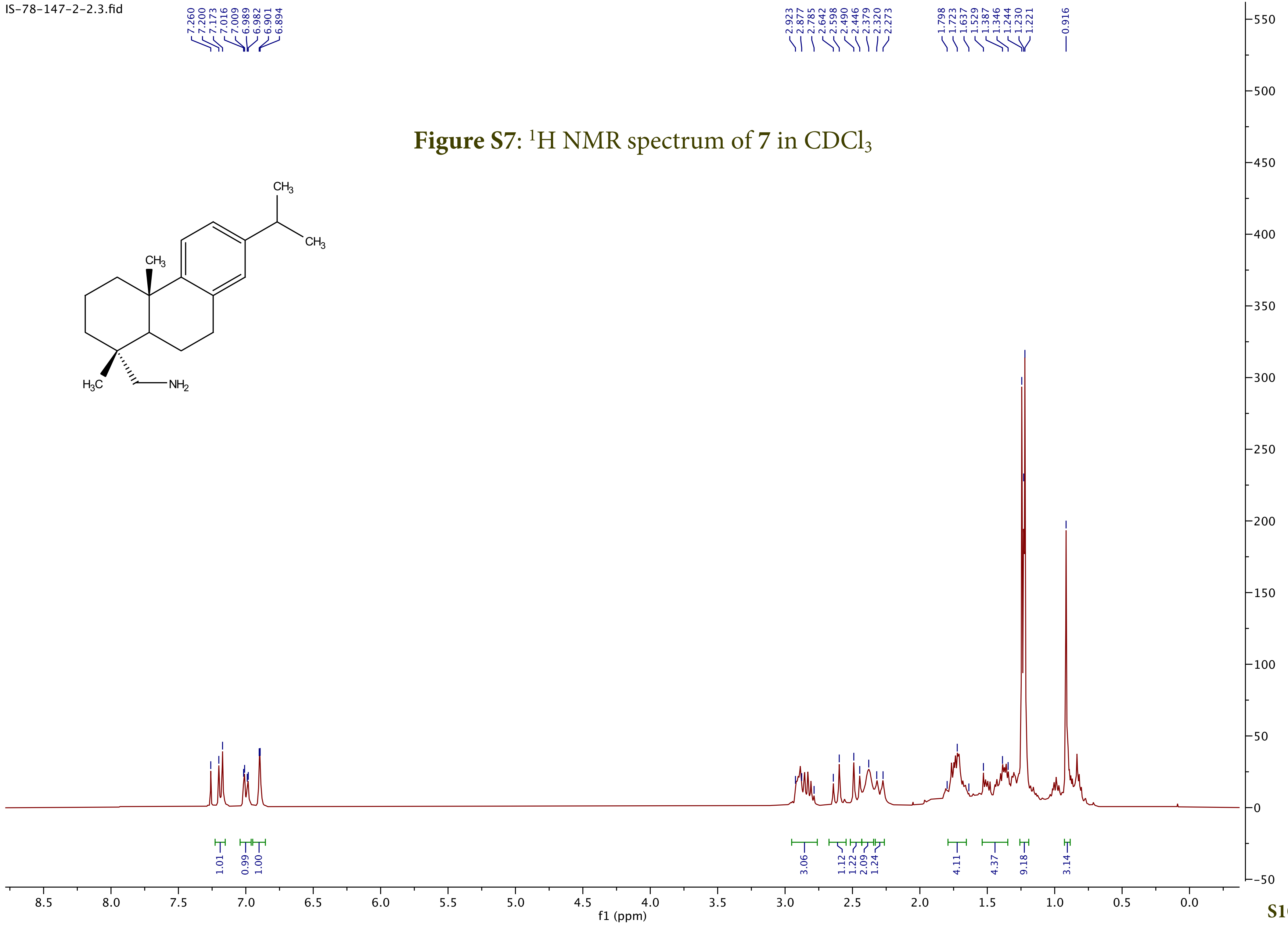

Figure S7: ^{1}H NMR spectrum of **7** in $CDCl_3$

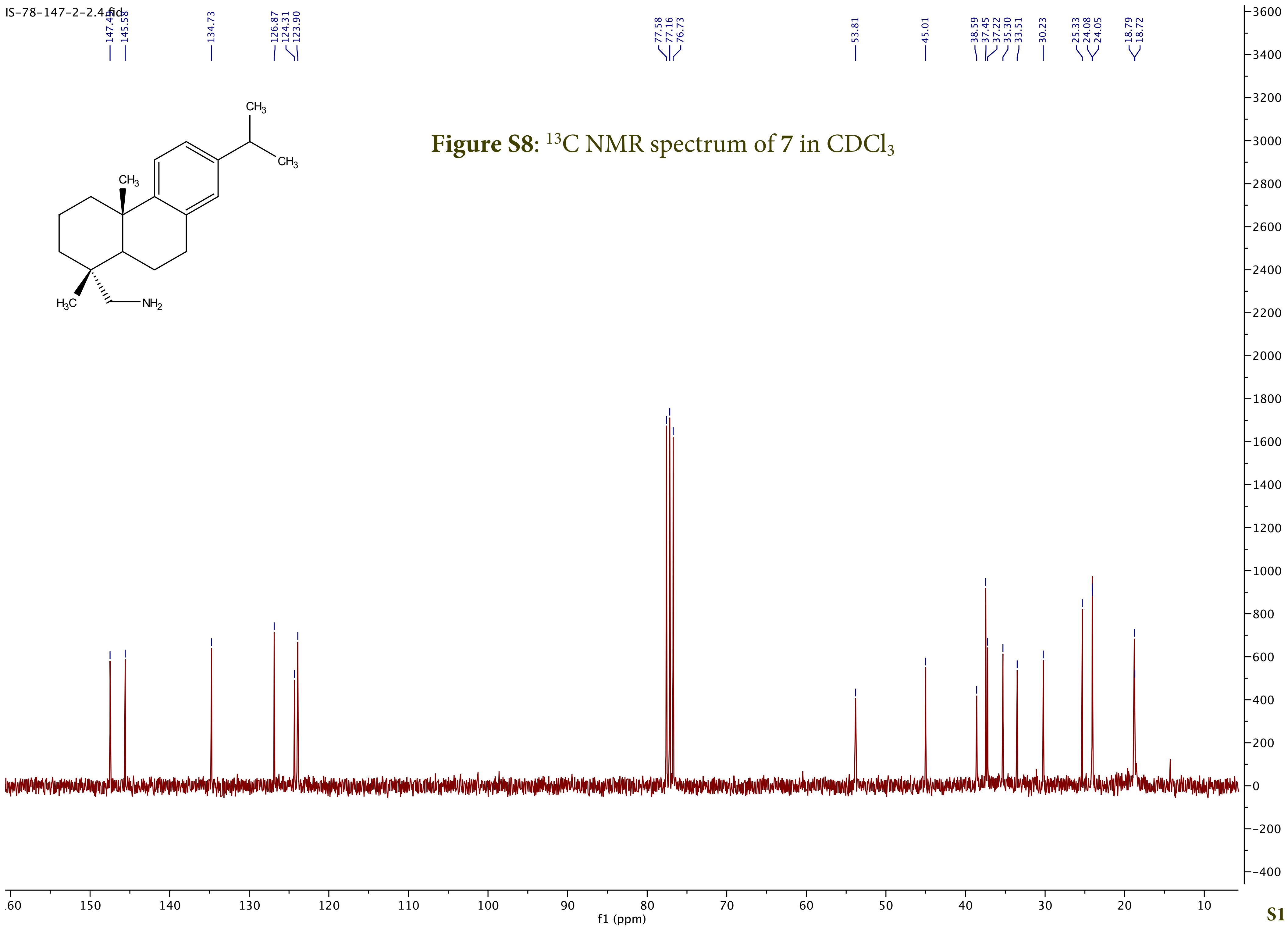

Figure S8: ^{13}C NMR spectrum of **7** in $CDCl_3$

Figure S9: High resolution mass spectrum of **7**

Sample Name	Mol Fomula	MW	M+H	observed	delta	ppm
IS-78-147-3	C20H31N	285.2457	286.2535	286.2530	-0.0005	-1.57

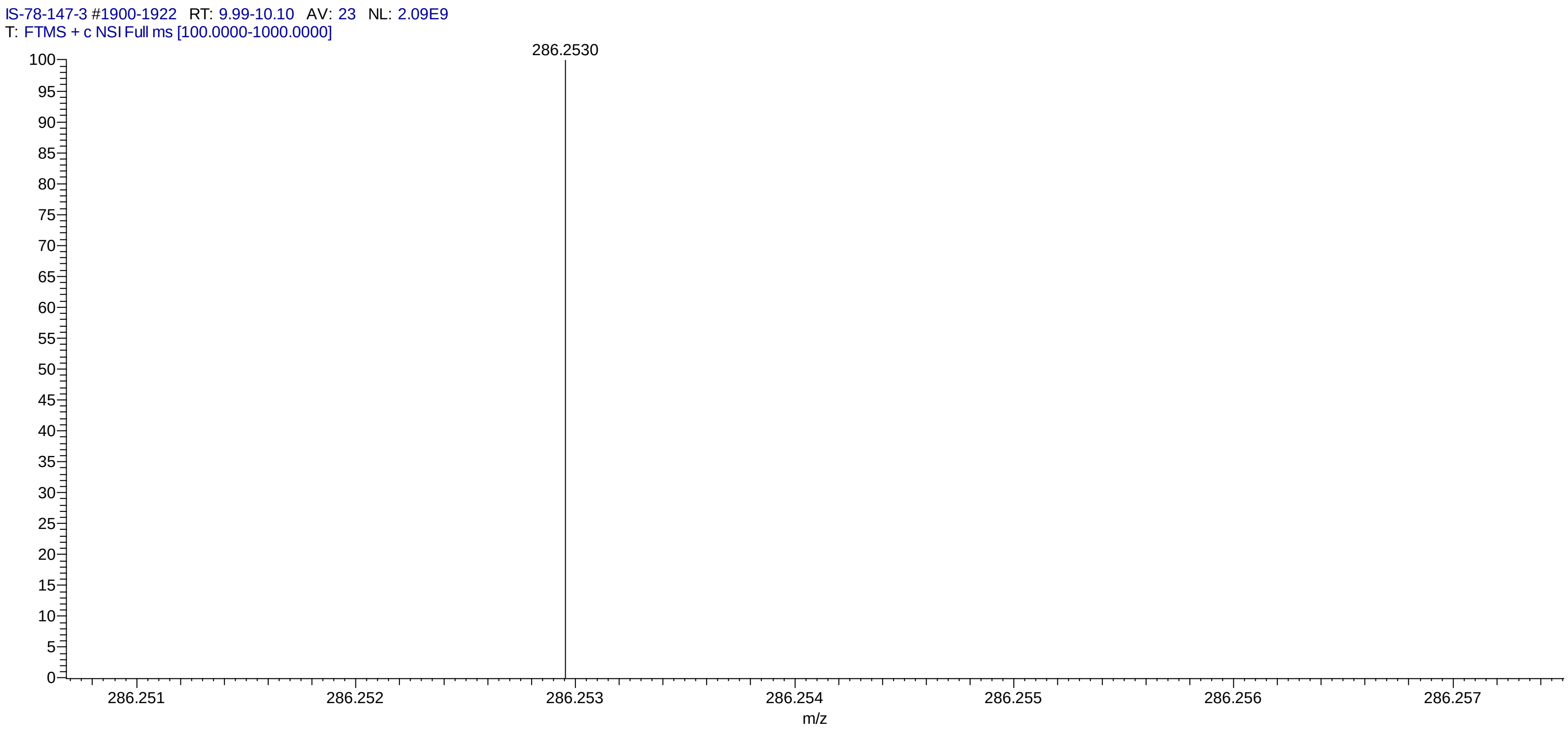

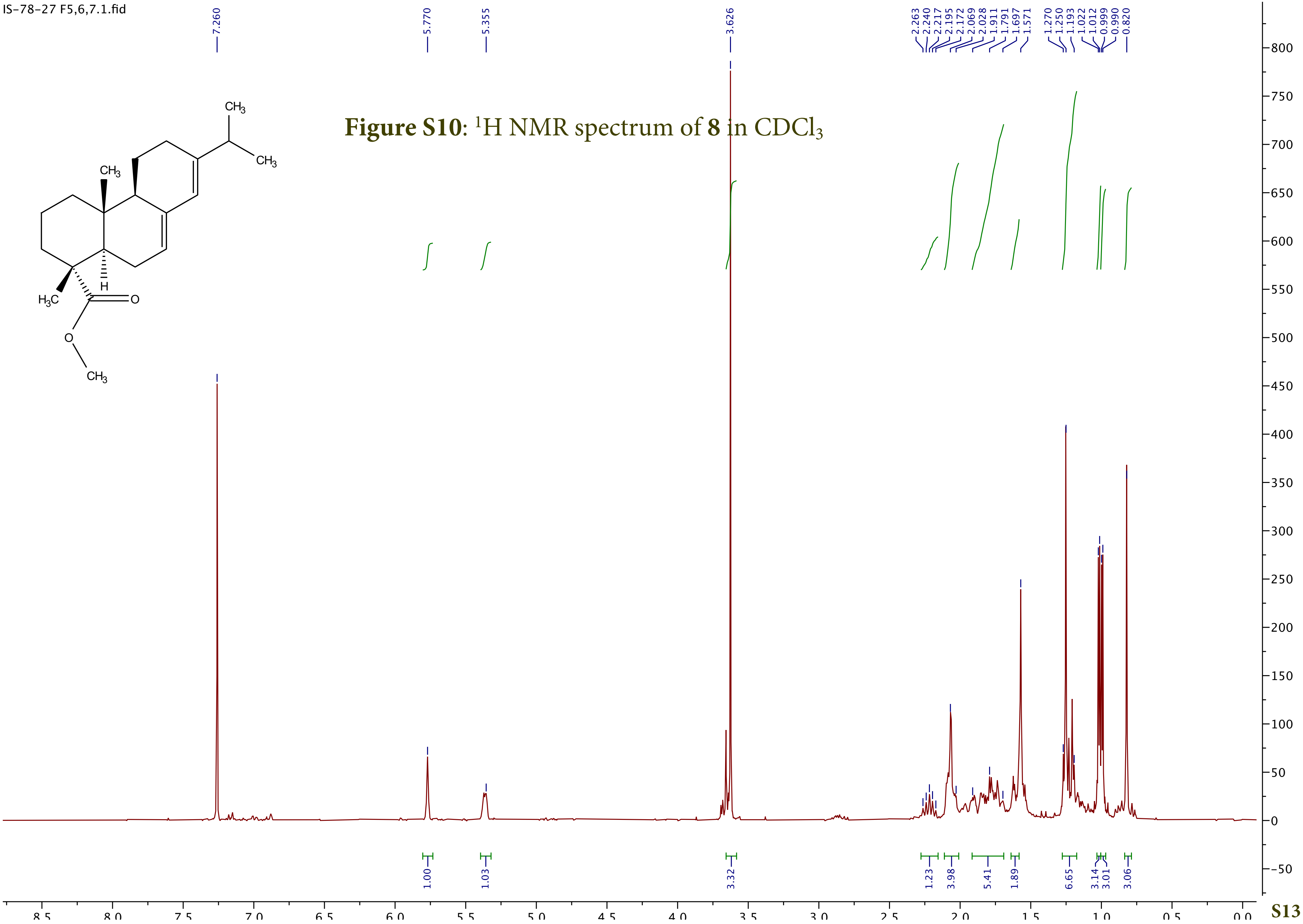

Figure S10: ^{1}H NMR spectrum of **8** in $CDCl_3$

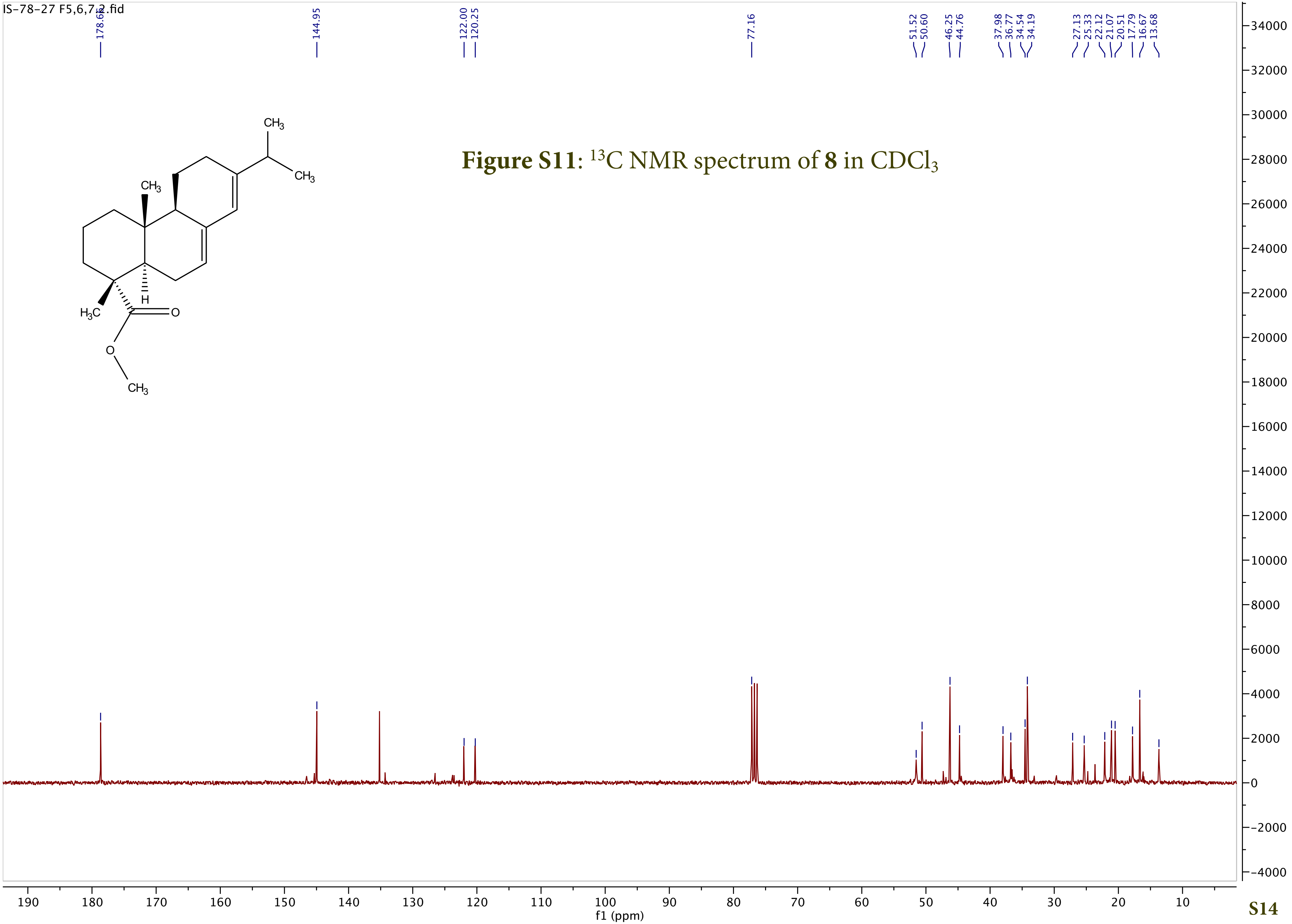

Figure S11: ^{13}C NMR spectrum of **8** in $CDCl_3$

Figure S12: High resolution mass spectrum of **8**

Sample Name	Mol Fomula	MW	M+H	observed	delta	ppm
IS-78-27-5	C21H32O2	316.2402	317.2480	317.2478	-0.0002	-0.76

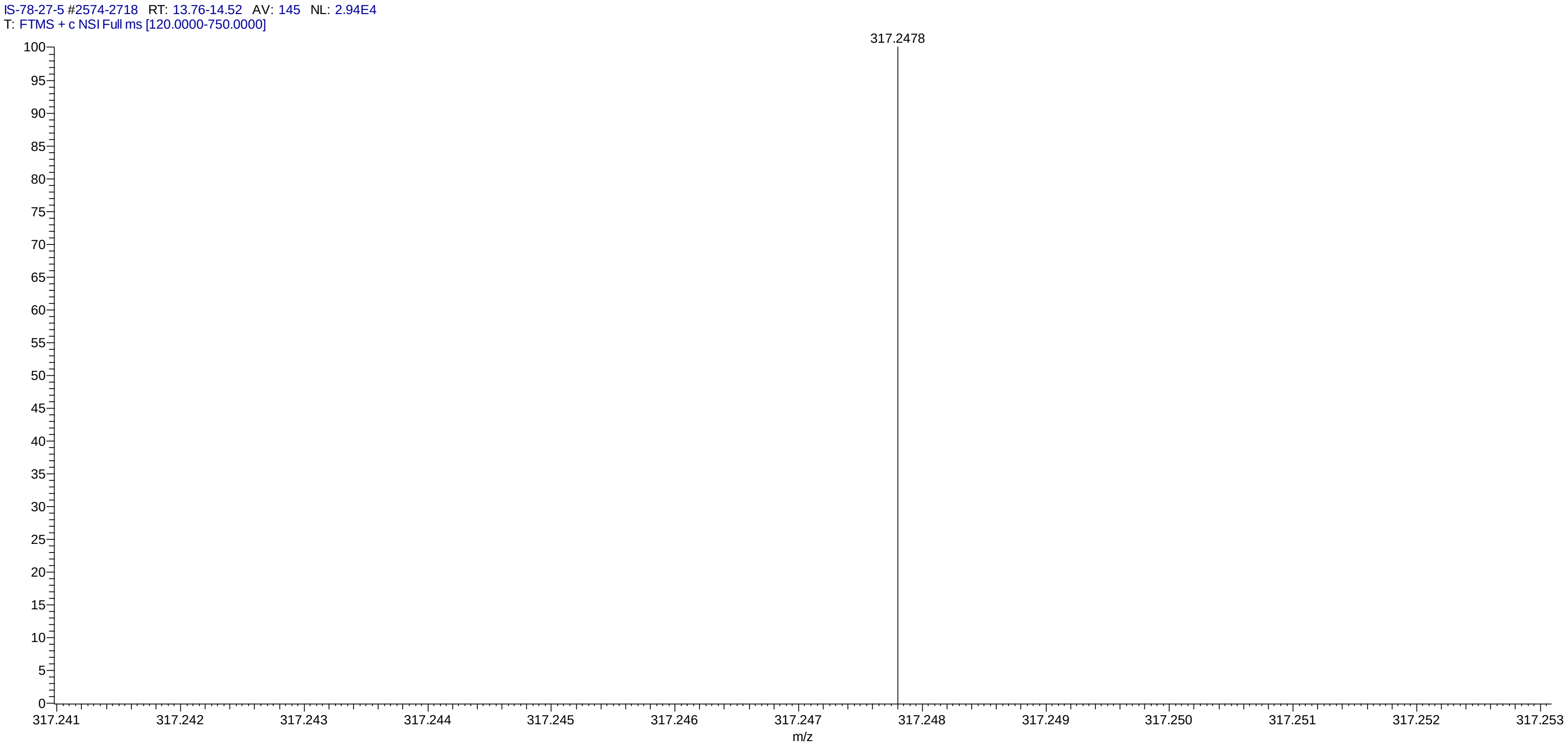

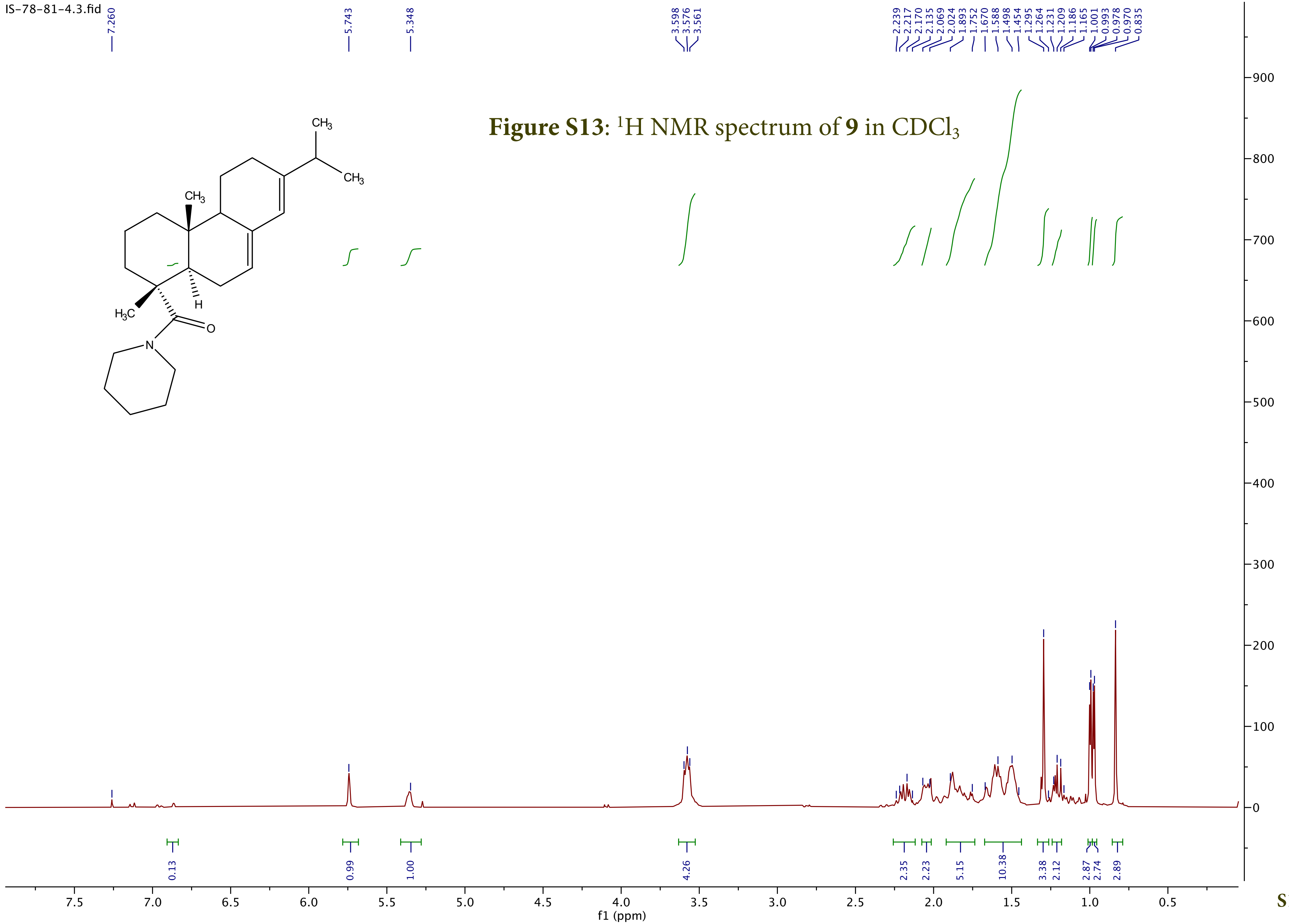

Figure S13: ^{1}H NMR spectrum of **9** in $CDCl_3$

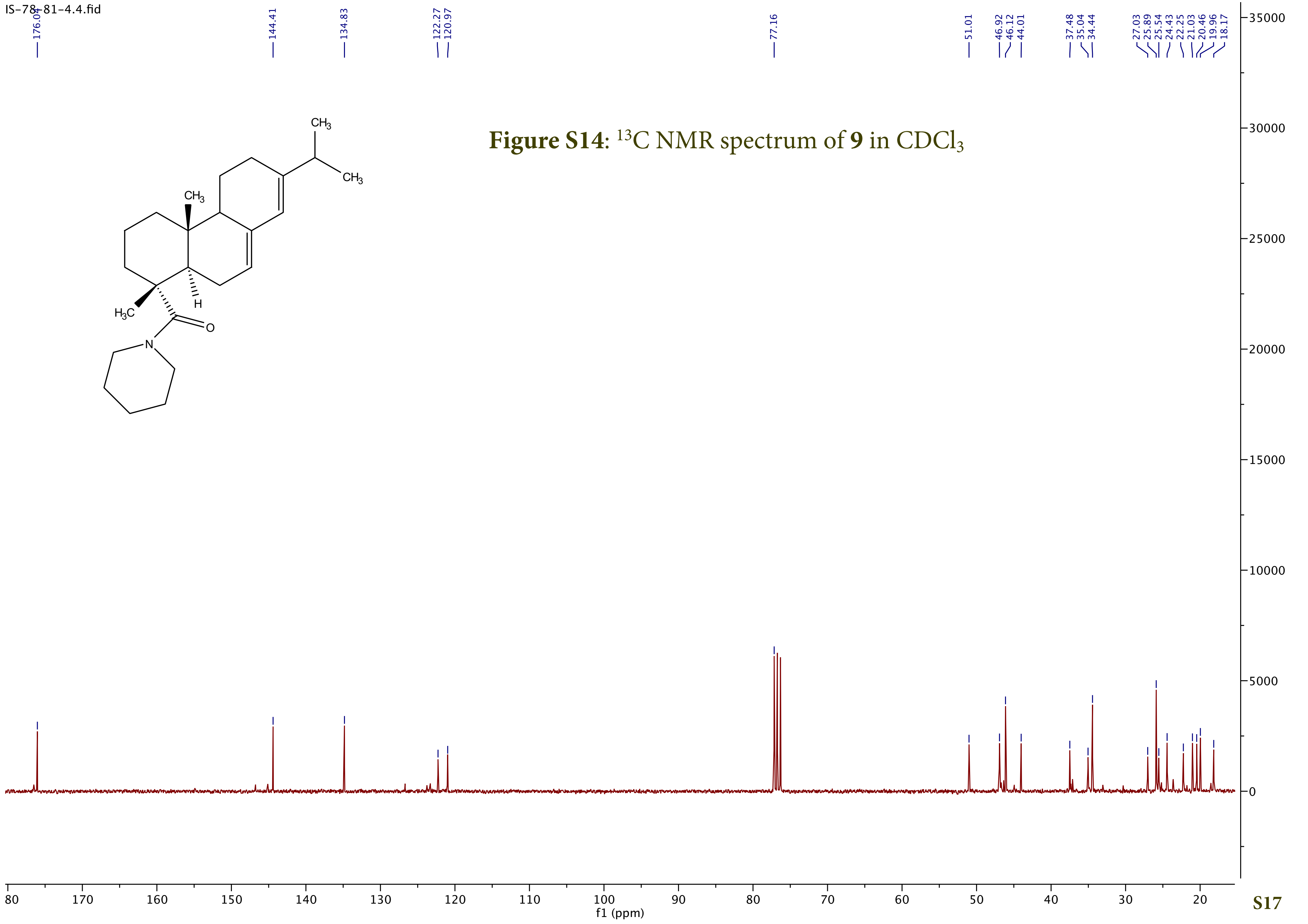

Figure S14: ^{13}C NMR spectrum of **9** in $CDCl_3$

Figure S15: High resolution mass spectrum of **9**

Sample Name	Mol Fomula	MW(COME WITH SAMPLES)	MW(CALCULATED)	M+H	observed	delta	ppm
**IS-78-81**	C25H39NO	369.3032	369.30317	370.3110	**370.3106**	-0.0004	-1.00

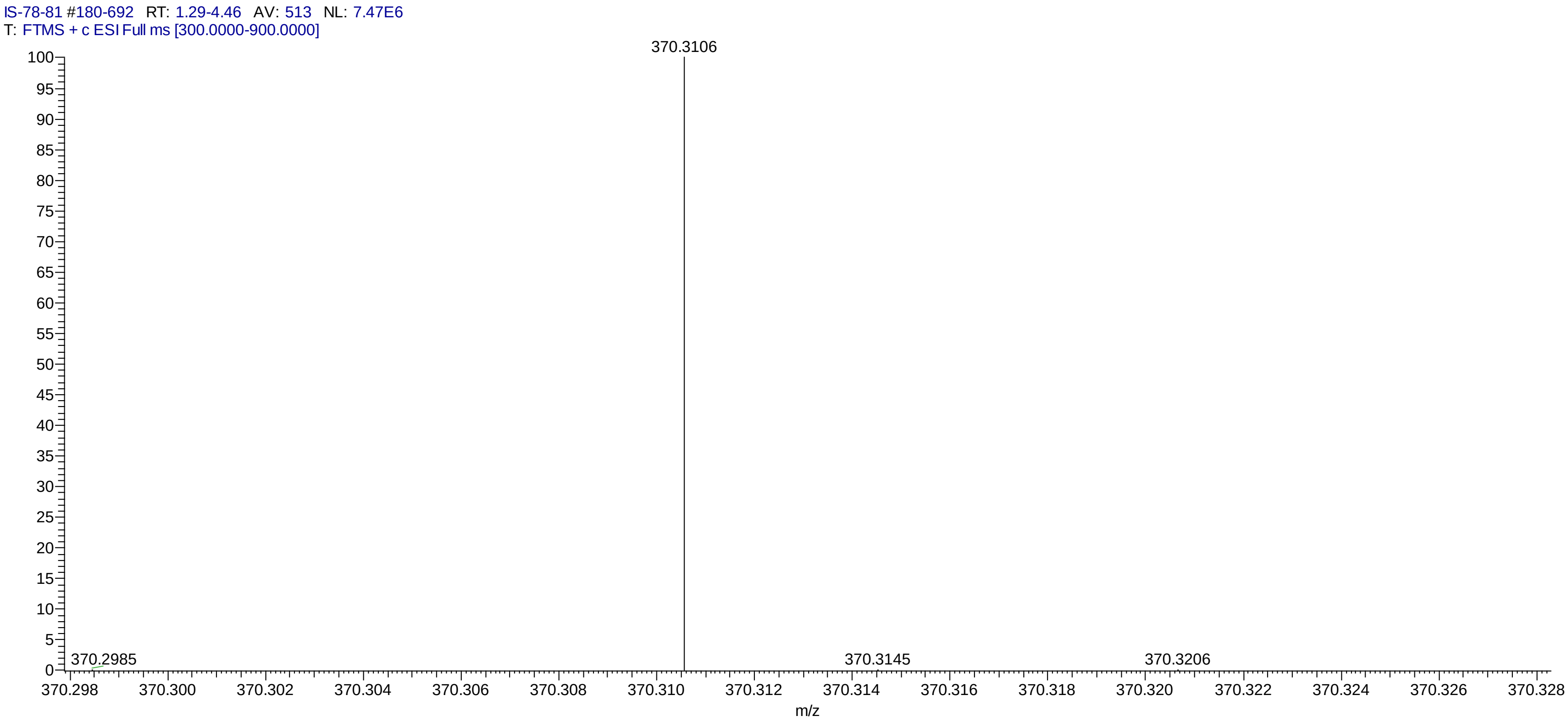

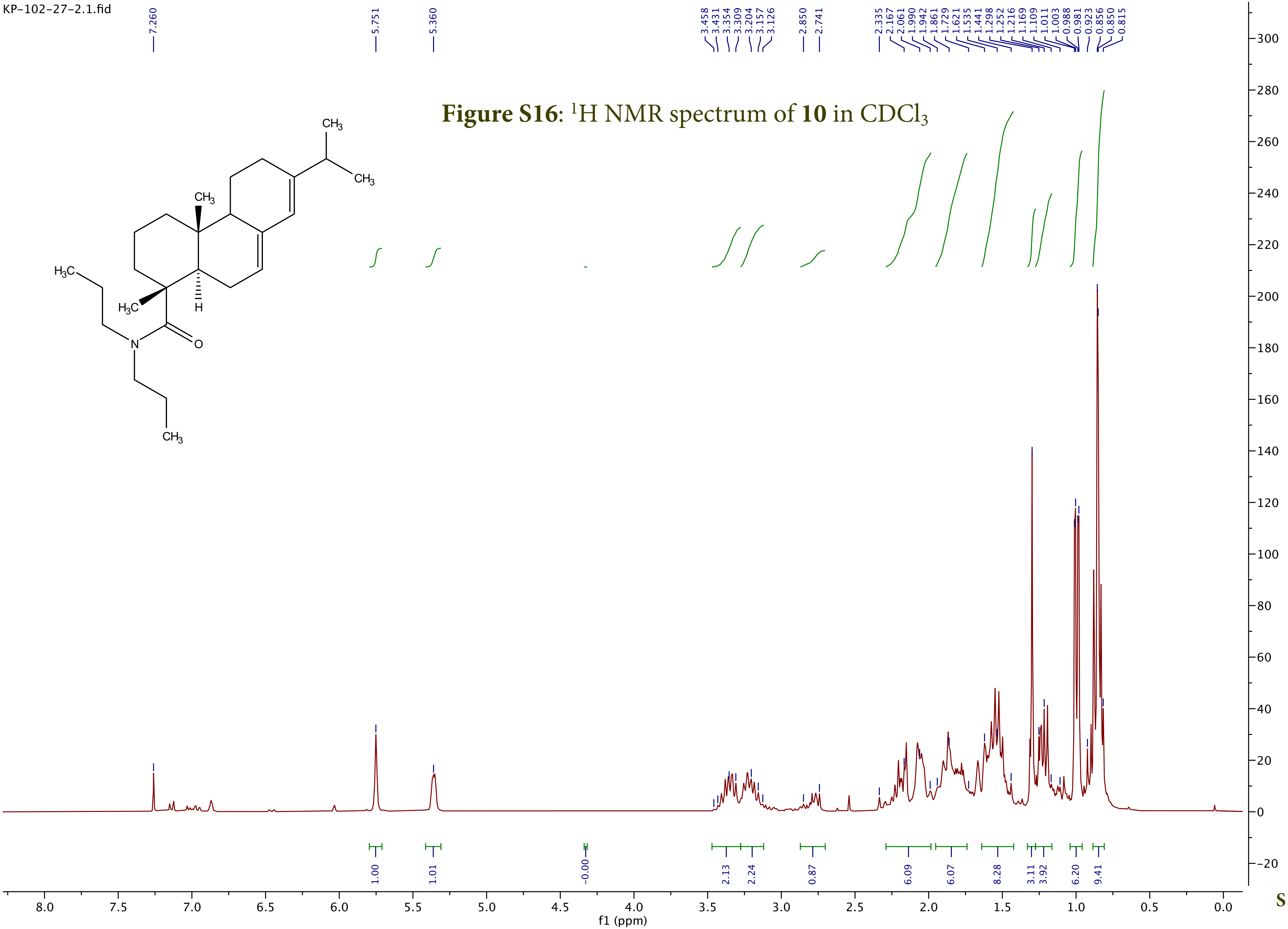

Figure S16: ^{1}H NMR spectrum of **10** in $CDCl_3$

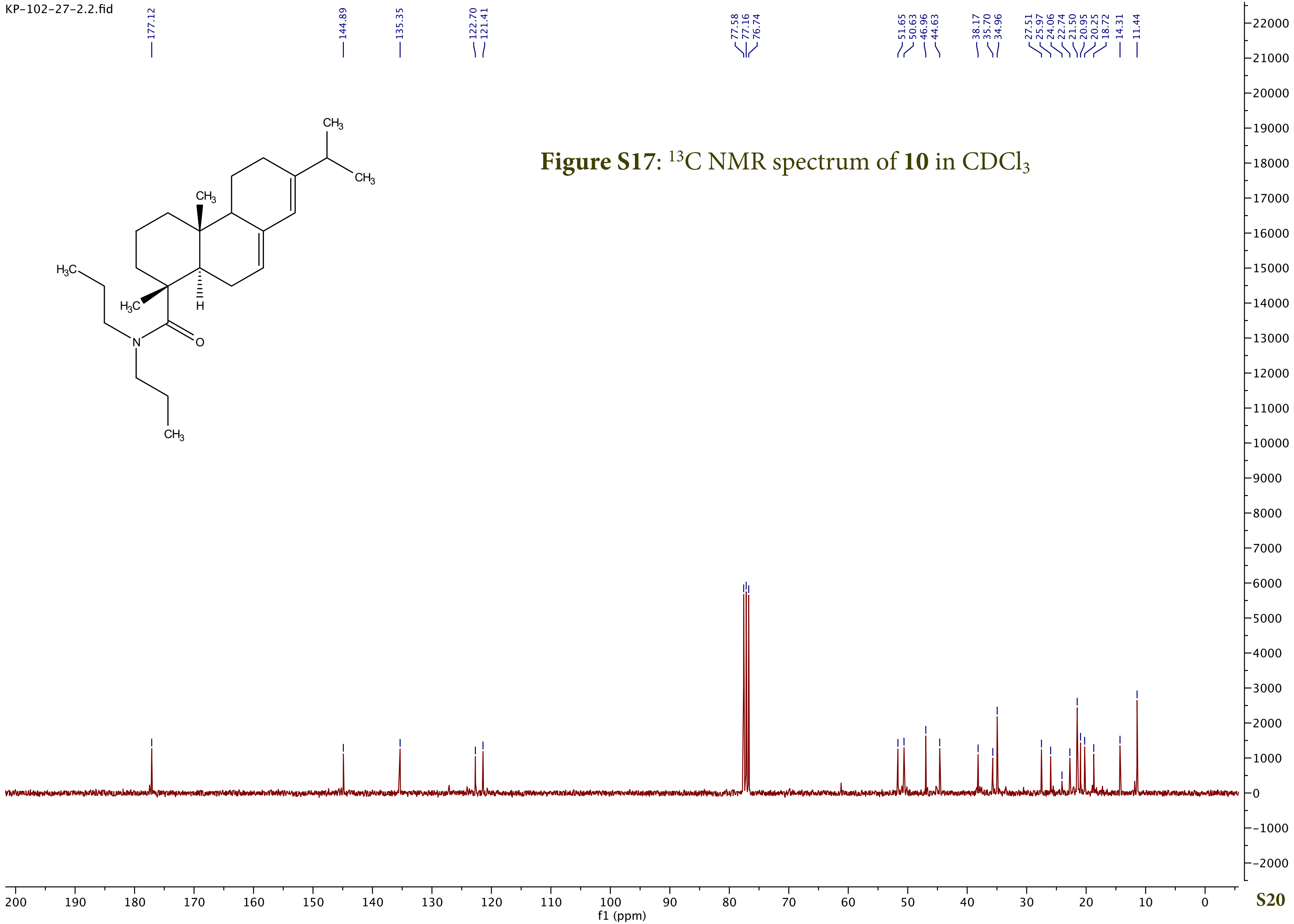

Figure S17: ^{13}C NMR spectrum of **10** in $CDCl_3$

Figure S18: High resolution mass spectrum of **10**

Sample Name	Mol Fomula	MW(COME WITH SAMPLES)	MW(CALCULATED)	M+H	observed	delta	ppm
**KP-102-27**	C26H43NO	385.3345	385.33447	386.3423	**386.3423**	0.0000	0.00

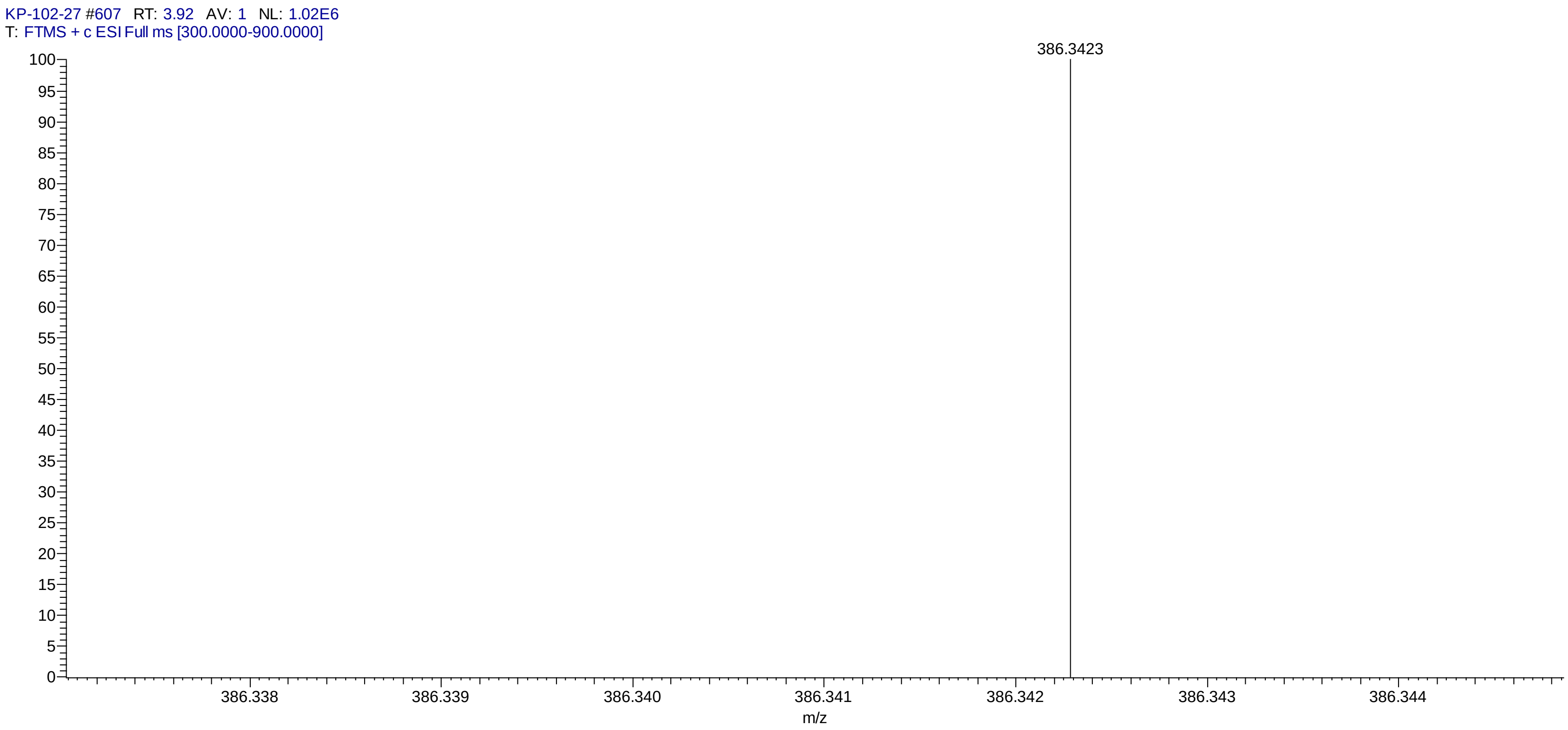

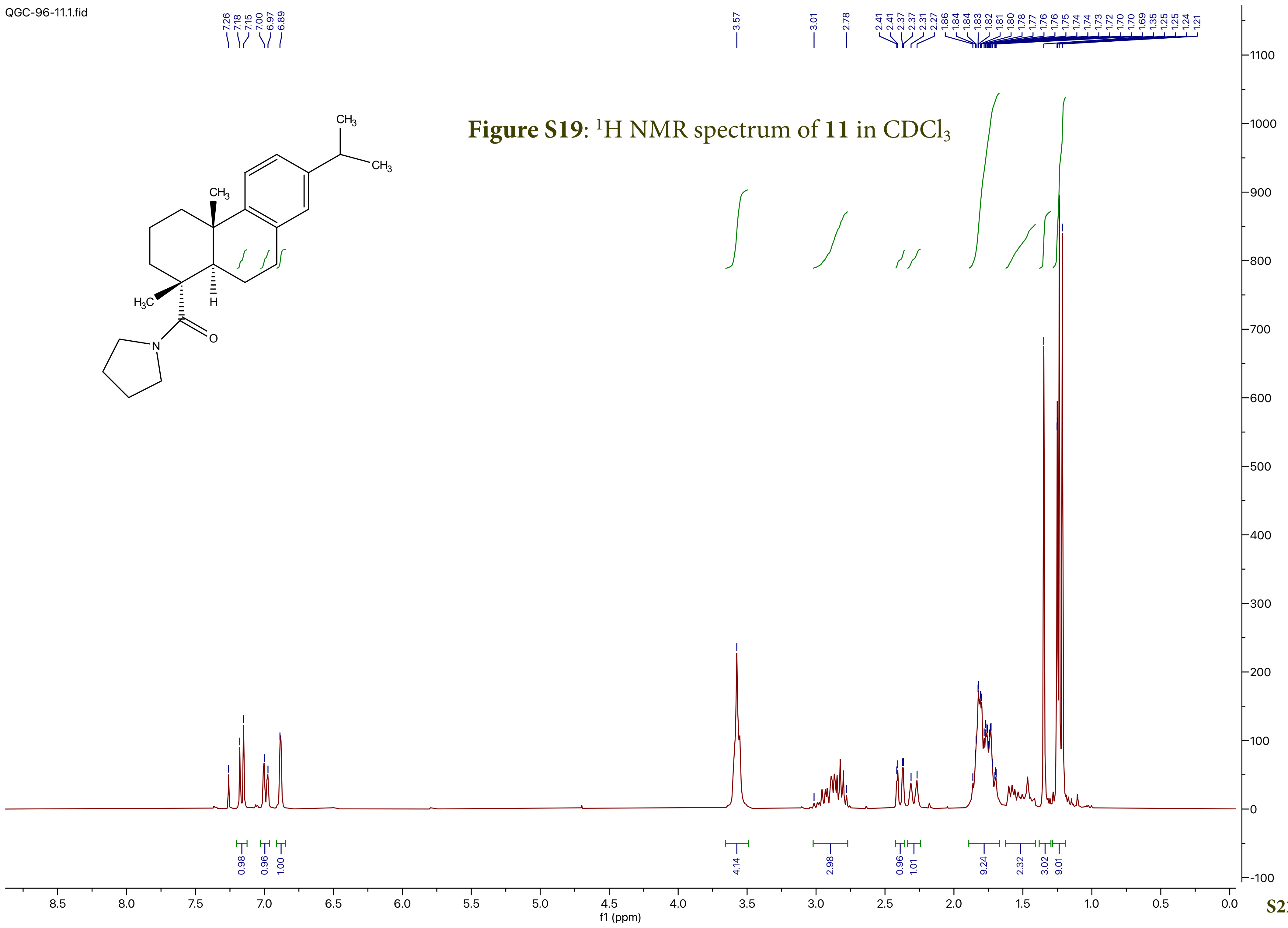

Figure S19: ^{1}H NMR spectrum of **11** in $CDCl_3$

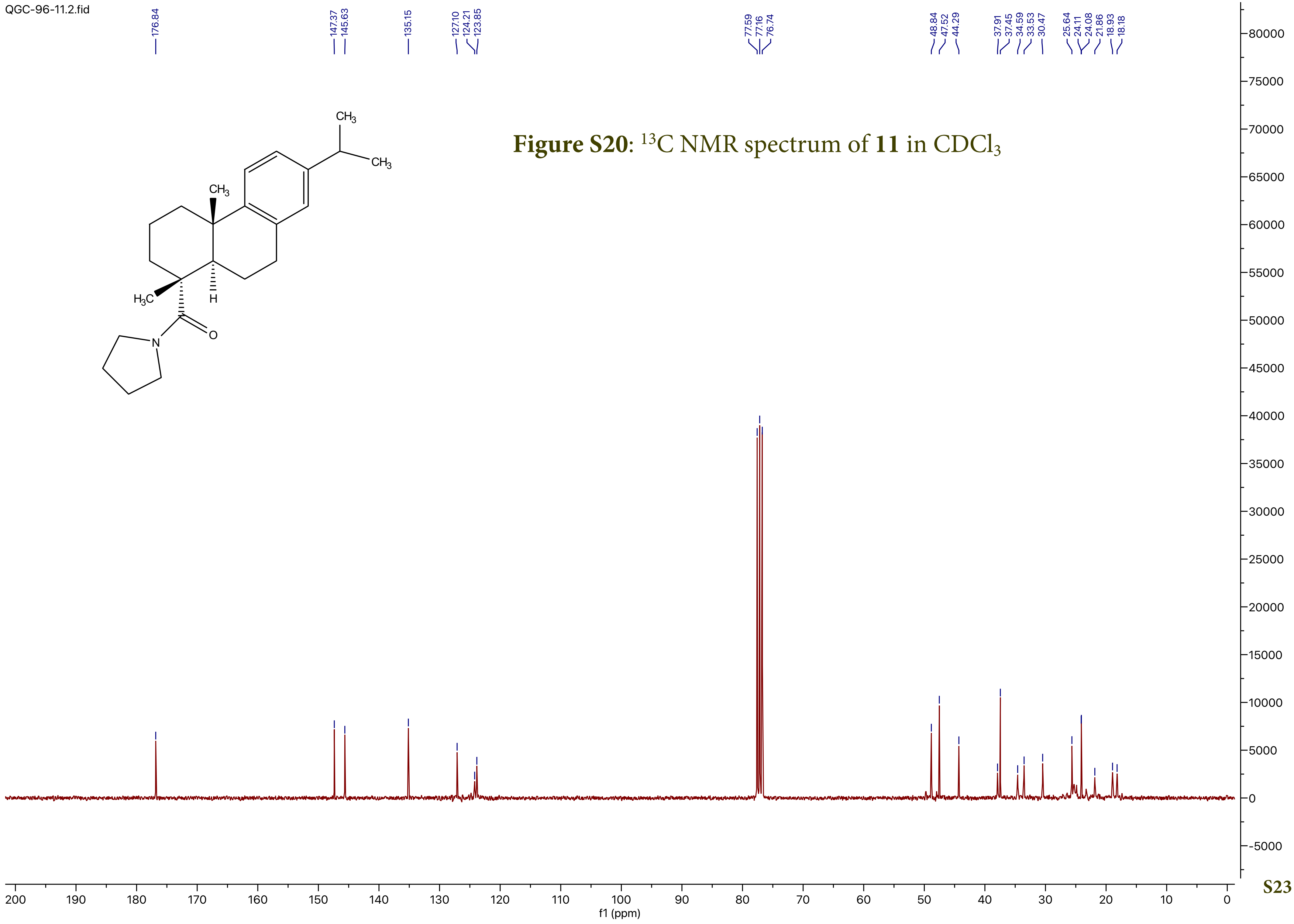

Figure S20: ^{13}C NMR spectrum of **11** in $CDCl_3$

Figure S21: High resolution mass spectrum of **11**

Sample Name	Mol Fomula	MW	M+H	observed	delta	ppm
IS-78-67	C24H35NO	353.2719	354.2797	354.2796	-0.0001	-0.17

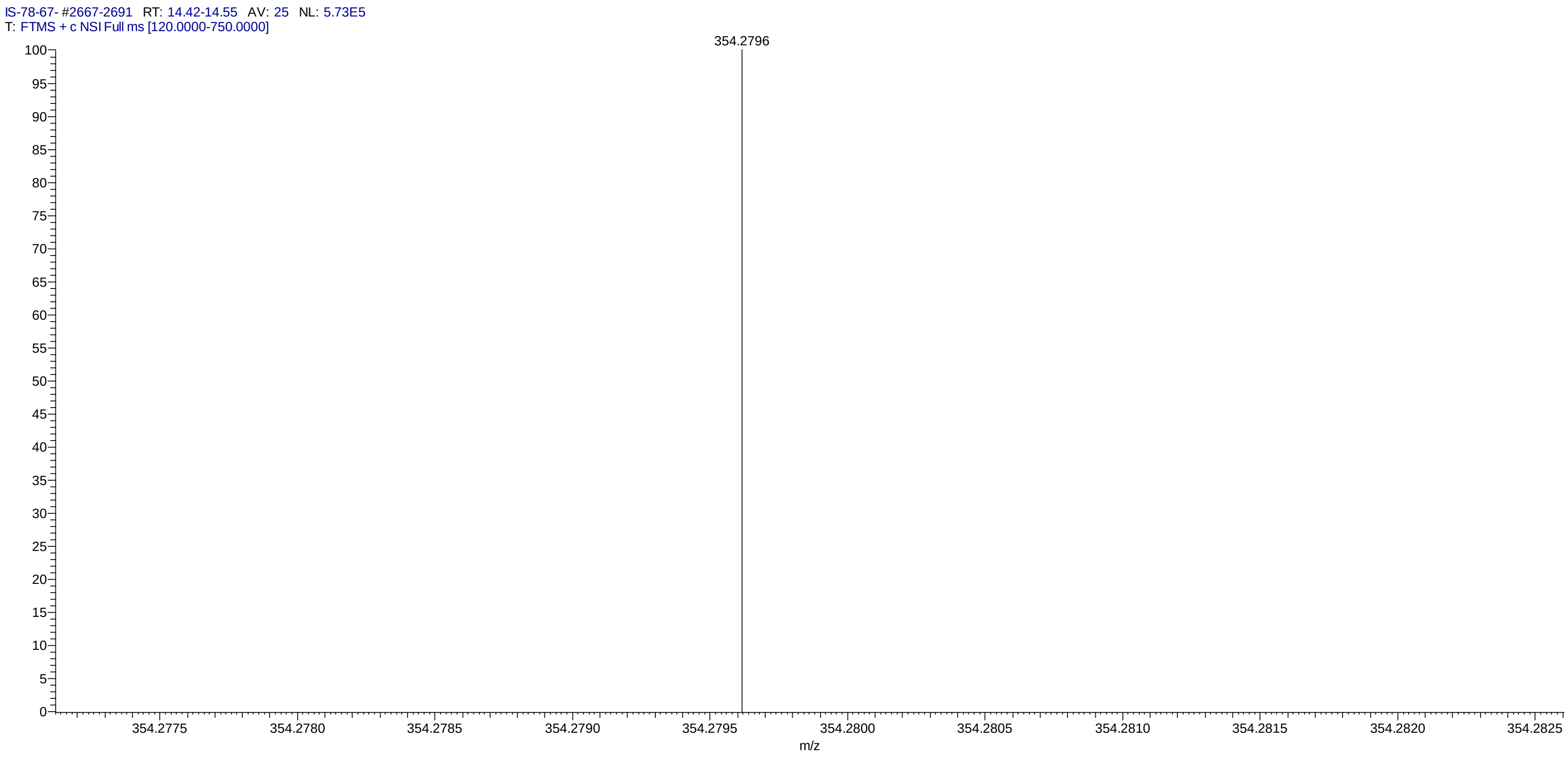

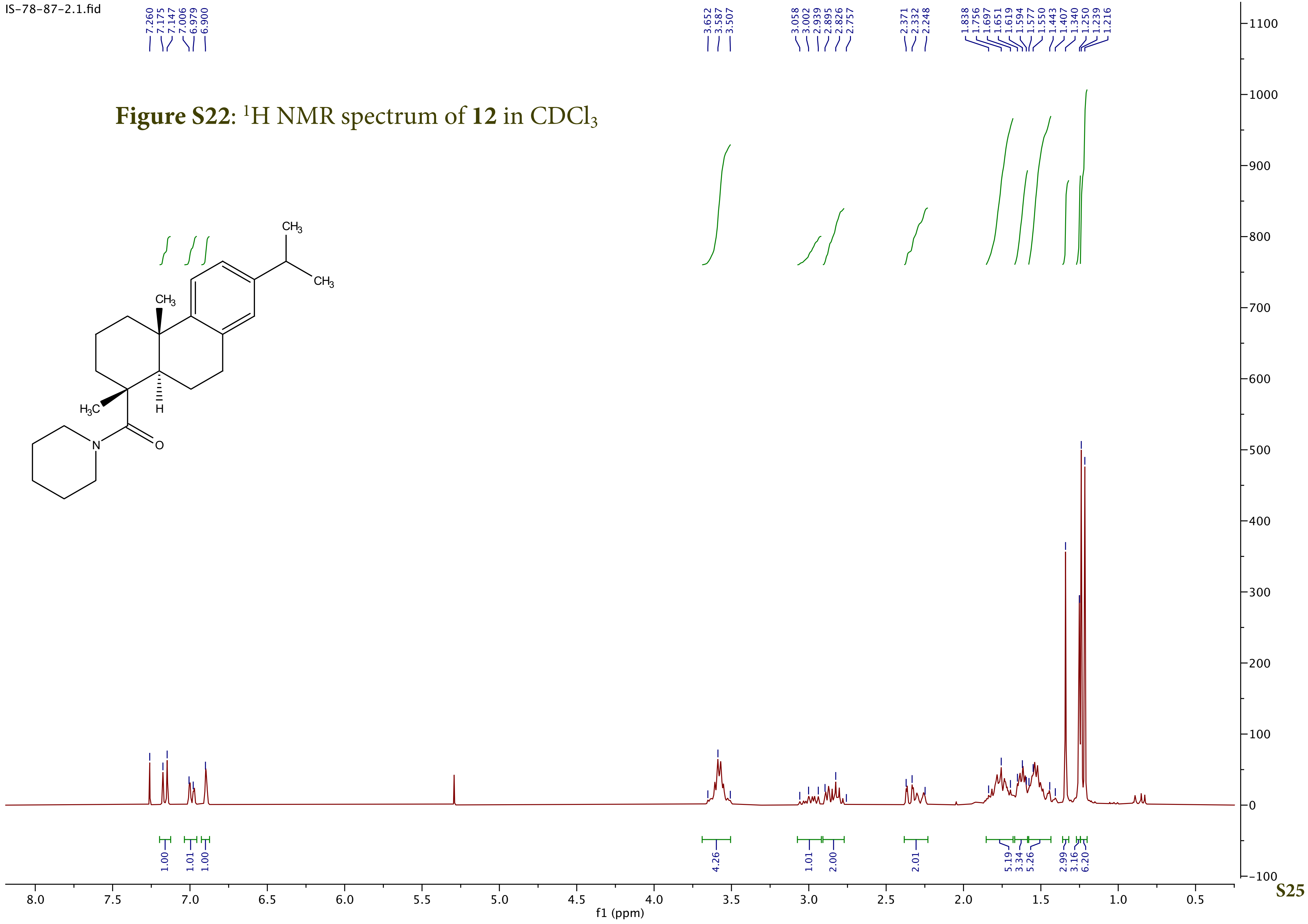

Figure S22: ^{1}H NMR spectrum of **12** in $CDCl_3$

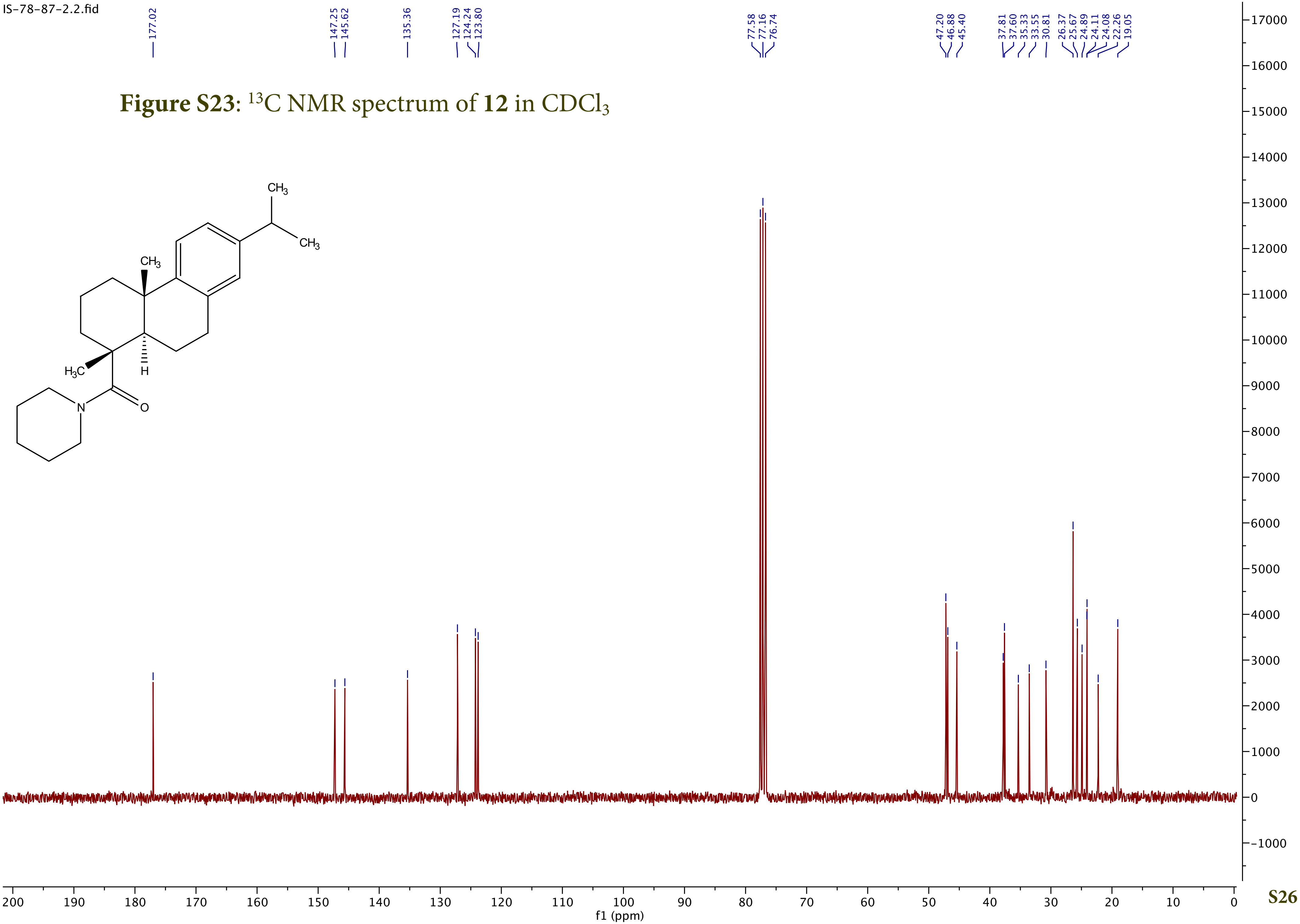

Figure S23: ^{13}C NMR spectrum of **12** in $CDCl_3$

Figure S24: High resolution mass spectrum of **12**

Sample Name	Mol Fomula	MW	M+H	observed	delta	ppm
IS-78-69	C25H37NO	367.2875	368.2953	368.2948	-0.0005	-1.38

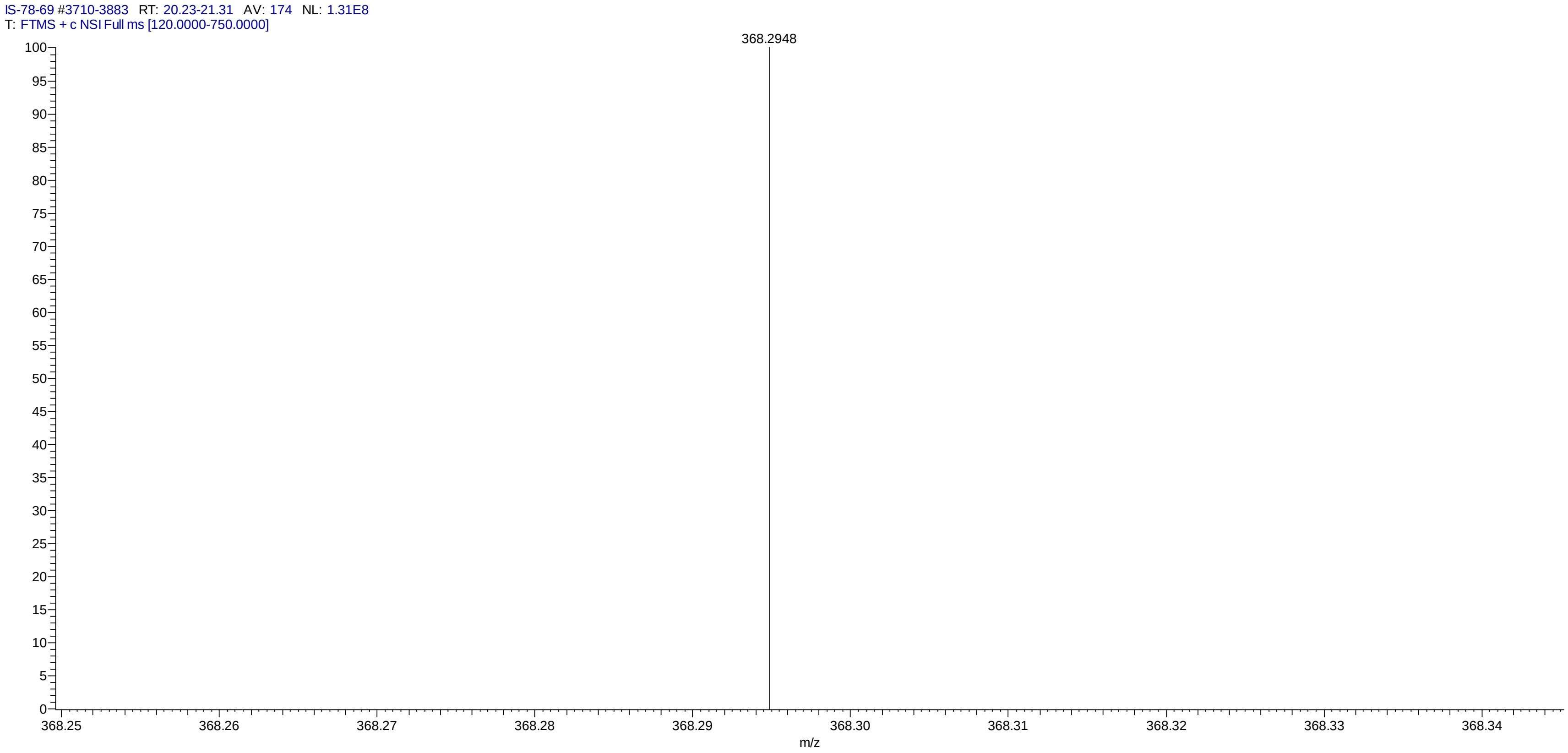

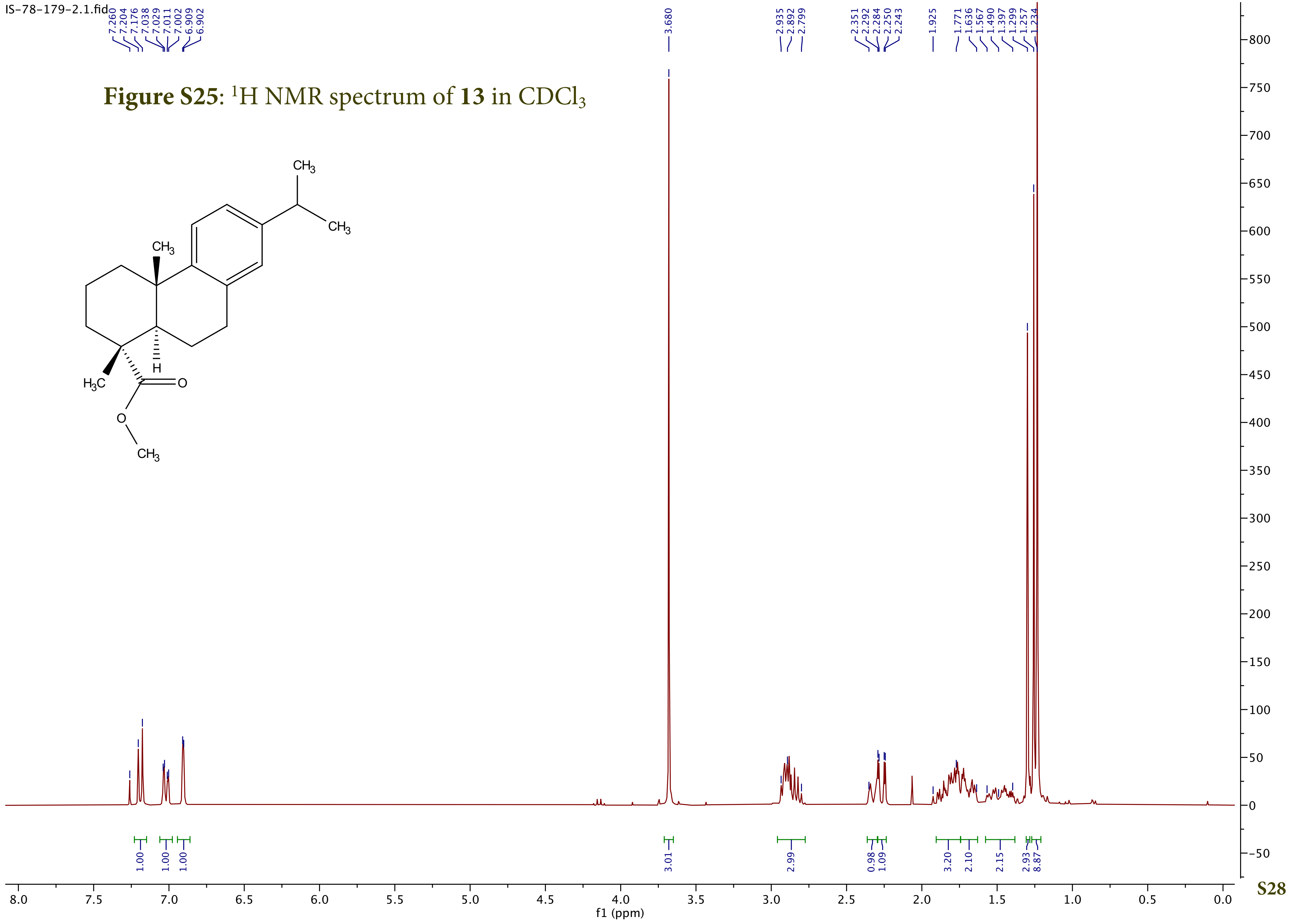

Figure S25: ^{1}H NMR spectrum of **13** in $CDCl_3$

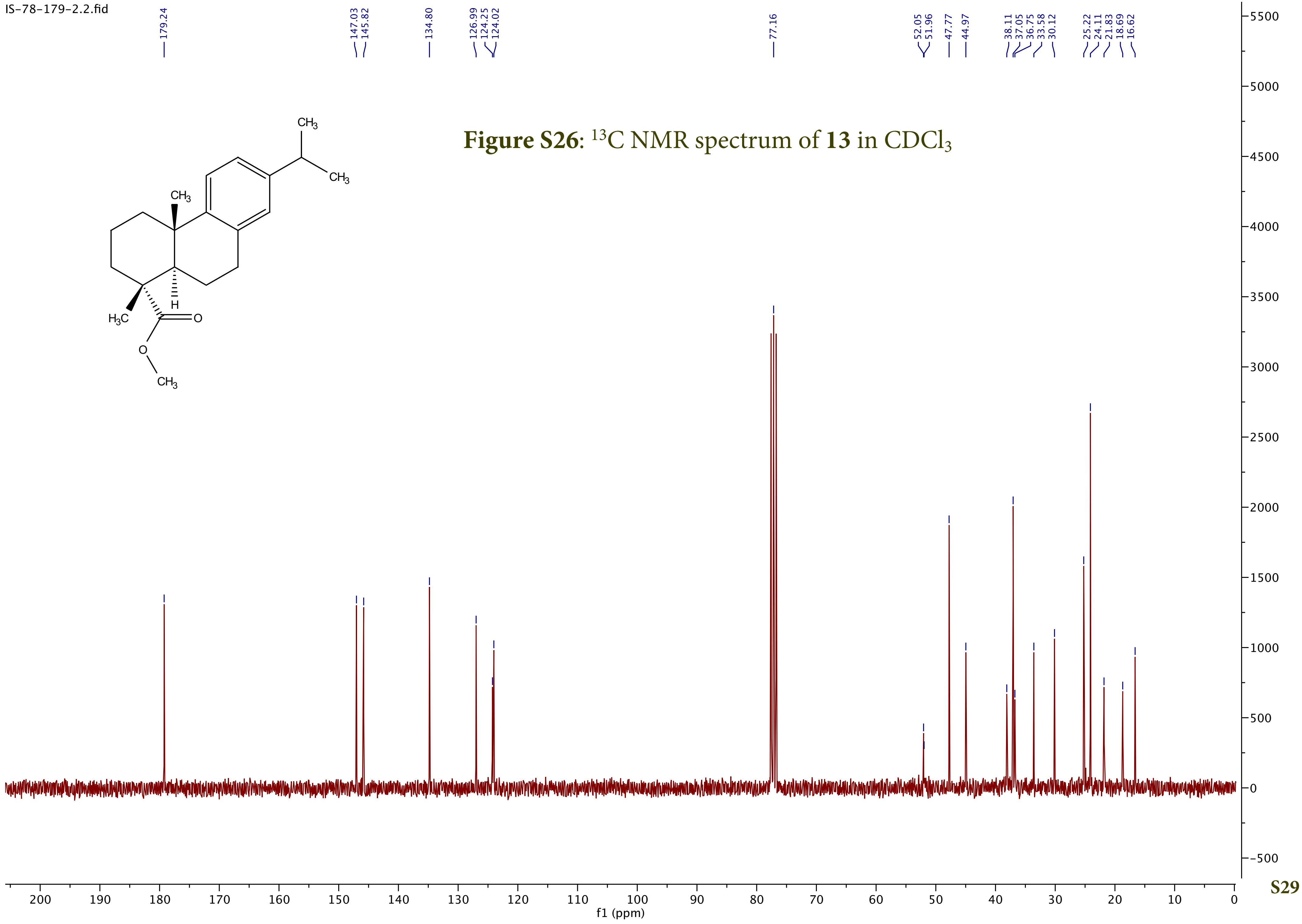

Figure S26: ^{13}C NMR spectrum of **13** in $CDCl_3$

Figure S27: High resolution mass spectrum of **13**

Sample Name	Mol Fomula	MW	M+H	observed	delta	ppm
IS-78-179-2	C21H31O2	314.2246	315.2324	315.2321	-0.0003	-0.92

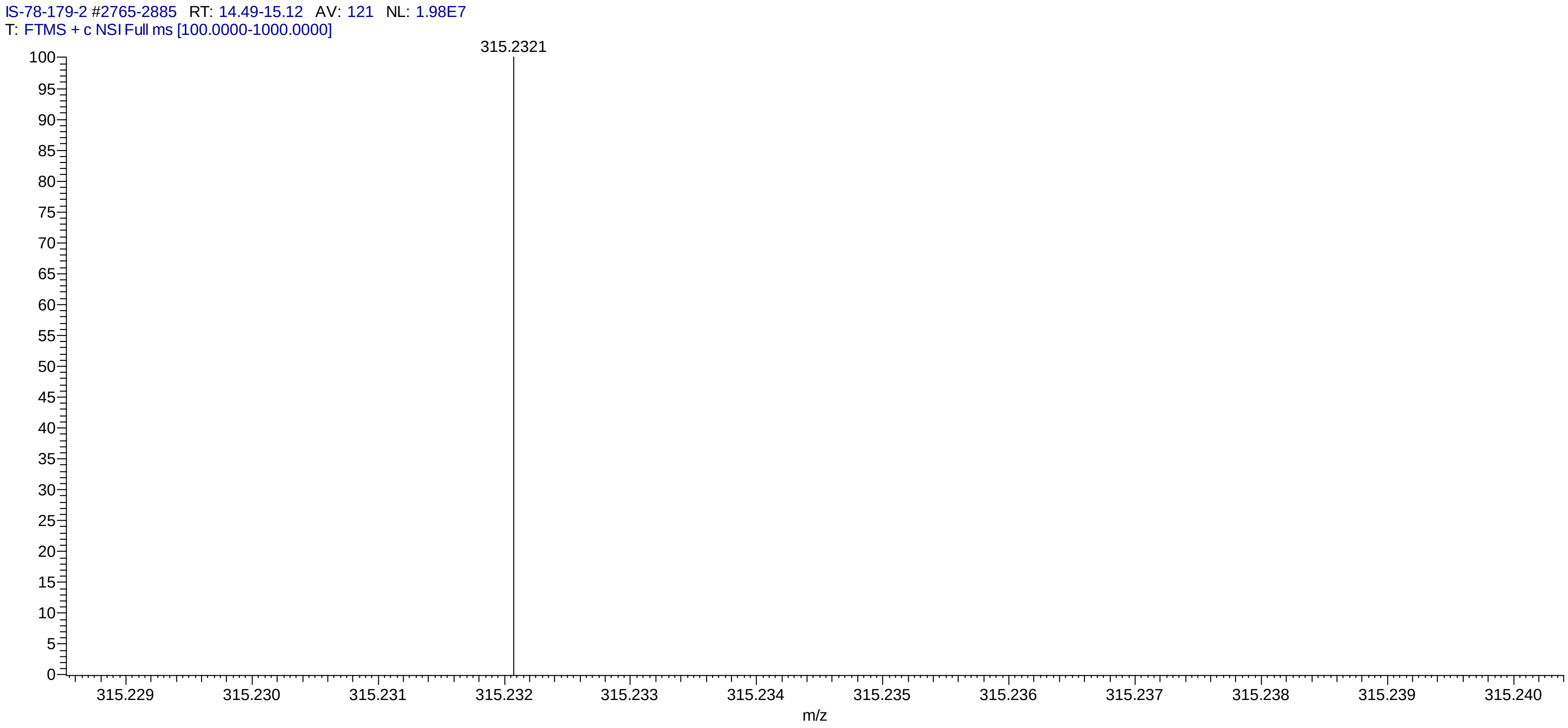

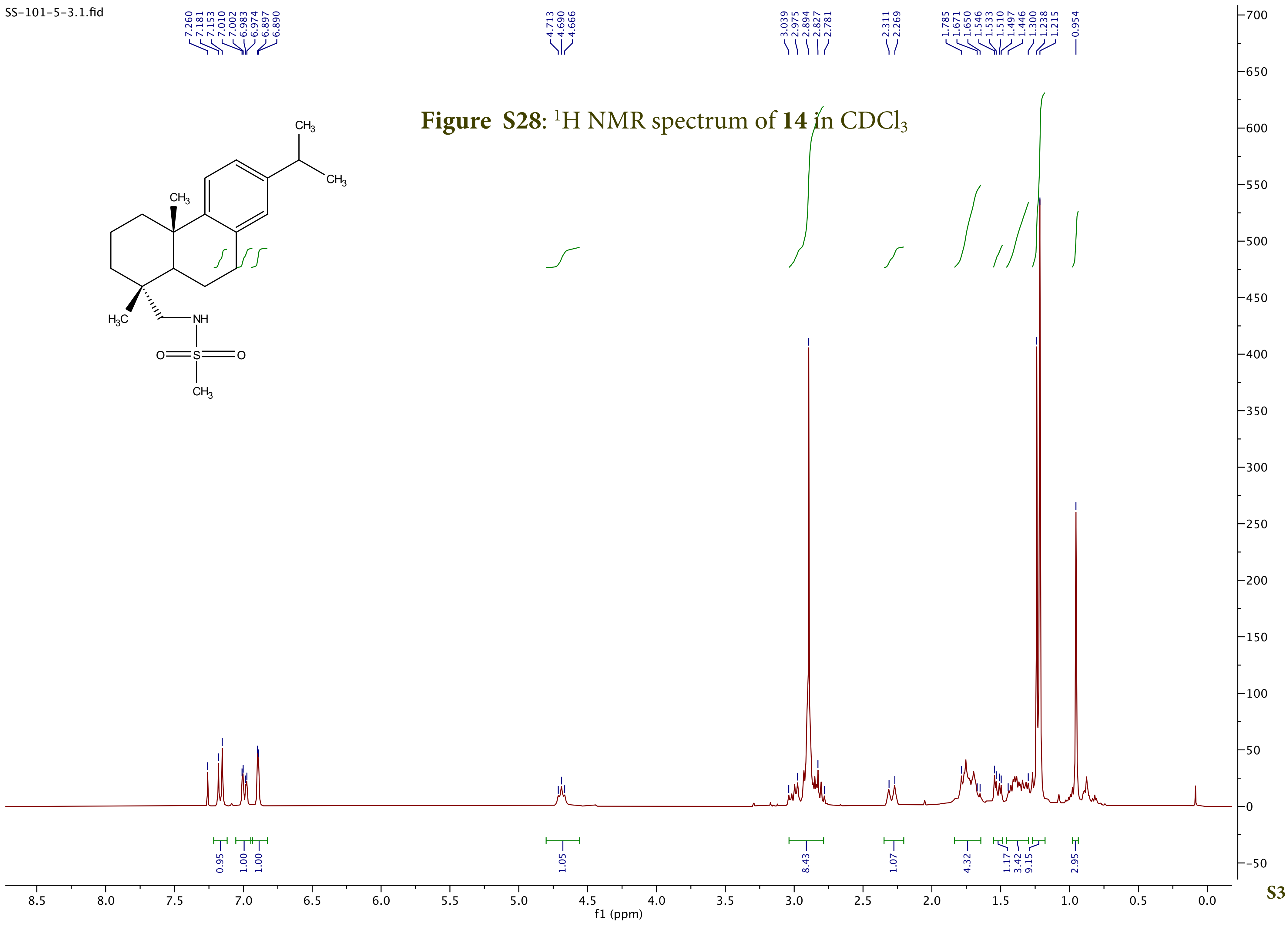

Figure S28: ^{1}H NMR spectrum of **14** in $CDCl_3$

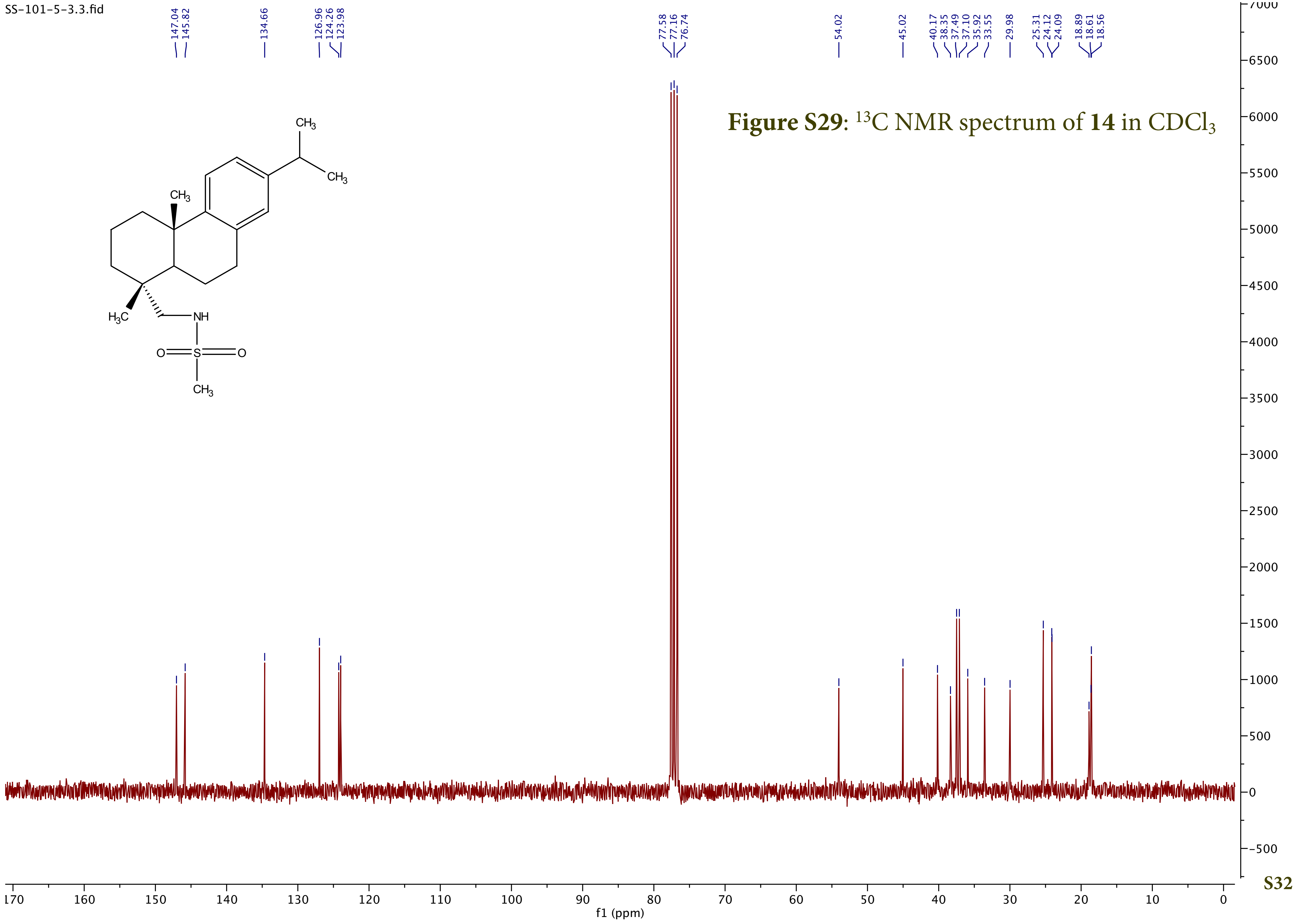

Figure S29: ^{13}C NMR spectrum of **14** in $CDCl_3$

Figure S30: High resolution mass spectrum of **14**

Sample Name	Mol Fomula	MW	M+H	observed	delta	ppm
SS-101-5-3	C21H33NO2S	363.2232	364.2310	364.2303	-0.0007	-1.92

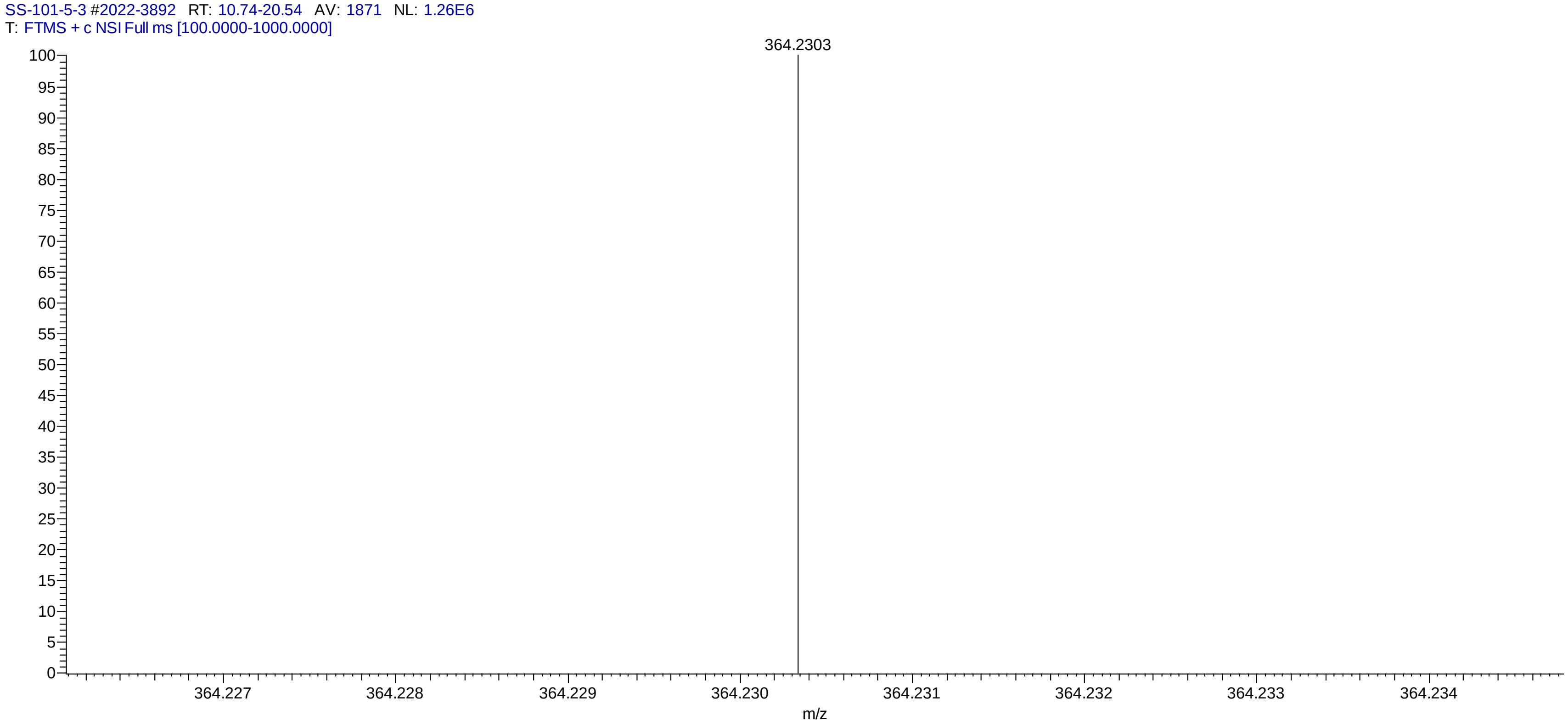

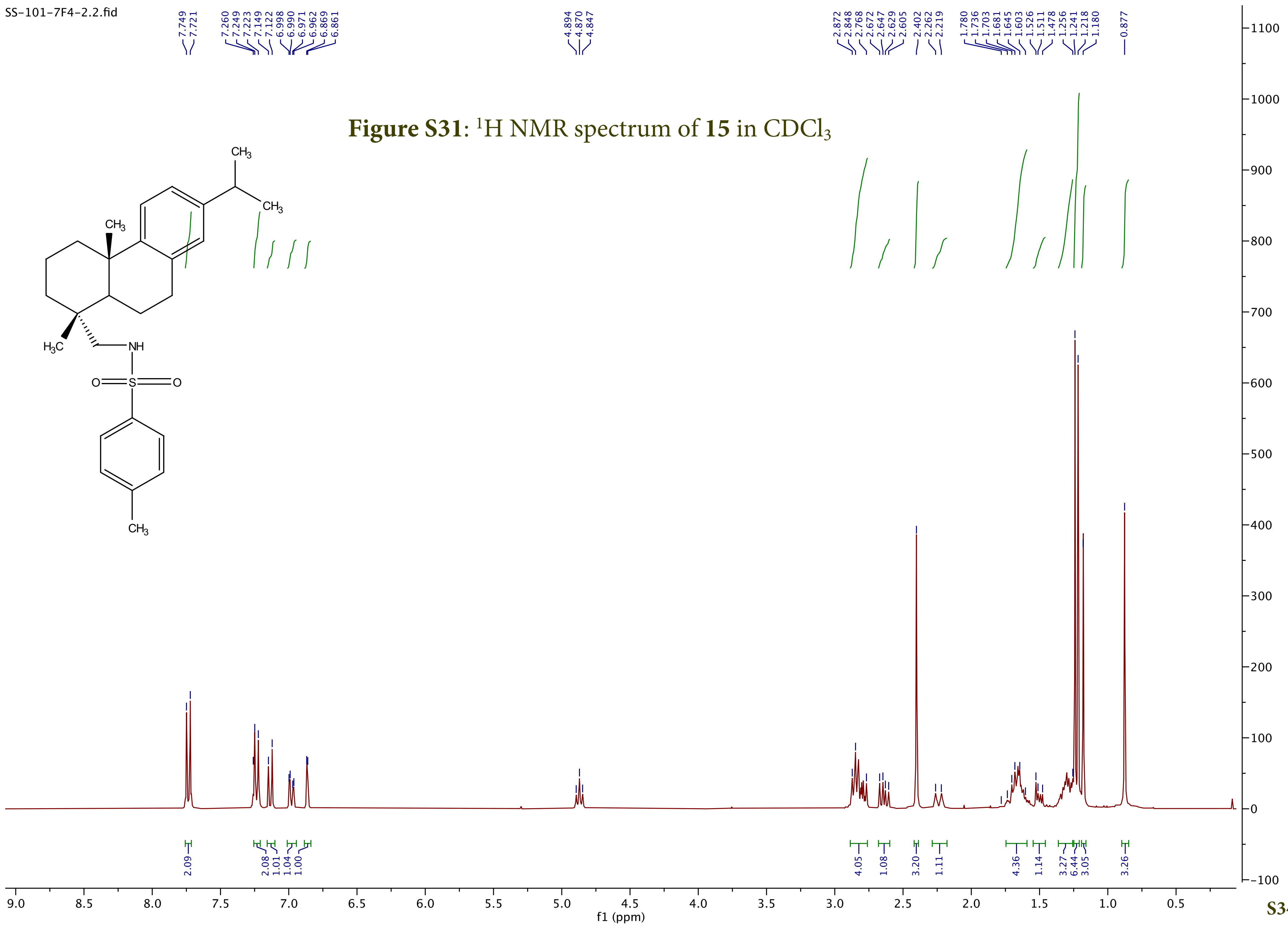

Figure S31: ^{1}H NMR spectrum of **15** in $CDCl_3$

SS-101-7F4-2.4.fid

Figure S32: ^{13}C NMR spectrum of **15** in $CDCl_3$

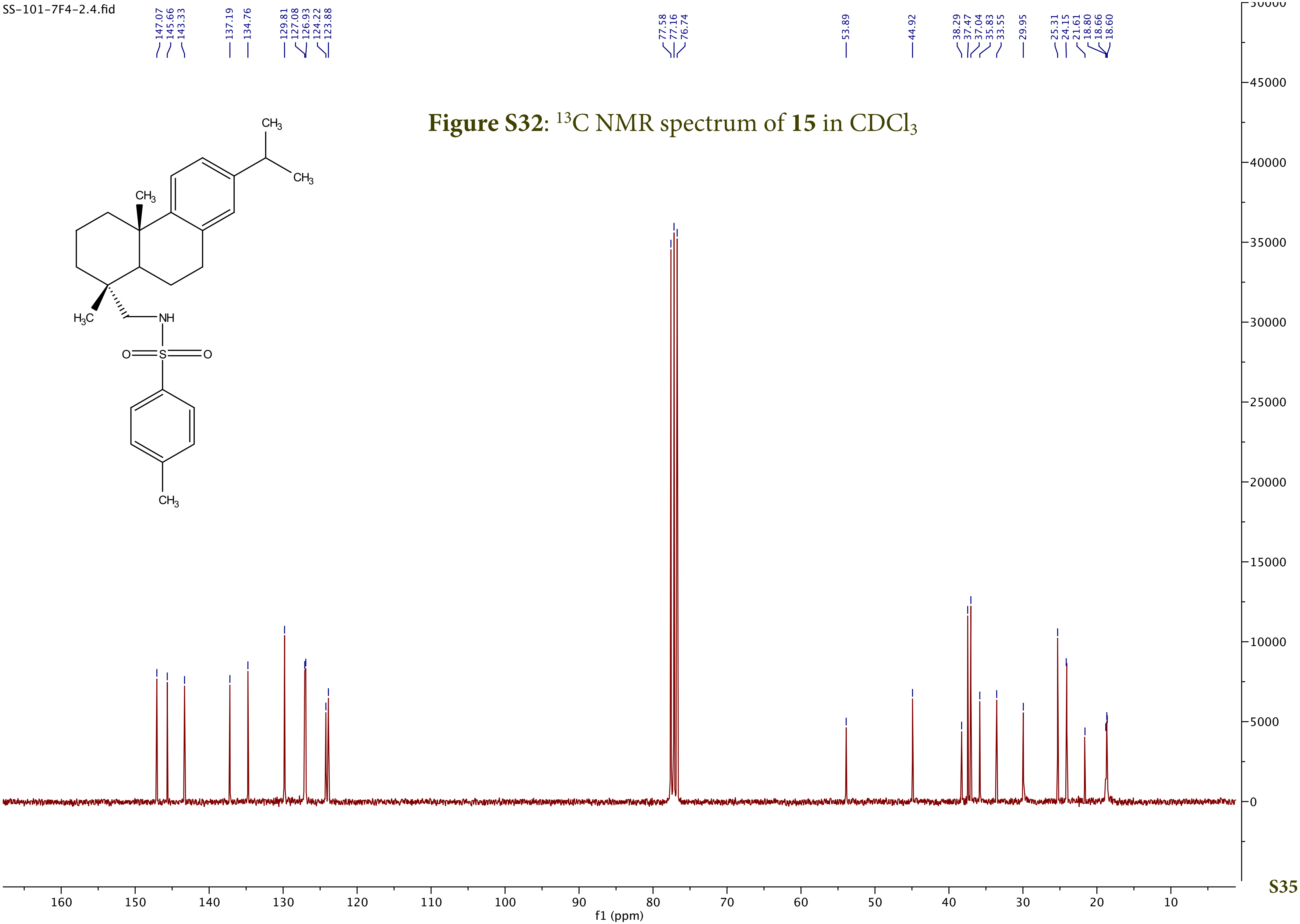

Figure S33: High resolution mass spectrum of **15**

Sample Name	Mol Fomula	MW	M+H	observed	delta	ppm
SS-101-7-4	C27H37NO2S	439.2545	440.2623	440.2616	-0.0007	-1.59

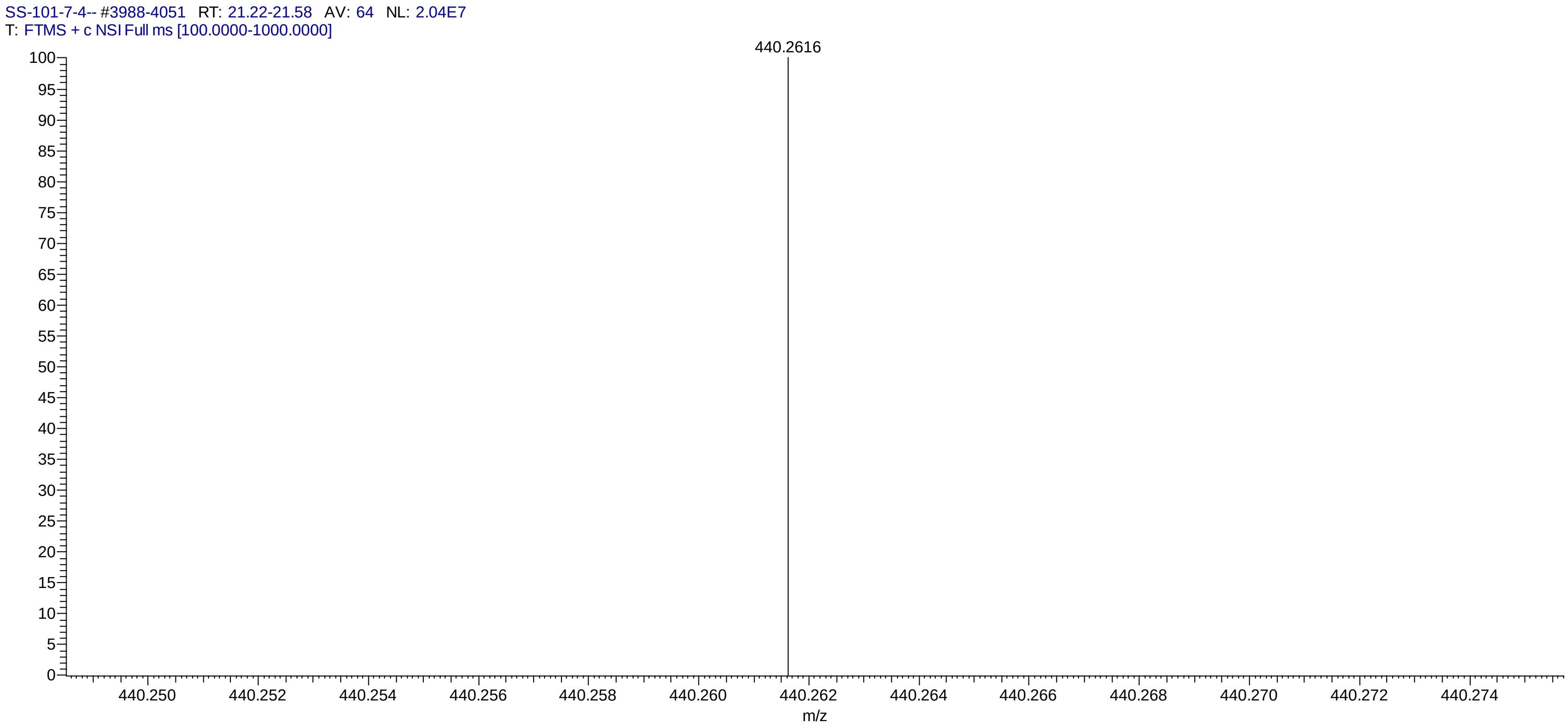

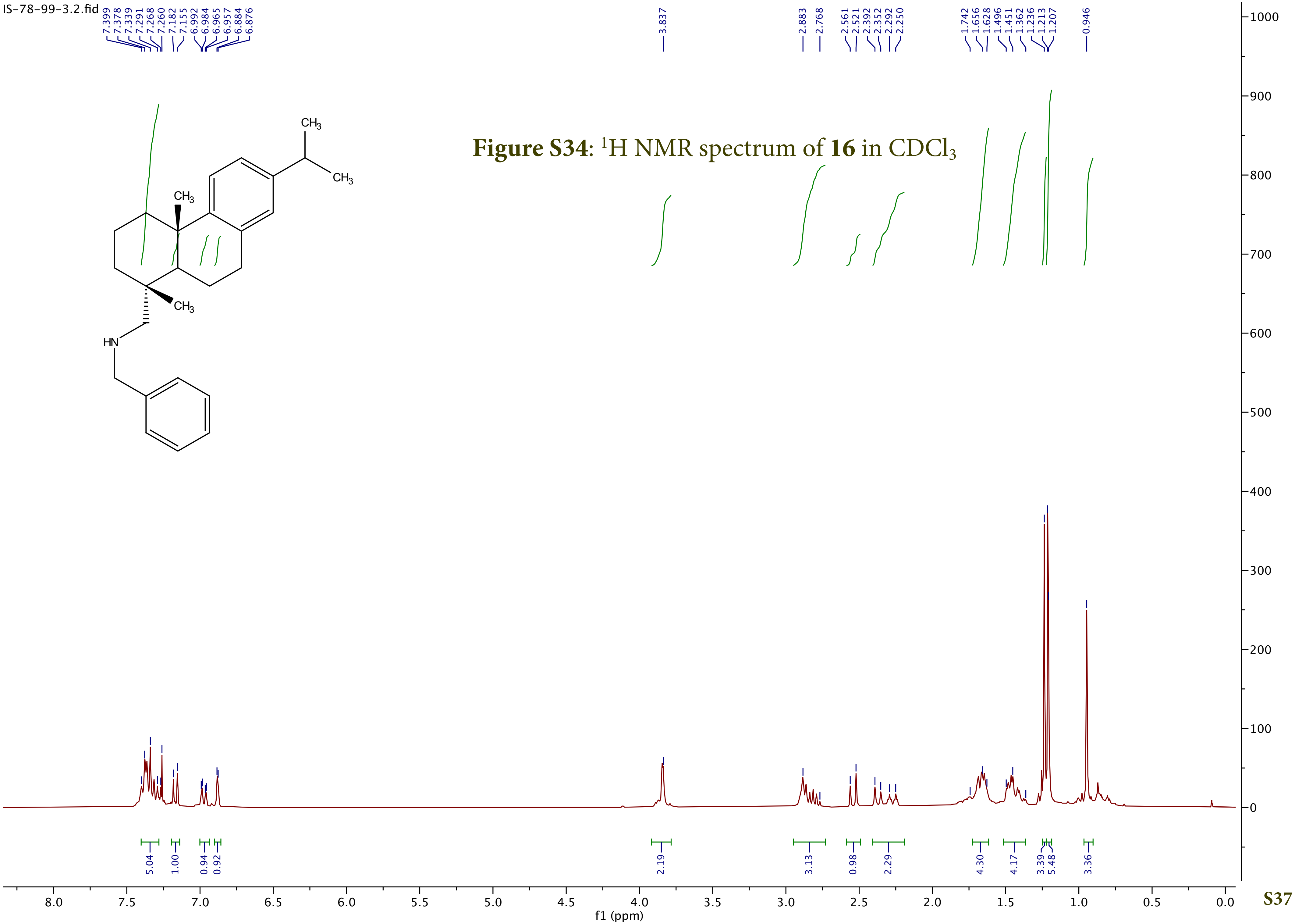

Figure S34: ^{1}H NMR spectrum of **16** in $CDCl_3$

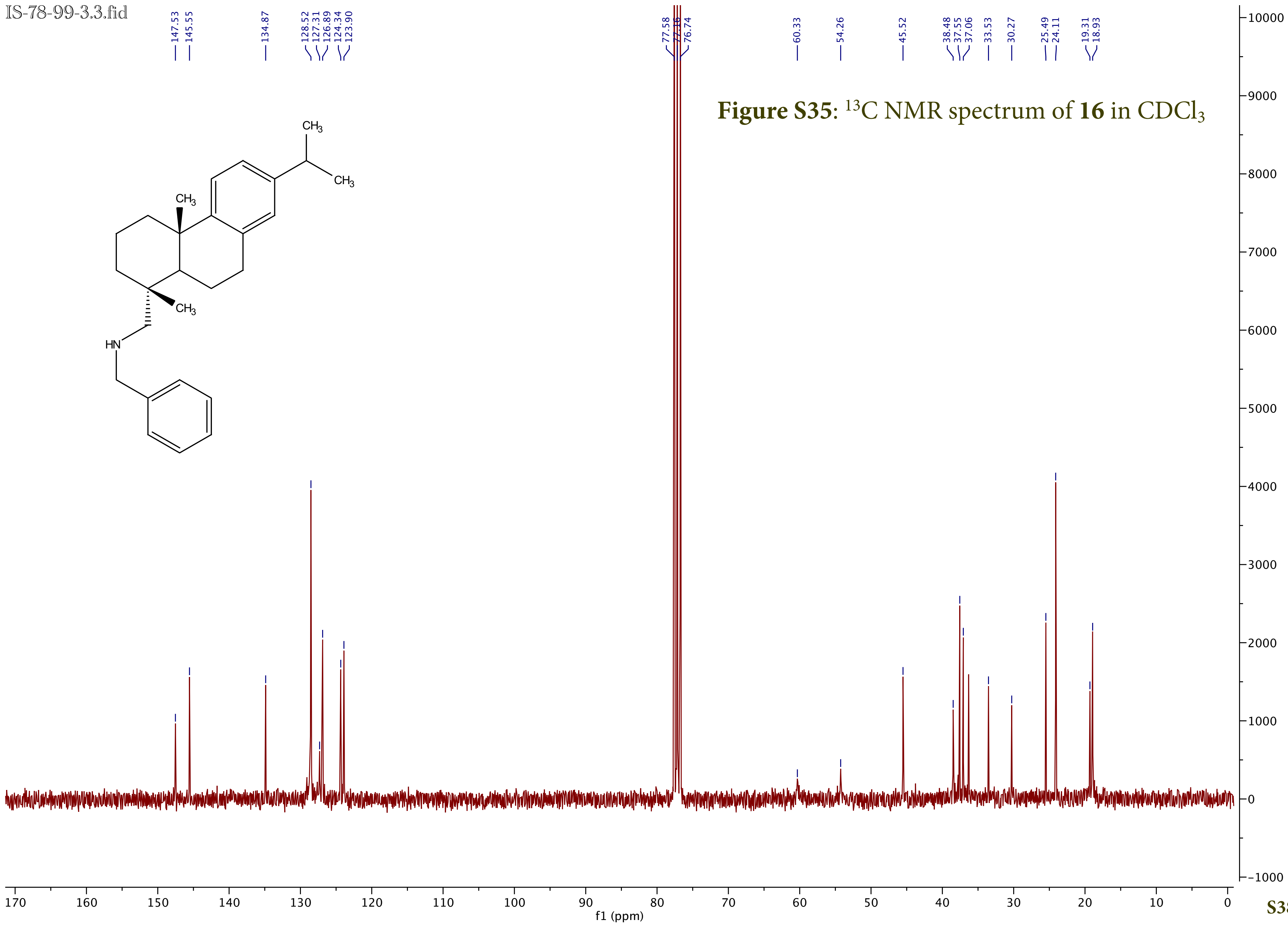

Figure S35: ^{13}C NMR spectrum of **16** in $CDCl_3$

Figure S36: High resolution mass spectrum of **16**

Sample Name	Mol Fomula	MW	M+H	observed	delta	ppm
IS-78-99-3	C27H37N	375.2926	376.3004	376.3001	-0.0003	-0.77

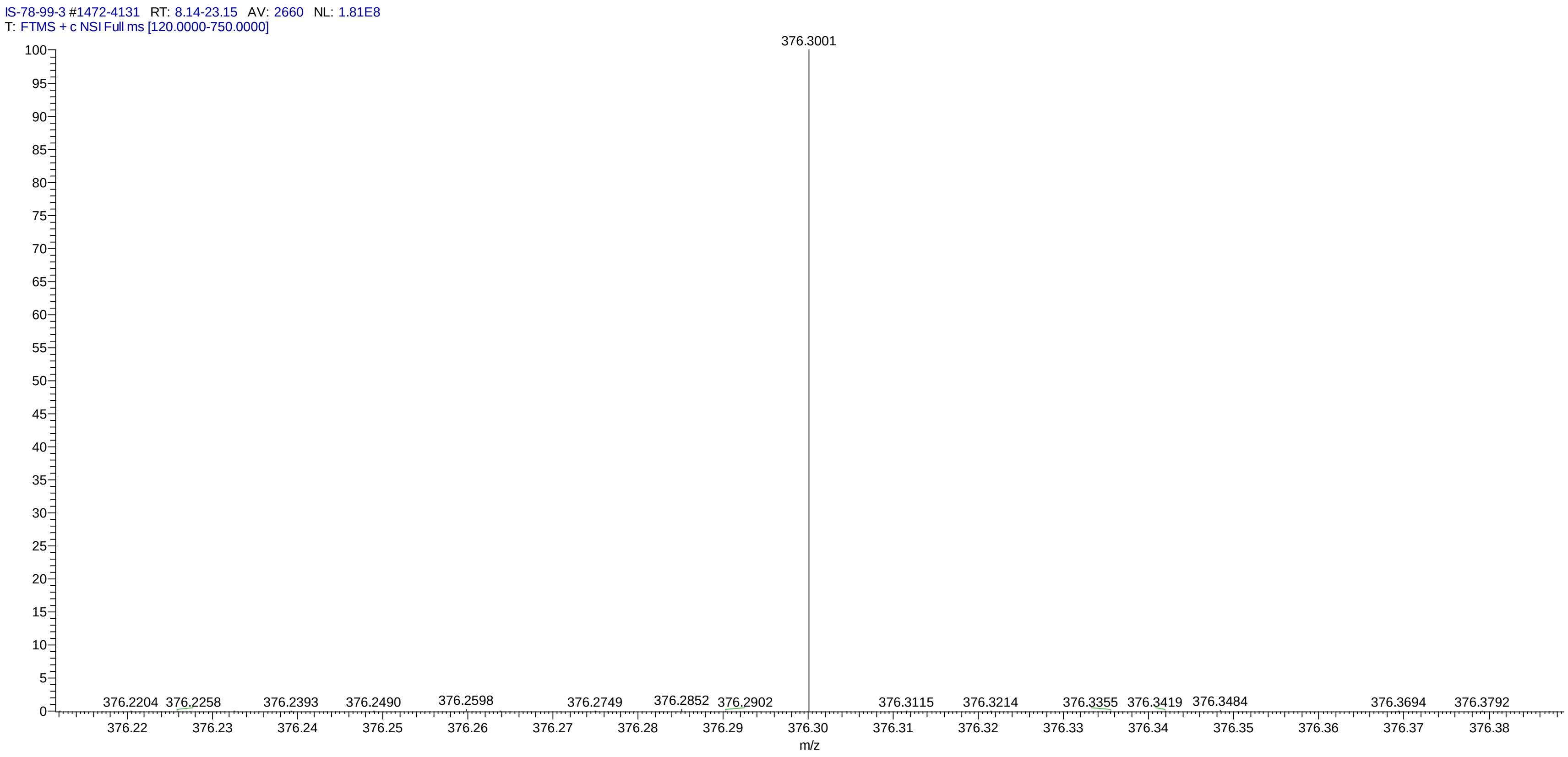

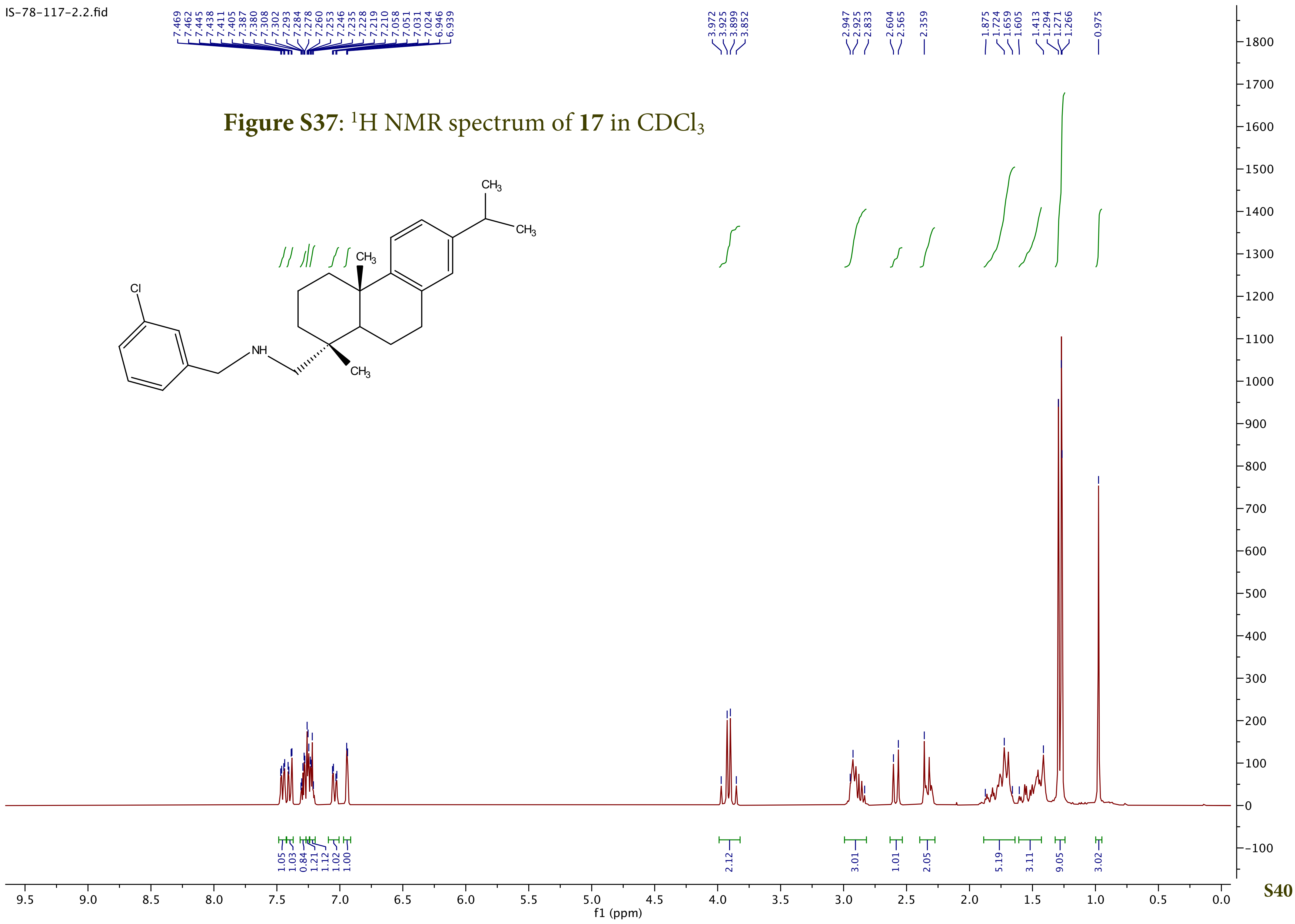

Figure S37: ^{1}H NMR spectrum of **17** in $CDCl_3$

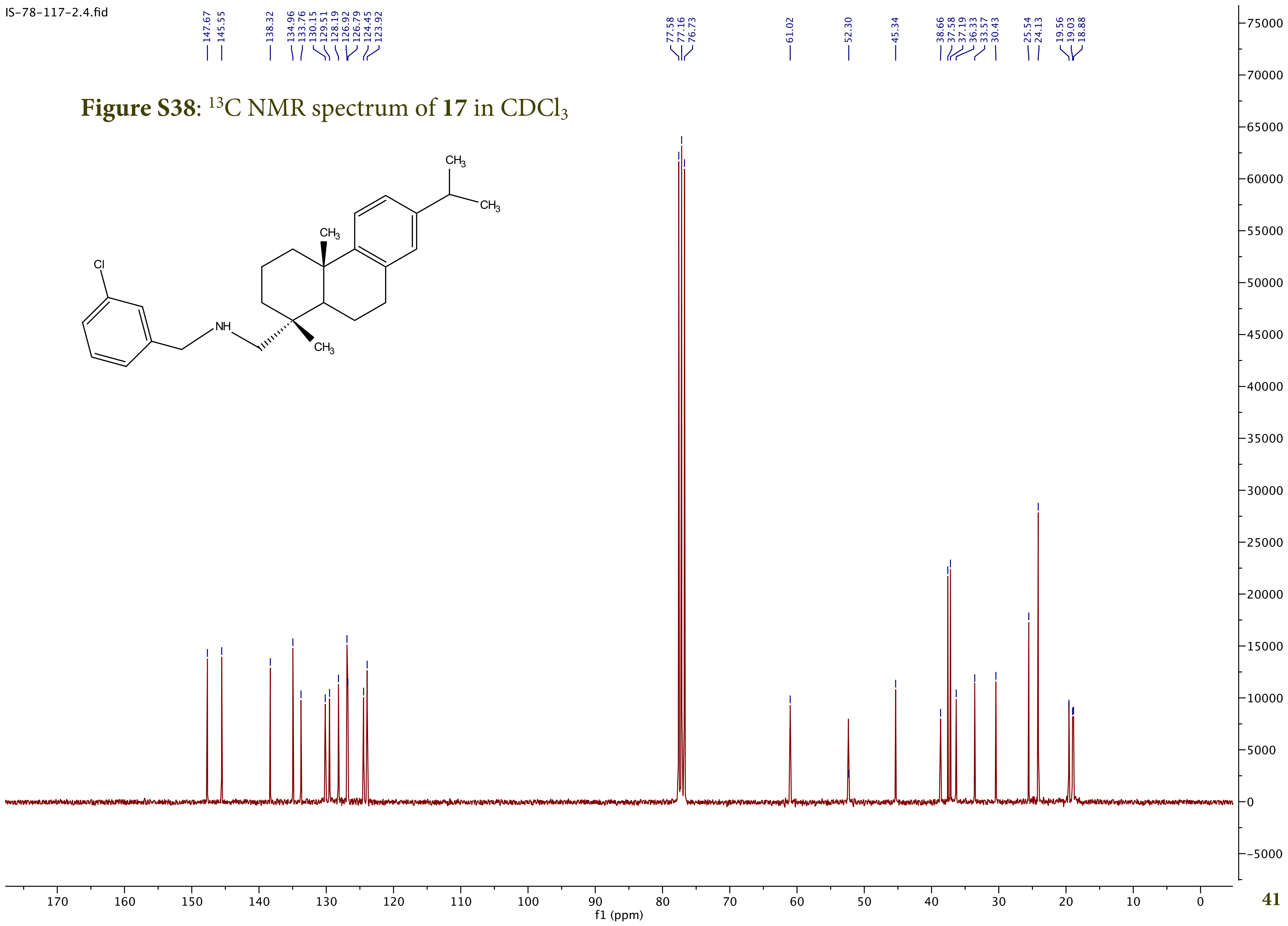

Figure S38: ^{13}C NMR spectrum of **17** in $CDCl_3$

Figure S39: High resolution mass spectrum of **17**

Sample Name	Mol Fomula	MW(COME WITH SAMPLES)	MW(CALCULATED)	M+H	observed	delta	ppm
**IS-78-117**	C27H36ClN	409.2536	409.25362	410.2614	**410.2611**	-0.0003	-0.78
			411.25067	412.2585	**412.2575**	-0.0010	-2.35

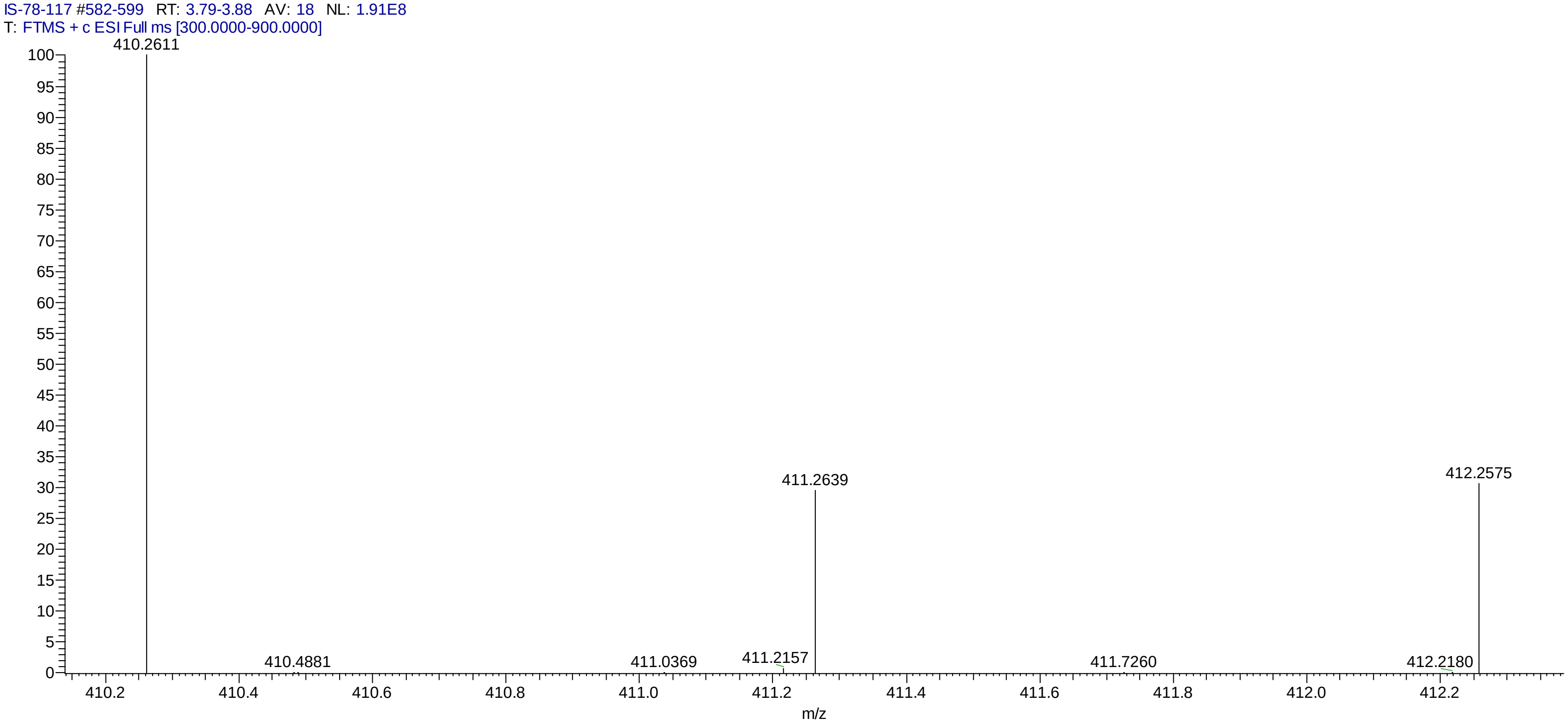

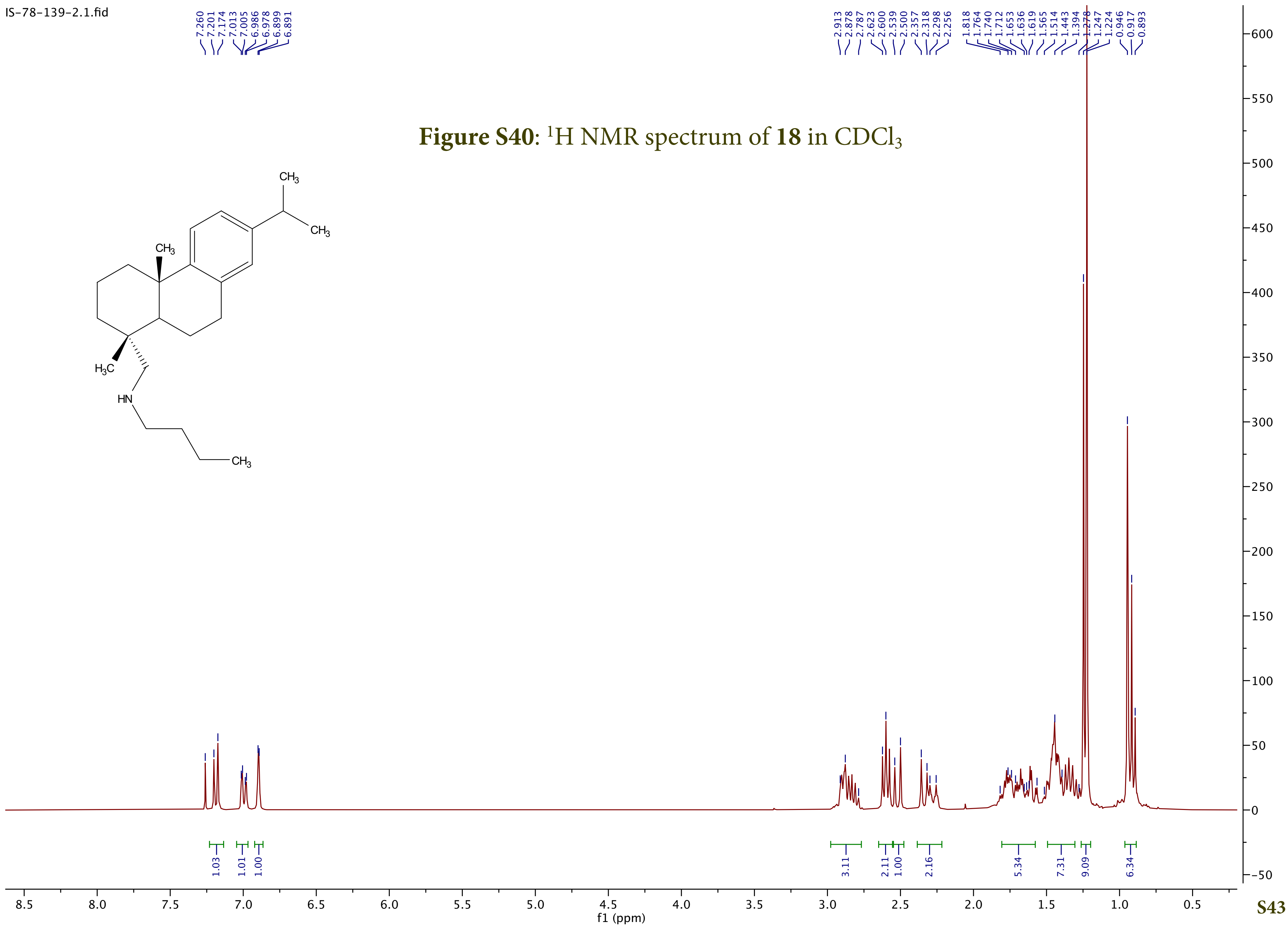

Figure S40: ^{1}H NMR spectrum of **18** in $CDCl_3$

IS-78-139-2.4.fid

Figure S41: ^{13}C NMR spectrum of **18** in $CDCl_3$

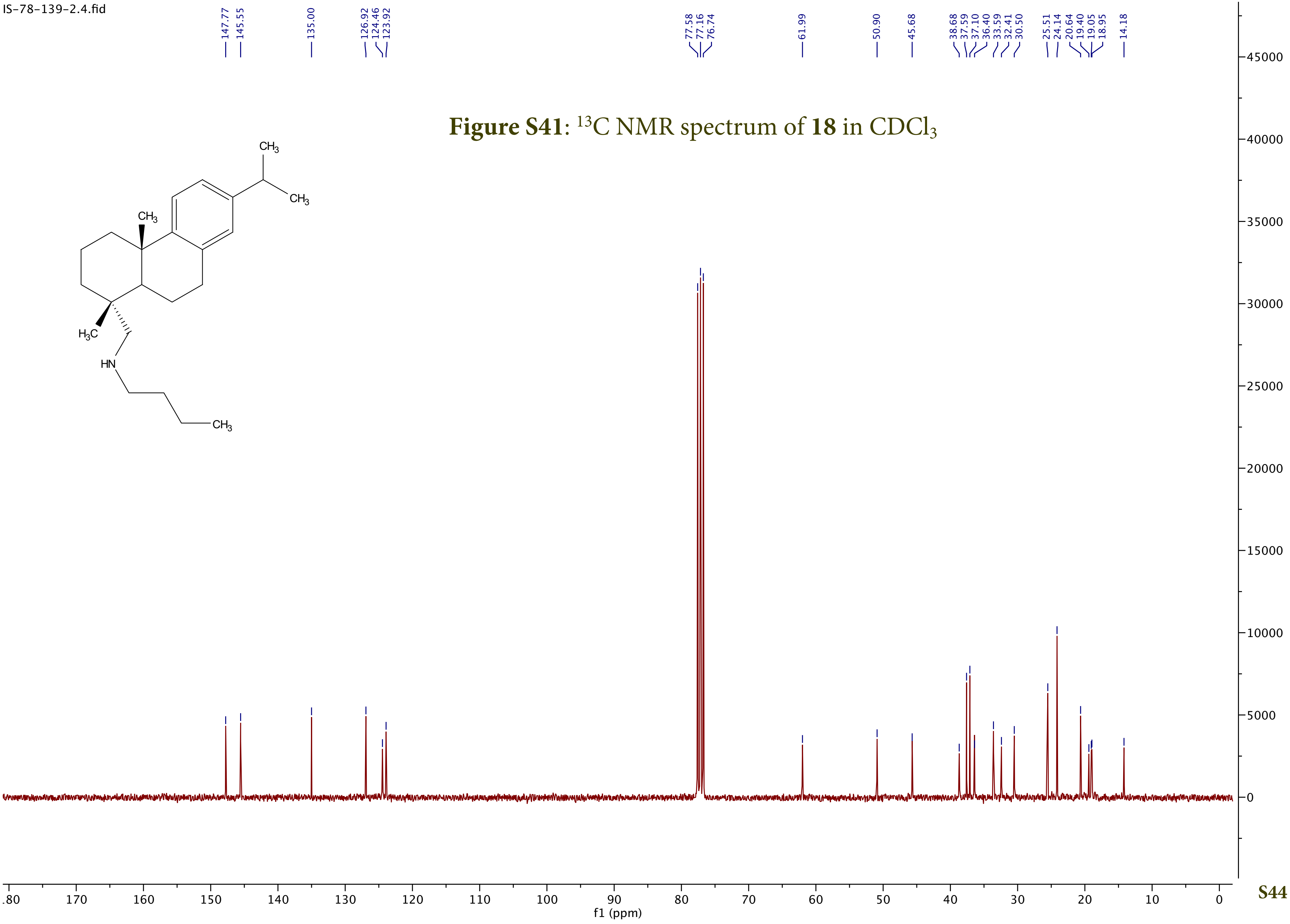

Figure S42: High resolution mass spectrum of **18**

Sample Name	Mol Fomula	MW	M+H	observed	delta	ppm
IS-78-139-2	C24H39N	341.3083	342.3161	342.3155	-0.0005	-1.61

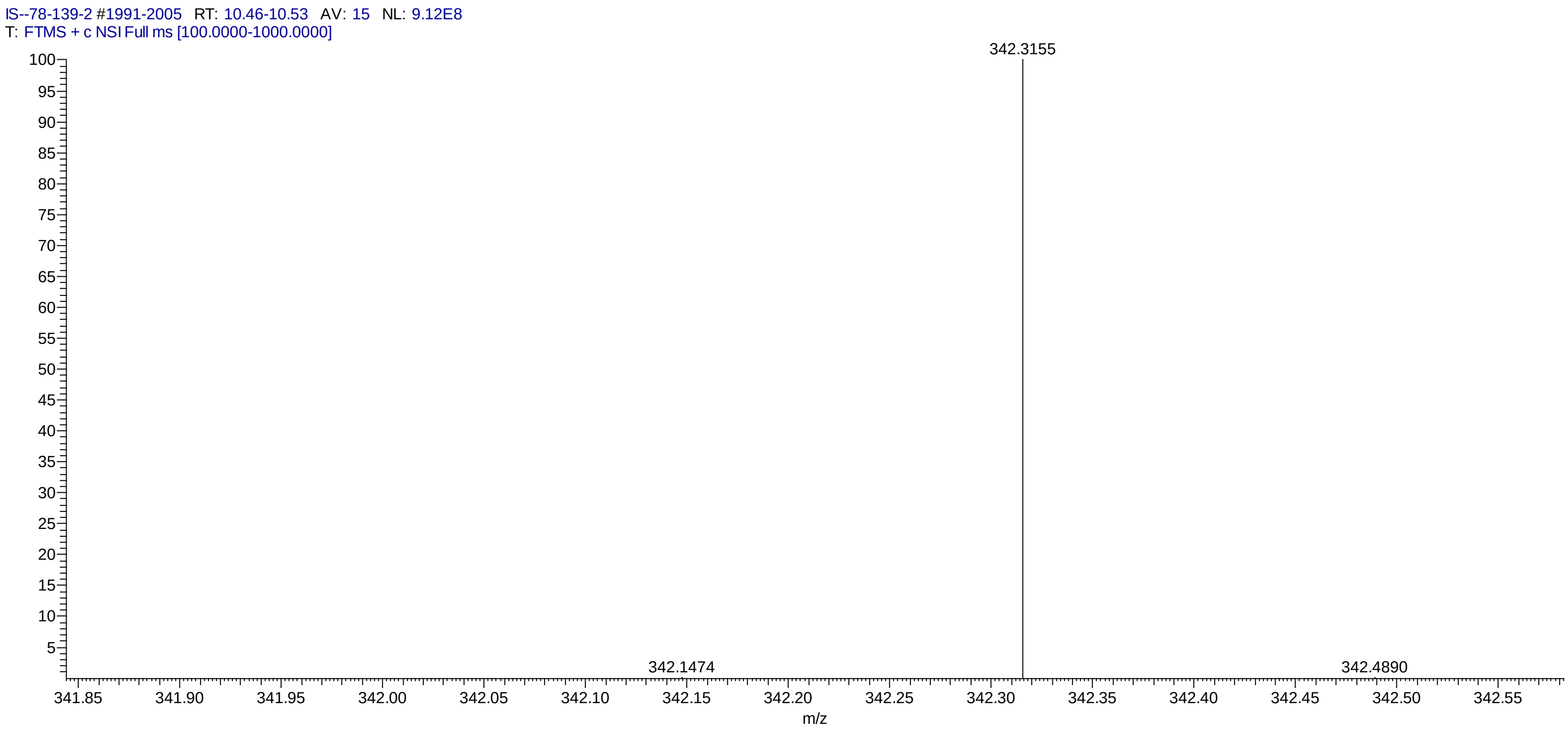

Figure S43: ^{1}H NMR spectrum of **19** in $CDCl_3$

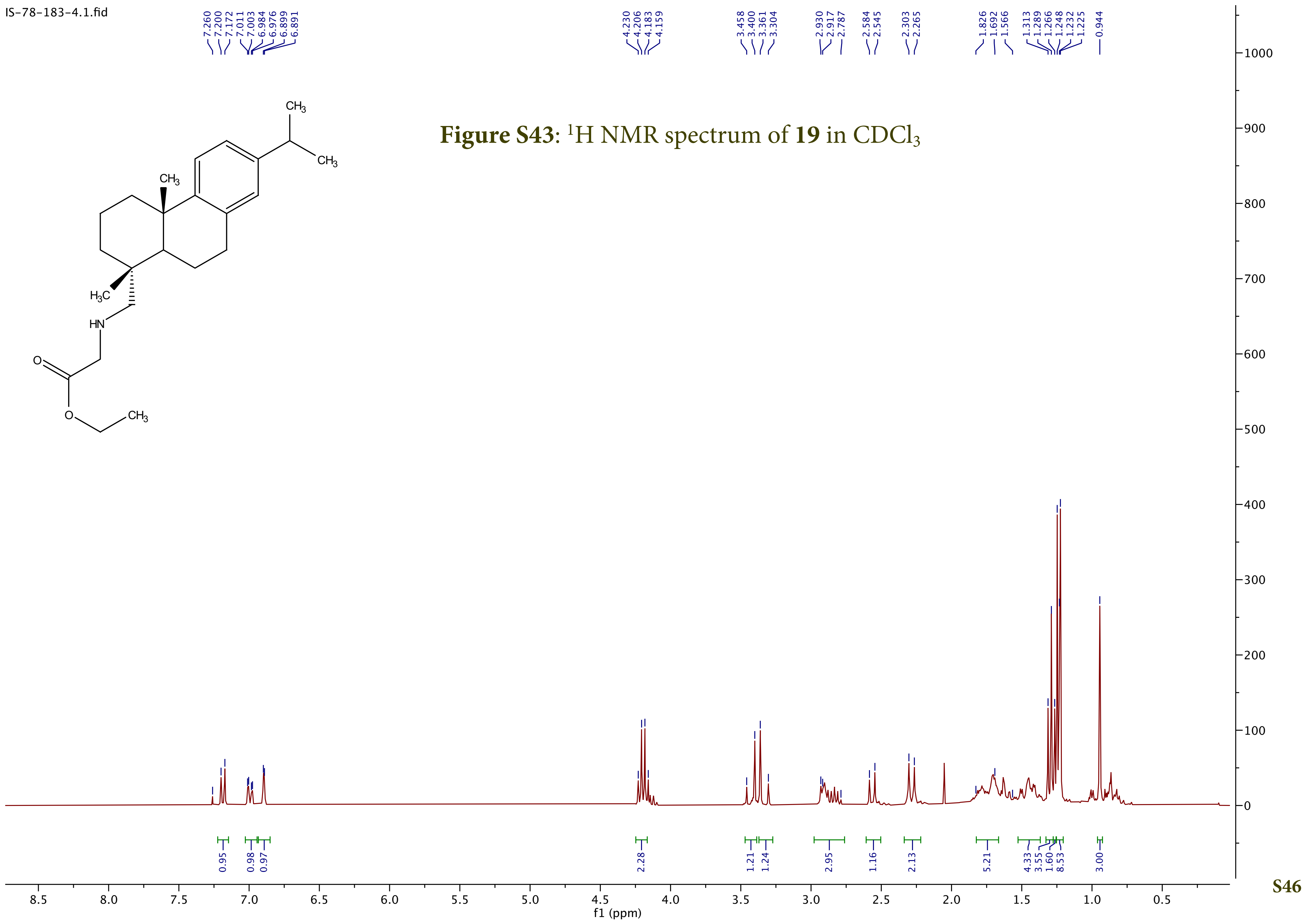

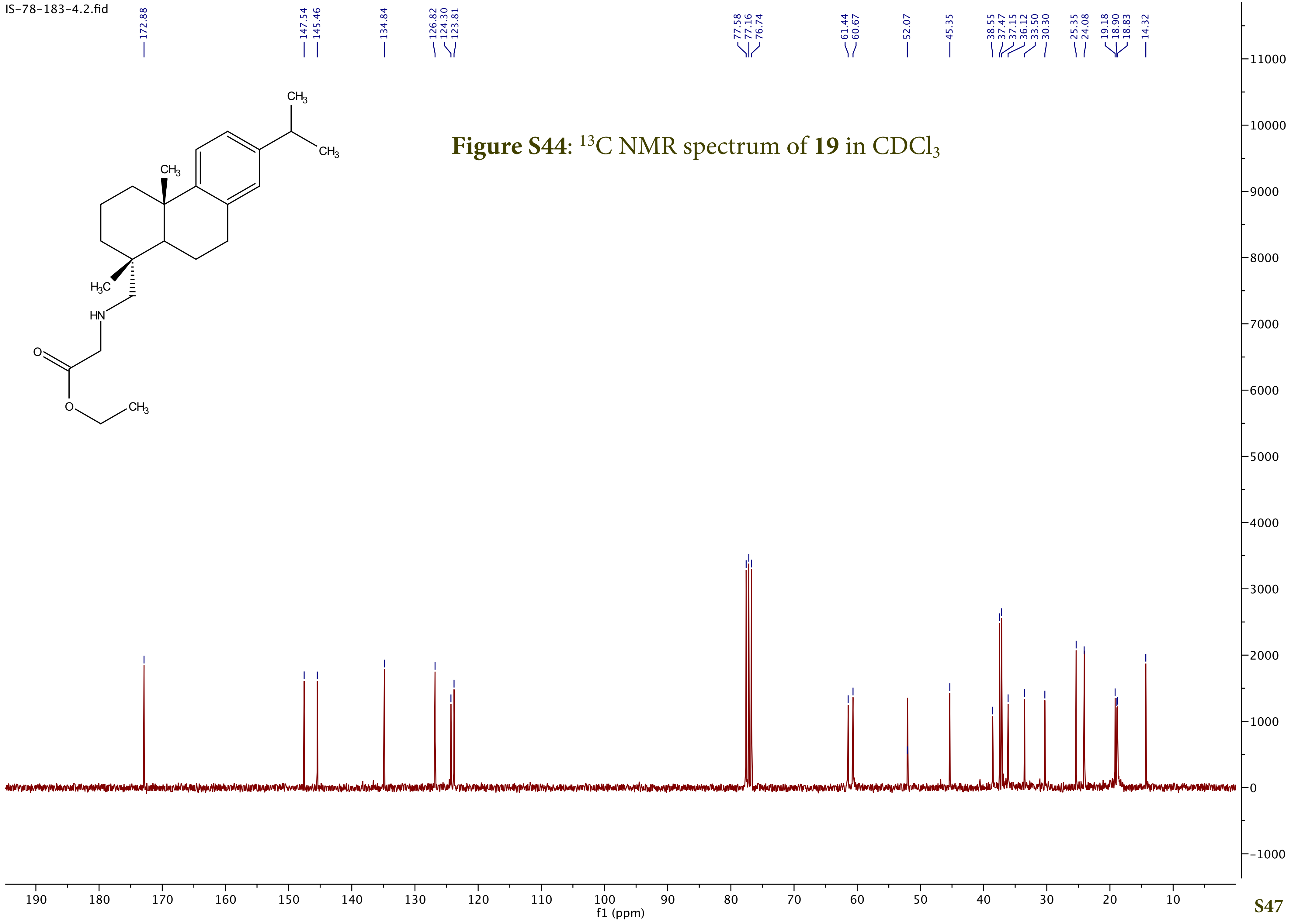

Figure S44: ^{13}C NMR spectrum of **19** in $CDCl_3$

Figure S45: High resolution mass spectrum of **19**

Sample Name	Mol Fomula	MW	M+H	observed	delta	ppm
IS-78-183-4	C24H37NO2	371.2824	372.2902	372.2896	-0.0006	-1.69

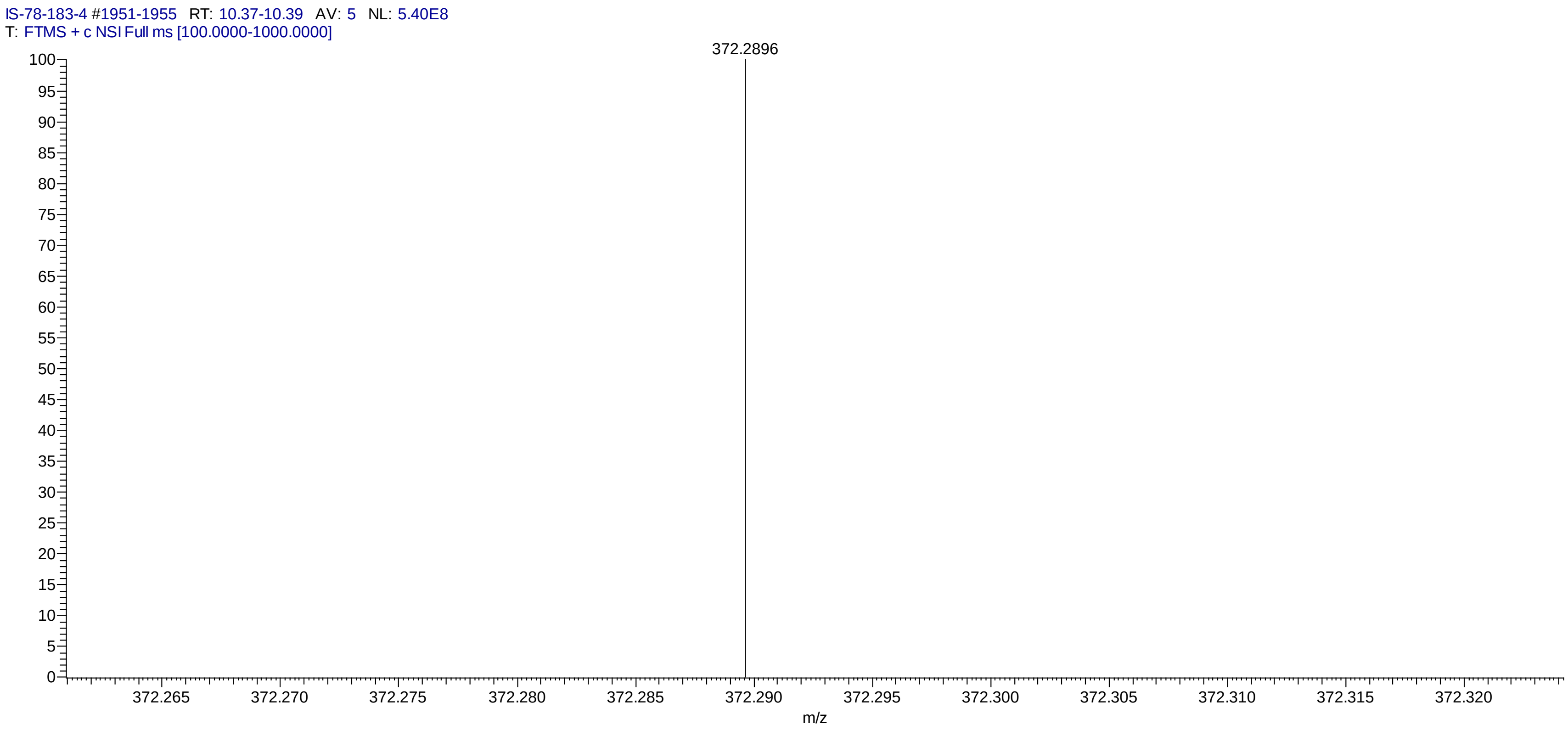

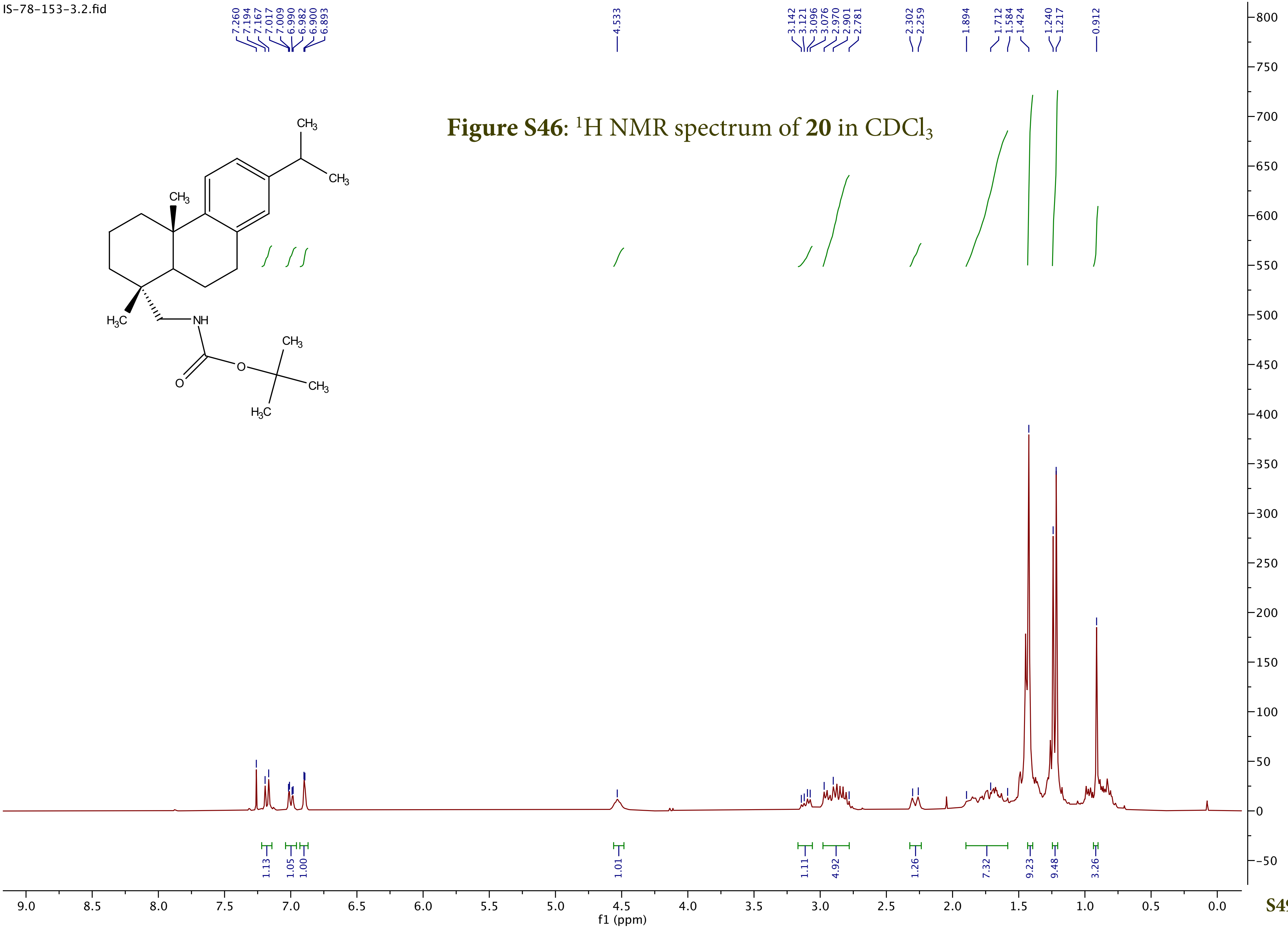

Figure S46: ^{1}H NMR spectrum of **20** in $CDCl_3$

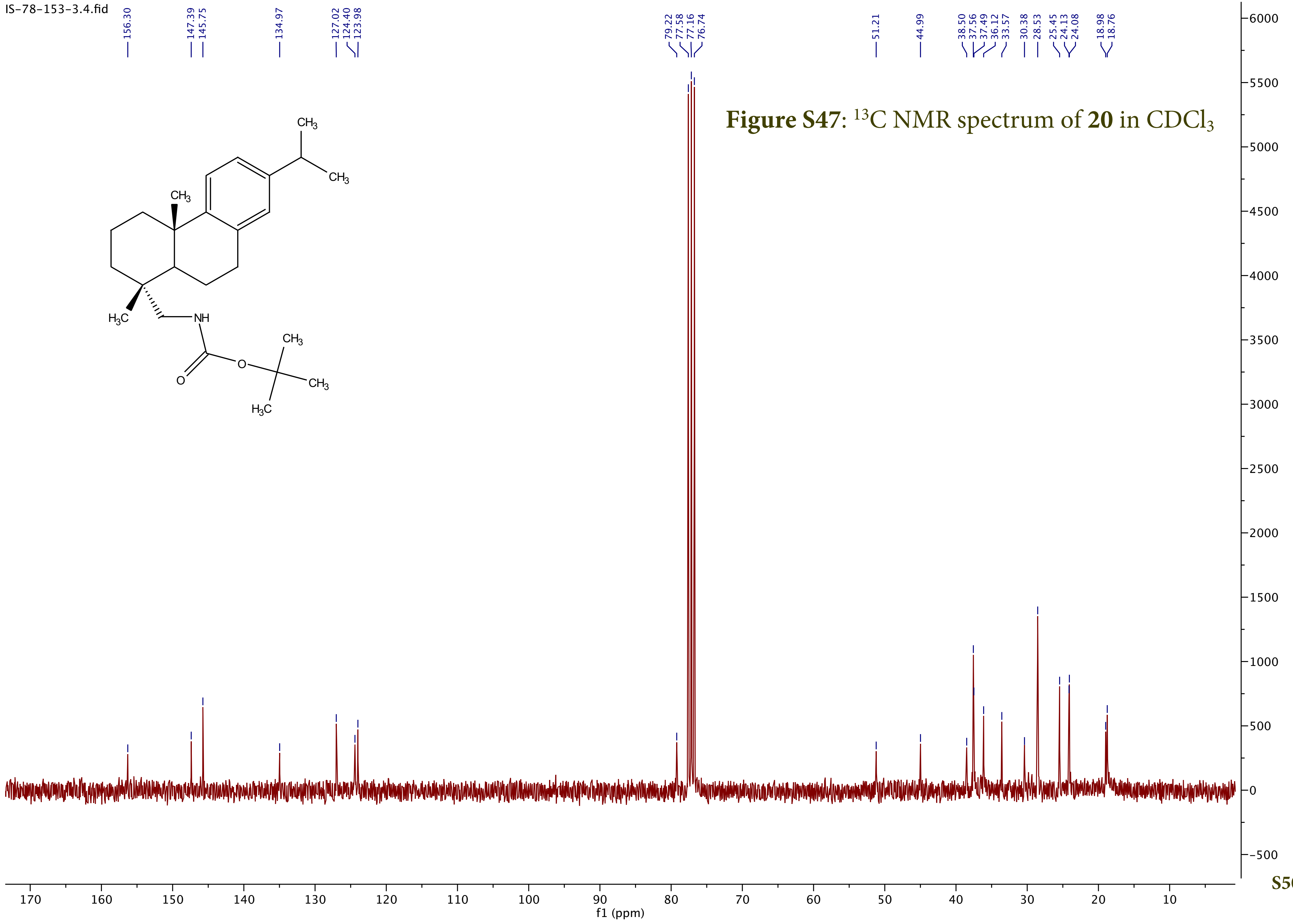

Figure S47: ^{13}C NMR spectrum of **20** in $CDCl_3$

Figure S48: High resolution mass spectrum of **20**

Sample Name	Mol Fomula	MW	M+H	observed	delta	ppm
IS-78-153(IS-78-153-3)	C25H39NO2	385.2981	386.3059	386.3051	-0.0008	-2.05

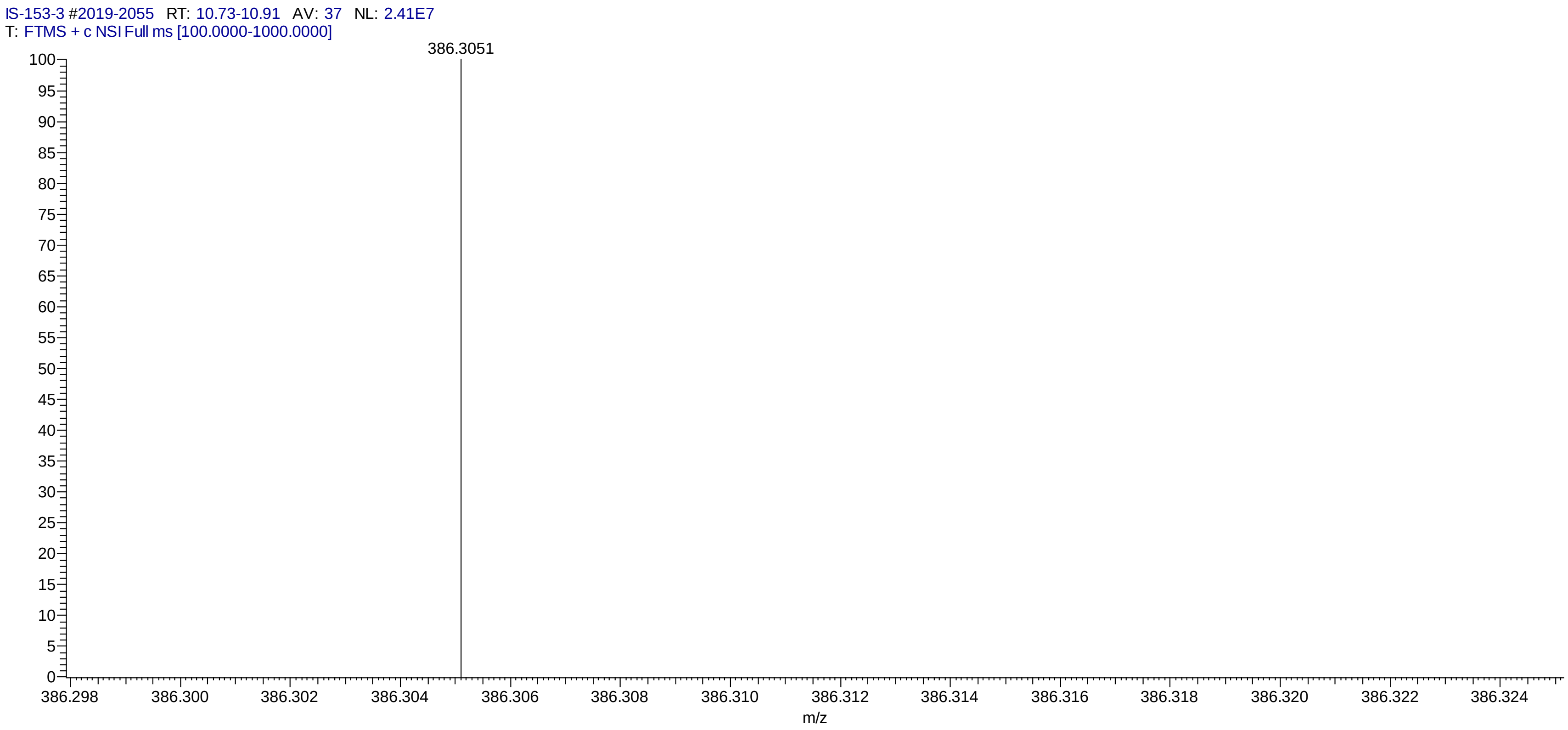

Figure S49: ^{1}H NMR spectrum of **21** in $CDCl_3$

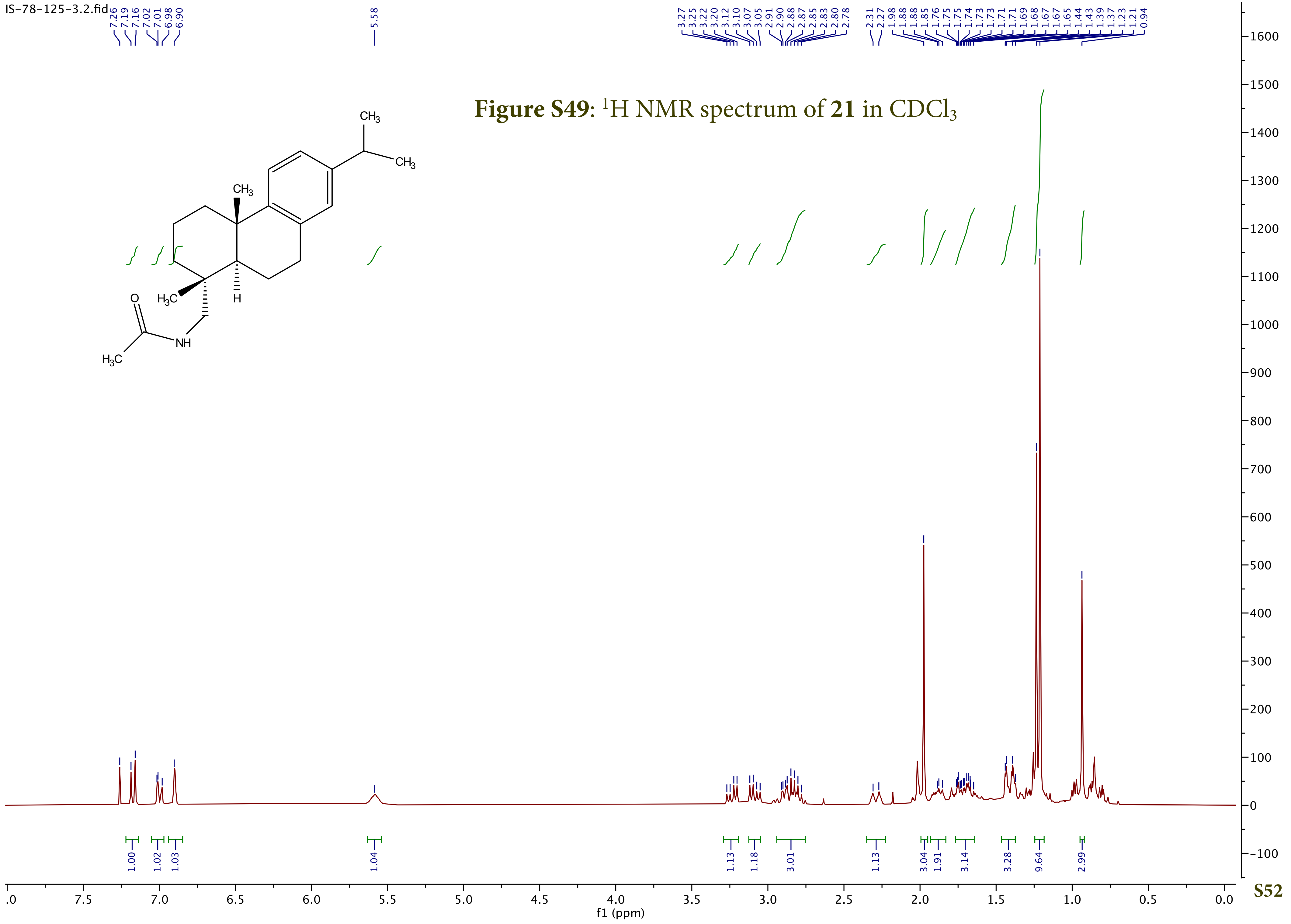

IS-78-125-3.4.fid

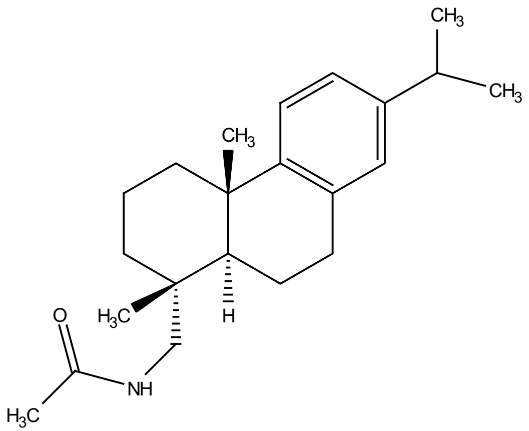

Figure S50: ^{13}C NMR spectrum of **21** in $CDCl_3$

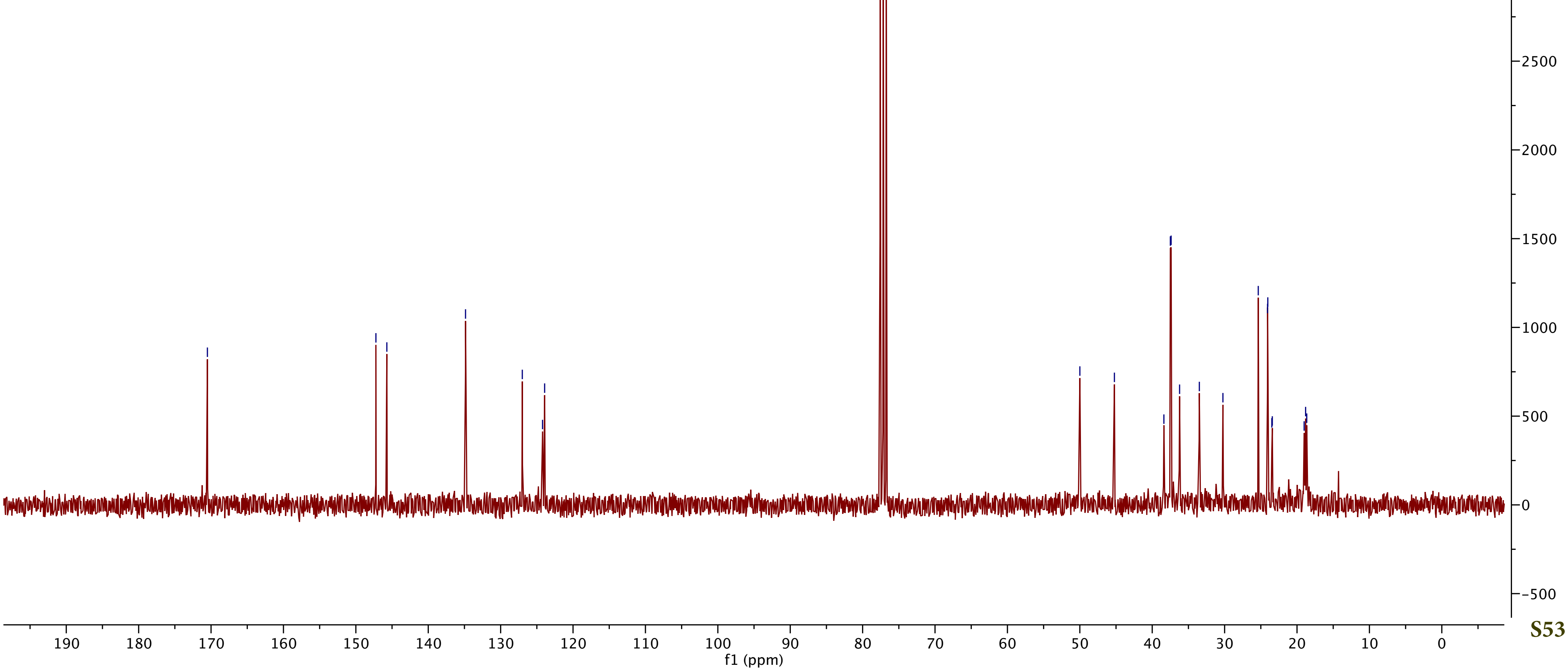

Figure S51: High resolution mass spectrum of **21**

Sample Name	Mol Fomula	MW(COME WITH SAMPLES)	MW(CALCULATED)	M+H	observed	delta	ppm
**IS-78-125-3**	C22H33NO	328.264	327.25621	328.2640	**328.2639**	-0.0001	-0.34

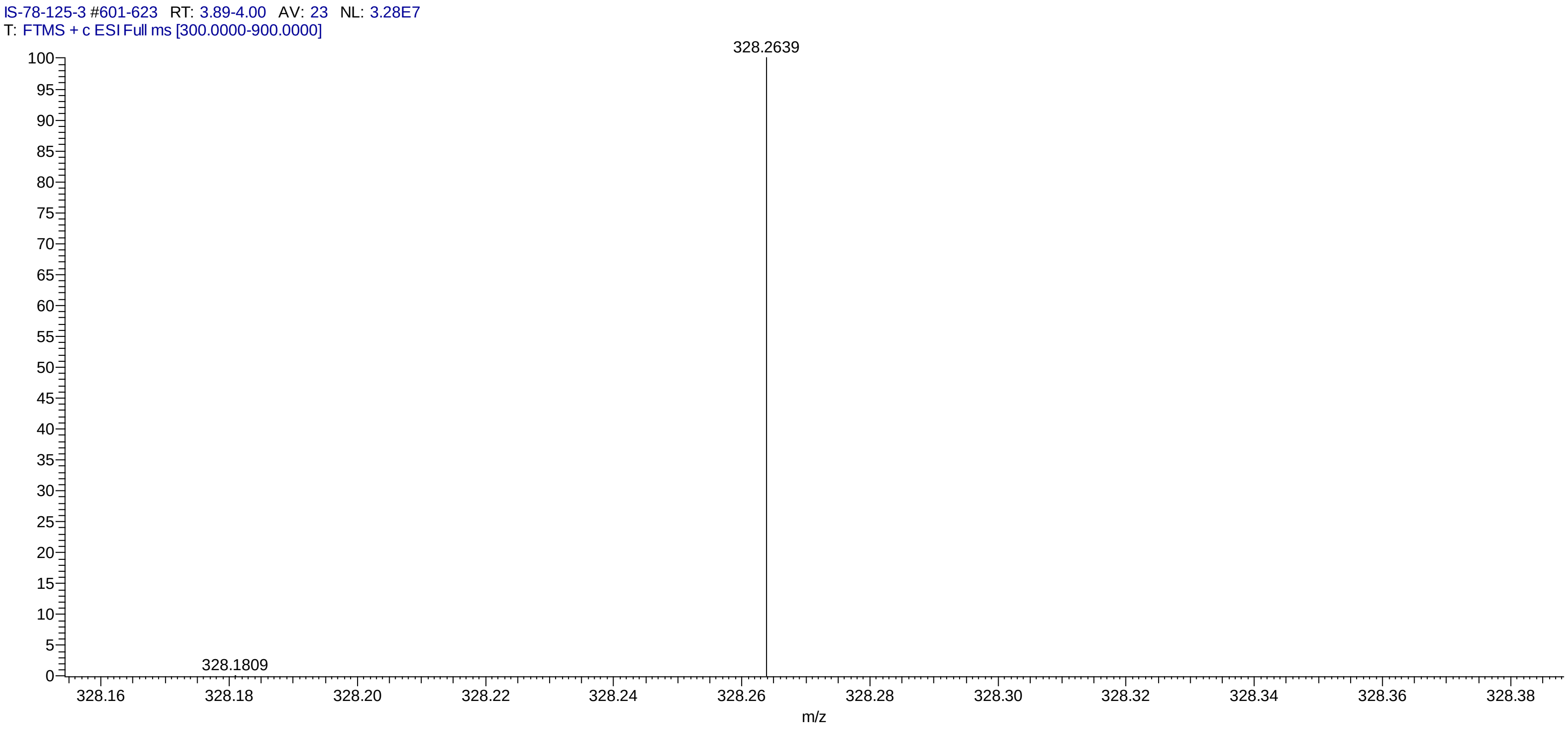

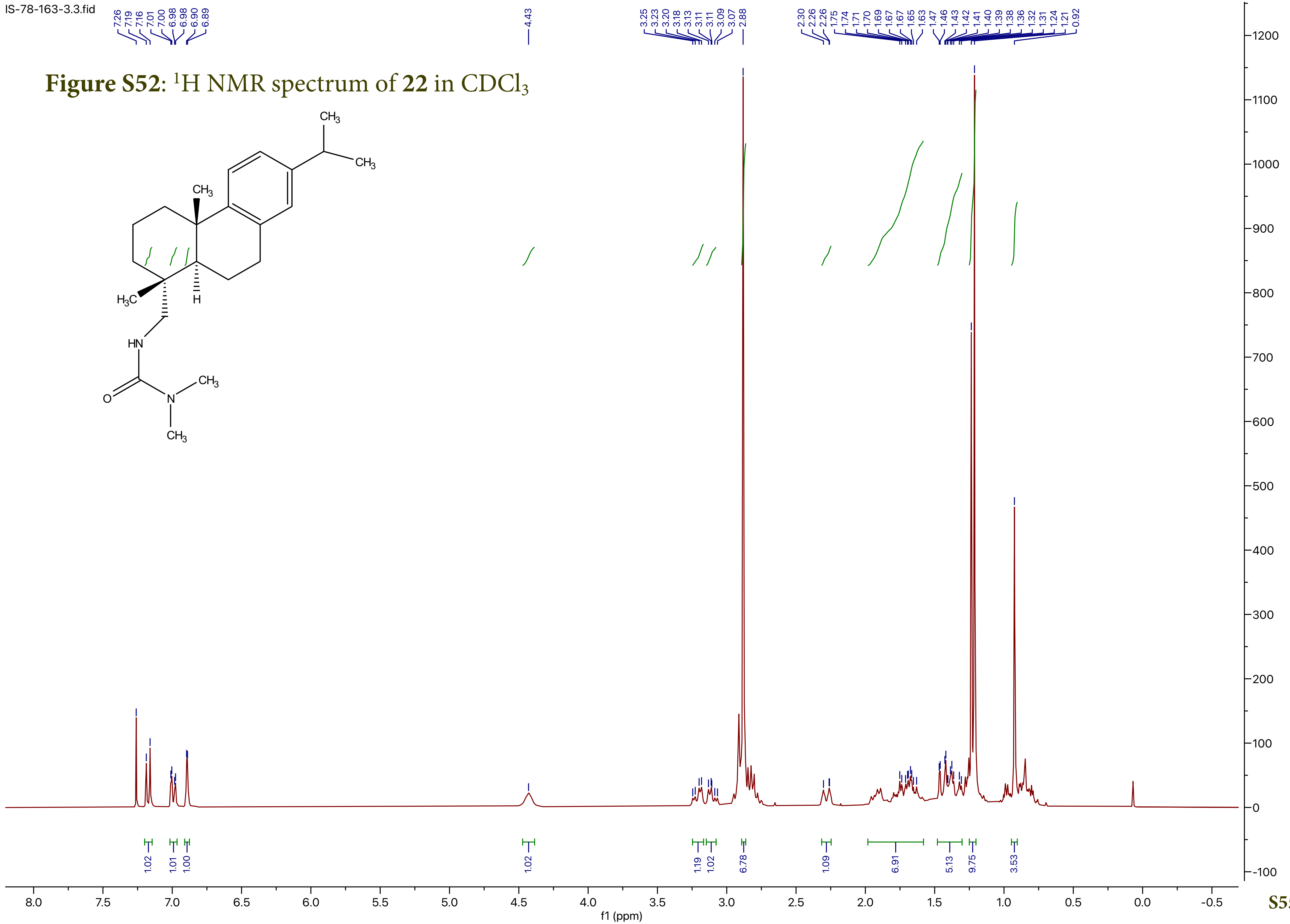

Figure S52: ^{1}H NMR spectrum of **22** in $CDCl_3$

Figure S53: ^{13}C NMR spectrum of **22** in $CDCl_3$

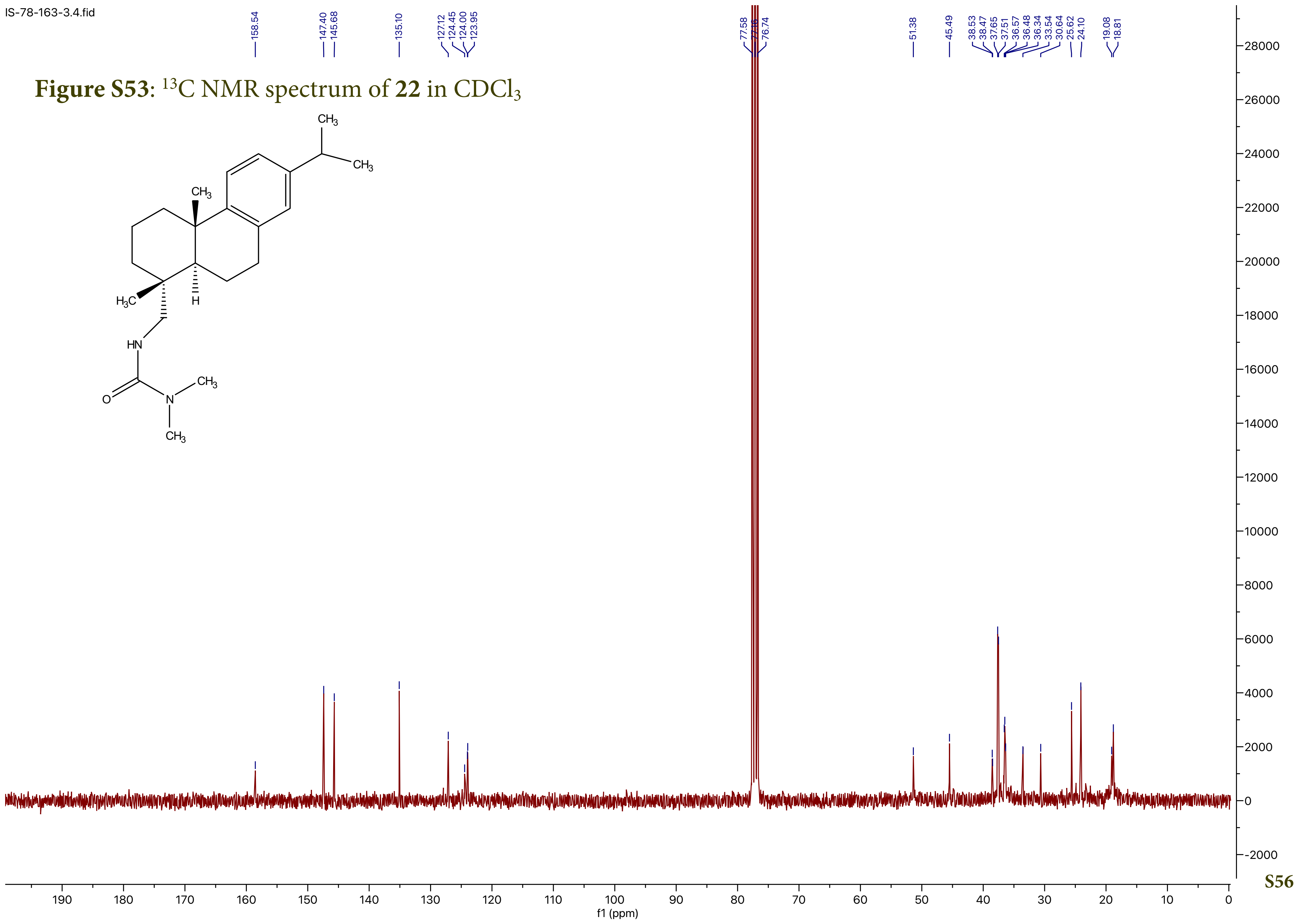

Figure S54: High resolution mass spectrum of **22**

Sample Name	Mol Fomula	MW	M+H	observed	delta	ppm
IS-78-163-3(IS-78-163-2)	C23H36N2O	356.2828	357.2906	357.2902	-0.0004	-1.04

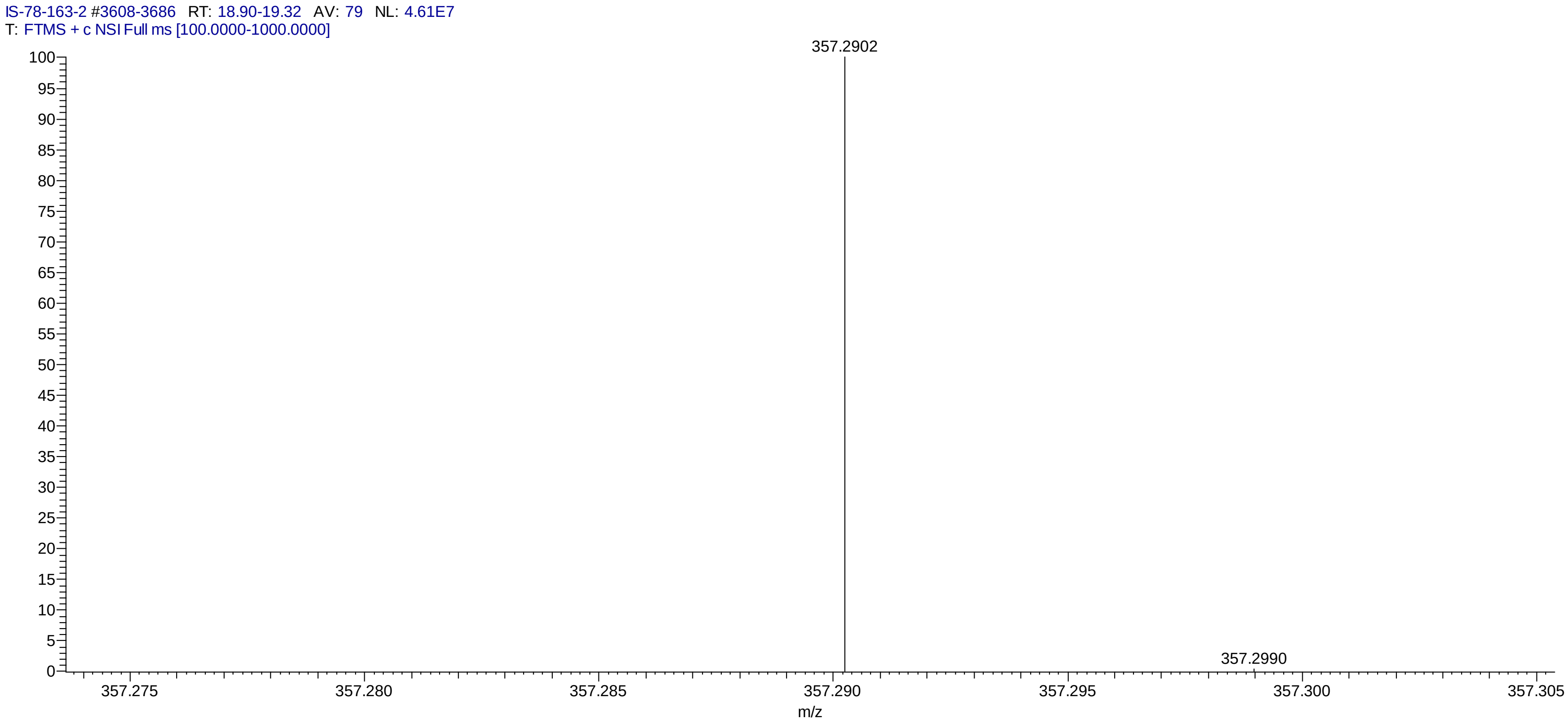

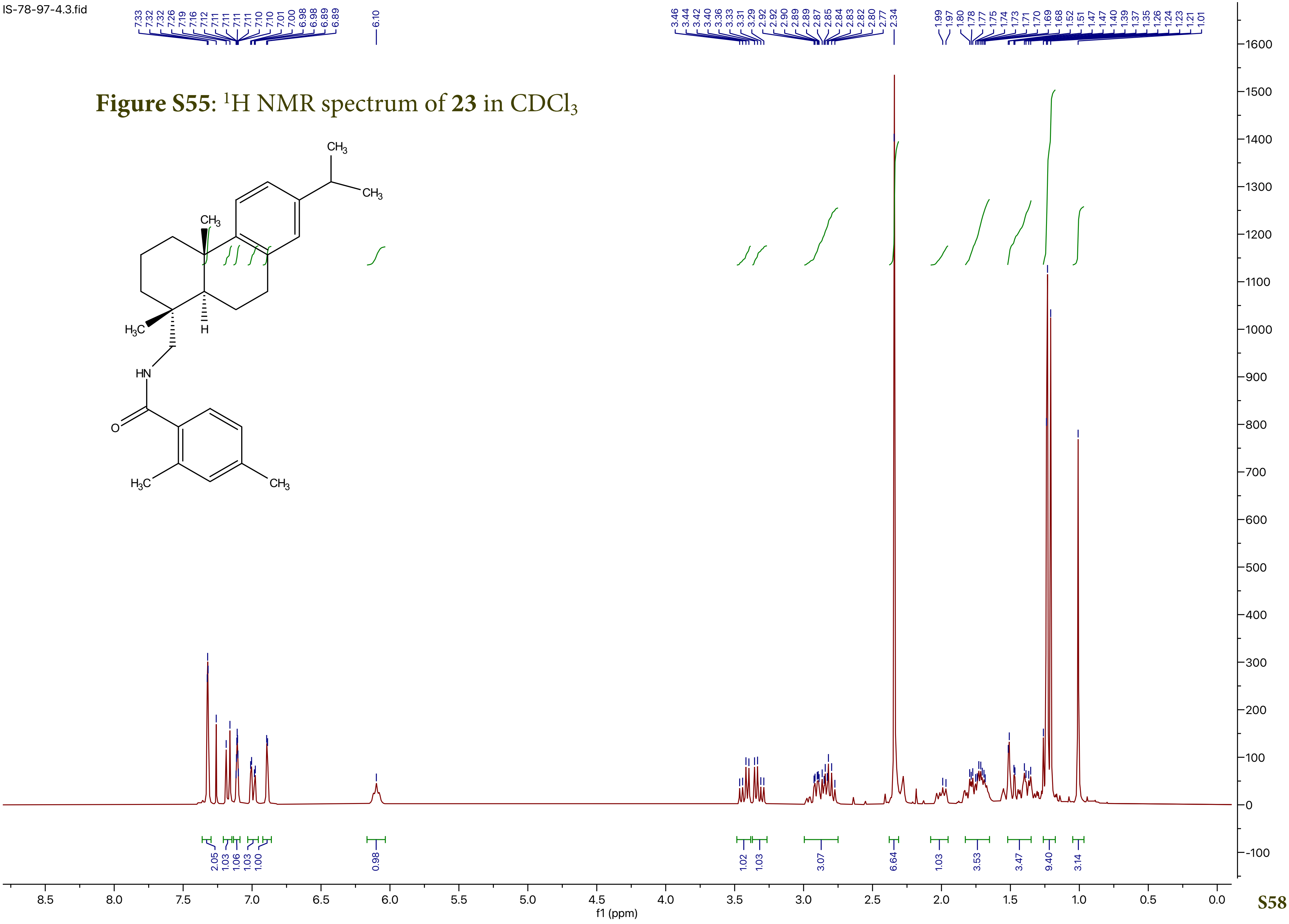

Figure S55: ^{1}H NMR spectrum of **23** in $CDCl_3$

IS-78-97-4.5.fid

Figure S56: ^{13}C NMR spectrum of **23** in $CDCl_3$

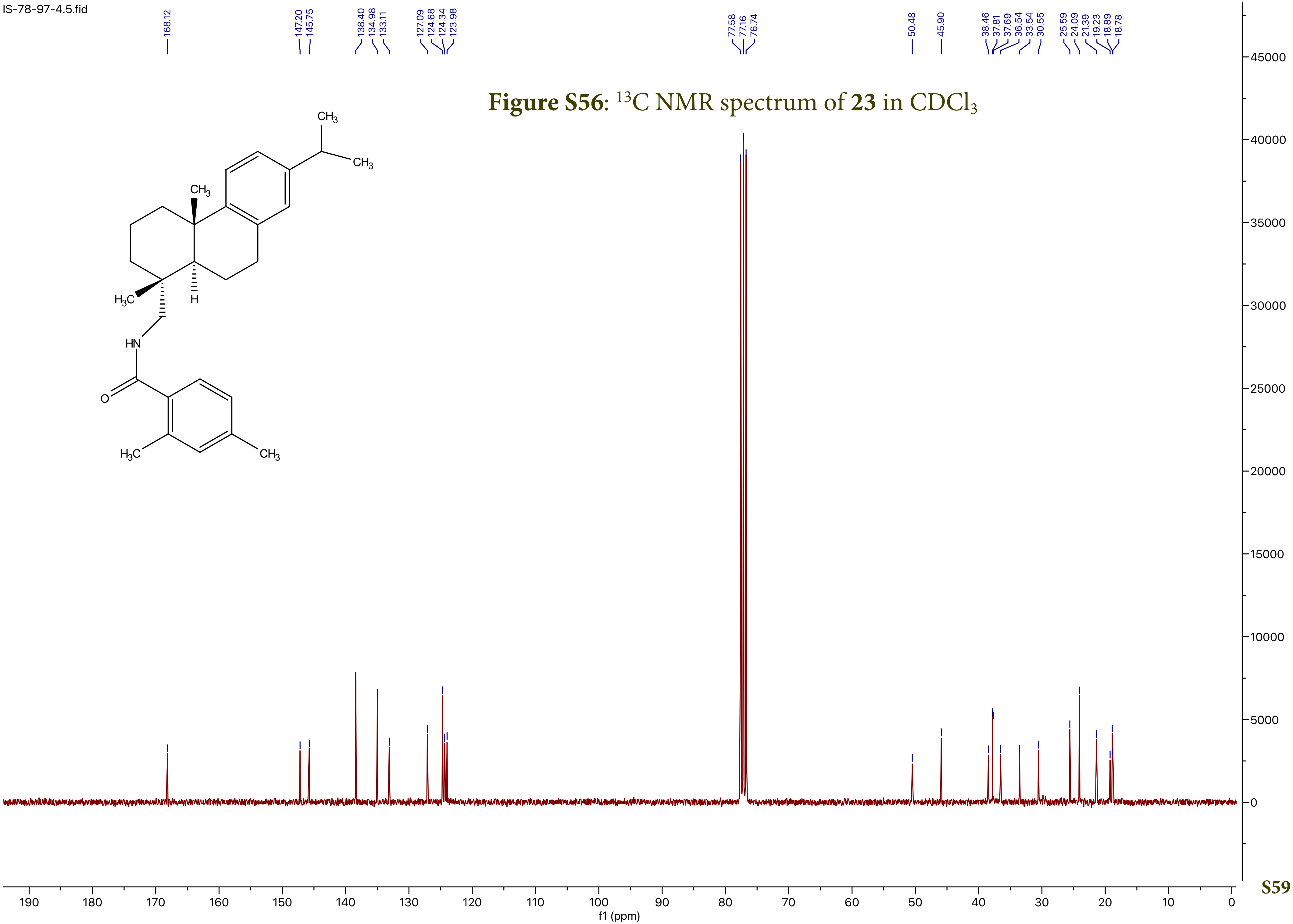

Figure S57: High resoluton mass spectrum of **23**

Sample Name	Mol Fomula	MW(COME WITH SAMPLES)	MW(CALCULATED)	M+H	observed	delta	ppm
**IS-78-97**	C29H39NO	417.3032	417.3032	418.3110	**418.3109**	-0.0001	-0.17

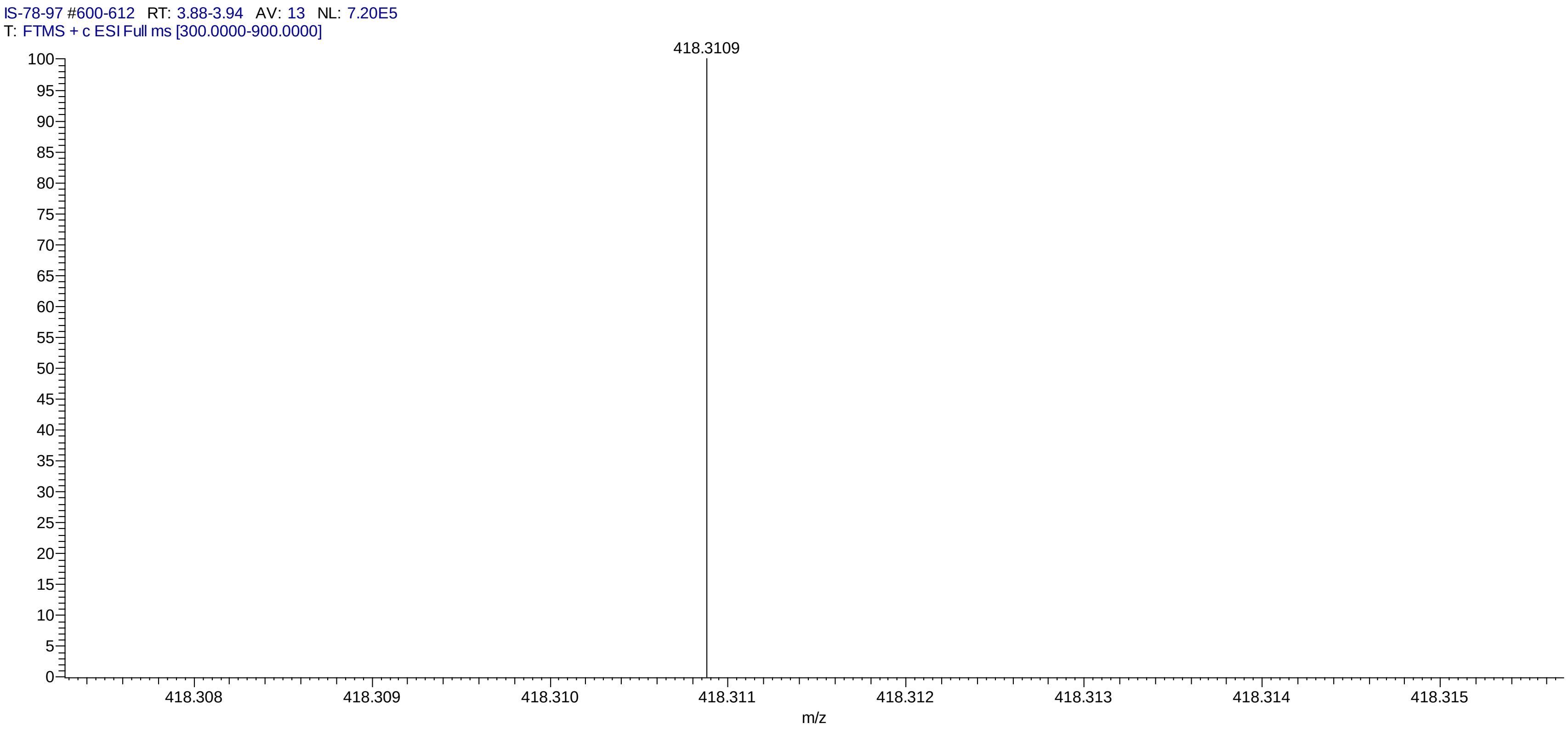

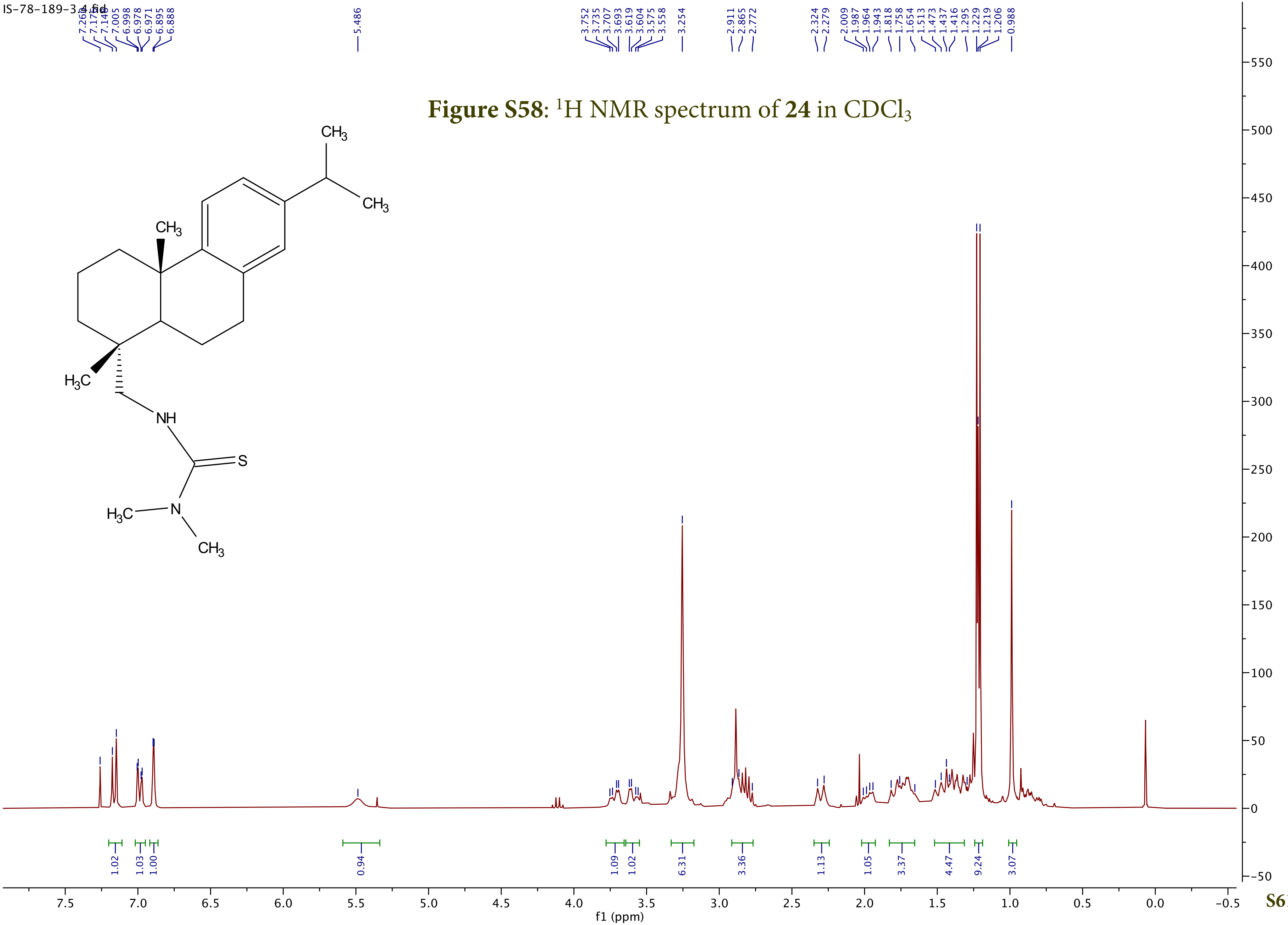

Figure S58: ^{1}H NMR spectrum of **24** in $CDCl_3$

Figure S59: ^{13}C NMR spectrum of **24** in $CDCl_3$

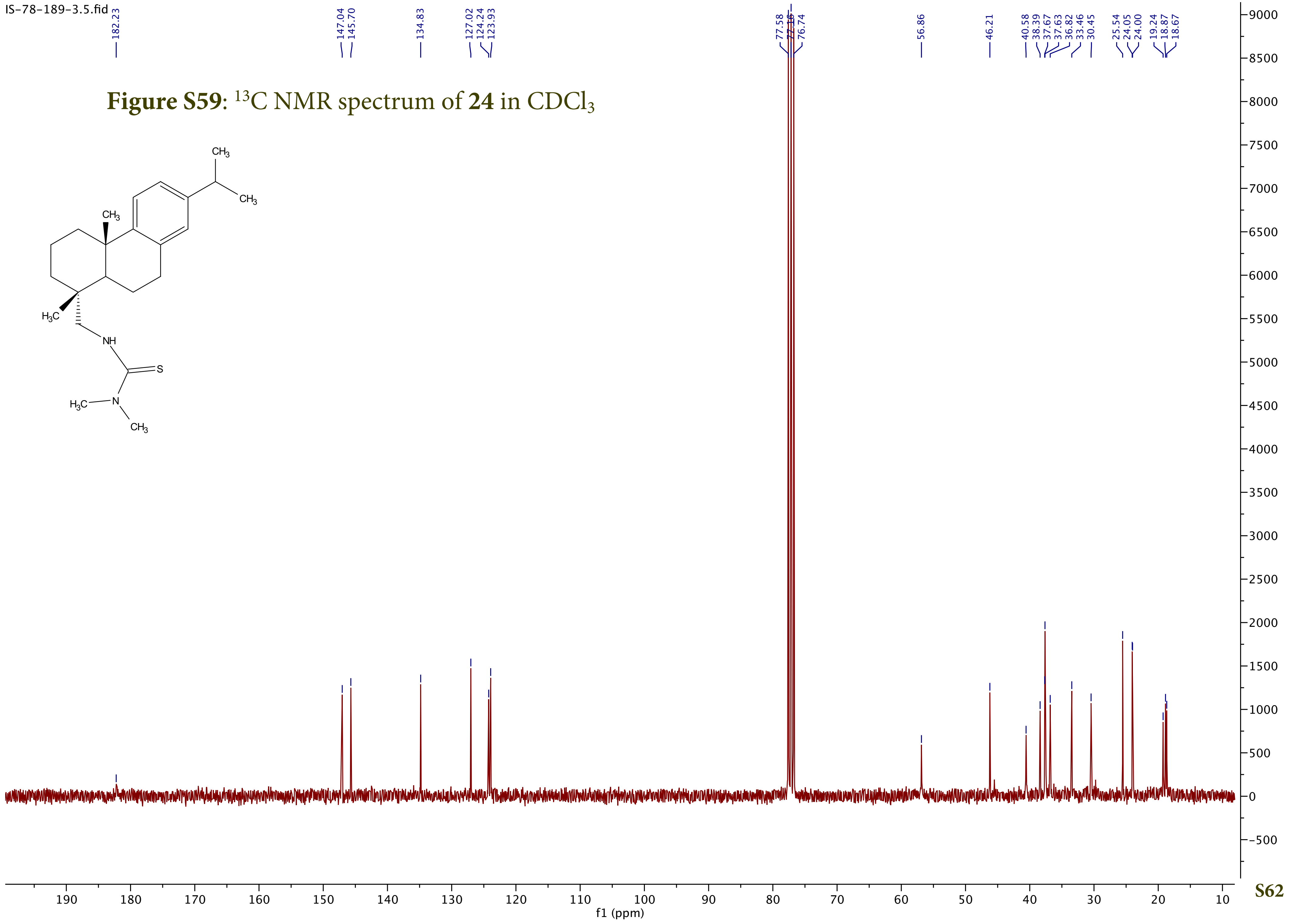

Figure S60: High resolution mass spectrum of **24**

Sample Name	Mol Fomula	MW	M+H	observed	delta	ppm
IS-78-189-3	C23H36N2S	372.2599	373.2677	373.2675	-0.0002	-0.59

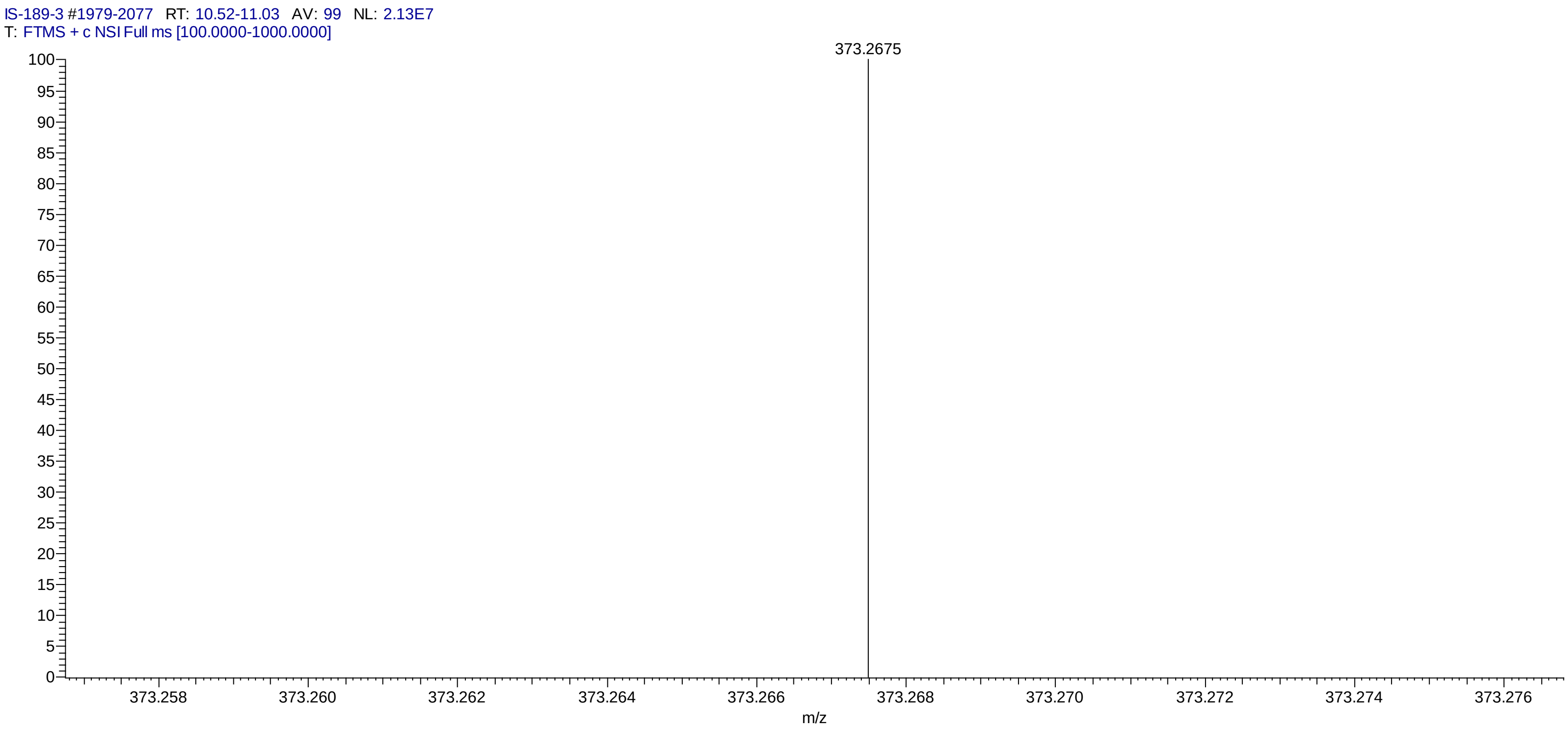

Supplement: Supplementary file 1 [file molecules-28-04743-s001.zip › molecules-2453093-supplementary.pdf]
